# MiR-125b Reduces Porcine Reproductive and Respiratory Syndrome Virus Replication by Negatively Regulating the NF-κB Pathway

**DOI:** 10.1371/journal.pone.0055838

**Published:** 2013-02-07

**Authors:** Dang Wang, Lu Cao, Zheng Xu, Liurong Fang, Yao Zhong, Quangang Chen, Rui Luo, Huanchun Chen, Kui Li, Shaobo Xiao

**Affiliations:** 1 Division of Animal Infectious Diseases, State Key Laboratory of Agricultural Microbiology, College of Veterinary Medicine, Huazhong Agricultural University, Wuhan, China; 2 Department of Microbiology, Immunology and Biochemistry, University of Tennessee Health Science Center, Memphis, Tennessee, United States of America; Huazhong Agricultural University, China

## Abstract

Porcine reproductive and respiratory syndrome virus (PRRSV) is an Arterivirus that has been devastating the swine industry worldwide since the late 1980s. To investigate the impact of cellular microRNAs (miRNAs) on the replication of PRRSV, we screened 10 highly conserved miRNAs implicated in innate immunity or antiviral function and identified miR-125b as an inhibitor of PRRSV replication. Virus titer and western blot assays demonstrated that miR-125b reduced PRRSV replication and viral gene expression in a dose-dependent manner in both MARC-145 cell line and primary porcine alveolar macrophages. Mechanistically, miR-125b did not target the PRRSV genome. Rather, it inhibited activation of NF-κB, which we found to be required for PRRSV replication. PRRSV, in turn, down-regulated miR-125b expression post-infection to promote viral replication. Collectively, miR-125b is an antiviral host factor against PRRSV, but it is subject to manipulation by PRRSV. Our study reveals an example of manipulation of a cellular miRNA by an arterivirus to re-orchestrate host gene expression for viral propagation and sheds new light on targeting host factors to develop effective control measures for PRRS.

## Introduction

MicroRNAs (miRNAs, miRs) are small RNA molecules that regulate gene expression at the post-transcriptional level [1]. In mammals, miRNAs are initially transcribed by RNA polymerase II and the primary miRNA transcripts (pri-miRNAs) are sequentially cut by RNase III enzymes Drosha and Dicer [2], [3]. The resulting ∼23-nucleotide double-stranded mature miRNA molecules load into RNA-induced silencing complexes (RISC) [4], [5], where they act to repress mRNA translation or reduce mRNA stability by interacting with miRNA-recognition elements (MRE) within 3′ untranslated region (UTR) of target genes [6]. The specificity of miRNAs is thought to be primarily mediated by residues 2–8 at the 5′ end of miRNA, also known as the seed region [7].

Growing evidence indicates that miRNAs play important roles in regulating viral infections [8]–[12]. Viral miRNAs may directly regulate viral and/or host cell gene expression to benefit the virus, and cellular miRNAs can also influence viral replication and pathogenesis [13]–[16]. As examples, the liver-specific miR-122 promotes the replication of hepatitis C virus (HCV) [17], [18], while miR-196, miR-199, miR-296, miR-351, miR-431 and miR-448 inhibit HCV genome propagation [19], [20]; miR-32 effectively restricts the accumulation of primate foamy virus type 1 (PFV-1) in human cells [21]; miR-323, miR-491 and miR-654 inhibit the replication of the H1N1 influenza A virus by binding to the viral *PB1* gene [22]; miR-28, miR-125b, miR-150, miR-223 and miR-382 target the 3′ end of human immunodeficiency virus (HIV) mRNA, thereby restricting HIV production [23]; miR-199a-3p and miR-210 limit the hepatitis B virus (HBV) surface antigen and polymerase production by degrading and/or inhibiting translation of viral mRNAs encoding these proteins [24]; overexpression of miR-24 and miR-93 suppresses vesicular stomatitis virus (VSV) replication through targeting the viral genes encoding RNA-dependent RNA polymerase (L protein) and phosphoprotein (P protein), respectively [25]; in macrophages, upregulation of miR-155 suppresses VSV replication, while inhibition of miR-155 had the opposite effect. Interestingly, instead of directly acting on VSV RNA, miR-155 was shown to target the expression of SOCS1, a negative regulator of type I interferon signaling, thereby indirectly enhancing the anti-viral state of the cell [26]. Defining the functions of miRNAs in regulating viral replication and pathogenesis may help identify new therapeutic approaches against viral diseases.

Porcine reproductive and respiratory syndrome (PRRS) is an emerging viral infectious disease characterized by severe reproductive failure in sows and respiratory distress in piglets and growing pigs [27]. The causative agent, PRRS virus (PRRSV), is a single-stranded positive-sense RNA virus classified within the family *Arteriviridae*. Since its emergence in the late 1980s, PRRS has continuously been a threat to the global swine industry, causing high economic losses [28], [29]. Unfortunately, neither traditional control strategies nor conventional vaccines provide sustainable control of PRRS [29]–[31]. A major obstacle in the development of a successful PRRS vaccine is the unconventional immune response of pigs to the virus [32]–[34], which remains poorly characterized. A better understanding of the virus-host interactions in PRRSV infection will facilitate development of more effective control measures [35]–[40]. Currently, the role of cellular miRNAs in PRRSV replication is unclear.

To determine whether specific cellular miRNA(s) regulates PRRSV propagation, we screened the synthetic mimics or inhibitors of 10 miRNAs which are well-conserved among different host species and were previously implicated in innate immunity and/or reported to possess antiviral activity against other viruses. Our results revealed that miR-125b is an inhibitor of PRRSV replication. We also investigated the underlying mechanism(s) and found that miR-125b does not directly target the PRRSV genome but rather inhibits activation of NF-κB, which is required for optimal replication of PRRSV.

## Materials and Methods

### Cells, Reagents and Virus

MARC-145 cells, a monkey kidney cell line highly permissive for PRRSV infection, were purchased from the American Type Culture Collection (ATCC no. CRL-1223), and cultured in DMEM (Invitrogen) supplemented with 10% fetal bovine serum (FBS), 100 U/mL penicillin and 100 µg/mL streptomycin in a humidified 37°C/5% CO_2_ incubator. Porcine alveolar macrophages (PAMs) were isolated from 4- to 6-week-old conventional Landrace pigs from a PRRSV-negative herd as described by Wensvoort et al [41]. The mimics and inhibitors of miR-24, miR-93, miR-122, miR-125b, miR-146a, miR-155, miR-181, miR-196, miR-351, and miR-365 (shown in Table S1) were obtained from GenePharma (Shanghai, China). BAY11-7082, a specific pharmacological inhibitor of NF-κB, was purchased from Calbiochem-Merck (Darmstadt, Germany). Poly(I:C) was obtained from Sigma. The WUH3 strain of PRRSV (GenBank accession no. HM853673) was used throughout this study. This virus was isolated from the brain of pigs suffering from the “high fever” syndrome in China at the end of 2006 and identified as a highly pathogenic type 2 PRRSV [42]. VSV-EGFP, a recombinant vesicular stomatitis virus (VSV) expressing green fluorescent protein (GFP), was a gift from Dr. Z.G. Bu (Harbin Veterinary Research Institute of Chinese Academy of Agricultural Science).

### Plasmids

The pMIR-REPORT luciferase reporter vector (Ambion) was used as the cloning vector for reporter gene assay analyzing the potential target region of miR-125b in PRRSV genome. The 21 cDNA fragments corresponding to 5′ UTR, 3′ UTR, and 19 nonstructural and structural genes (nsp1α, nsp1β, nsp2-nsp5, nsp7-nsp12, ORF2a, ORF2b, ORF3-ORF7) of PRRSV were amplified by PCR from PRRSV RNA (WUH3 strain) and subcloned into the pMIR-REPORT vector downstream of the luciferase ORF, to generate the reporter vectors pMIR-5′UTR, pMIR-3′UTR, pMIR-nsp1α, pMIR-nsp1β, pMIR-nsp2, pMIR-nsp3, pMIR-nsp4, pMIR-nsp5, pMIR-nsp7, pMIR-nsp8, pMIR-nsp9, pMIR-nsp10, pMIR-nsp11, pMIR-nsp12, pMIR-ORF2a, pMIR-ORF2b, pMIR-ORF3, pMIR-ORF4, pMIR-ORF5, pMIR-ORF6, and pMIR-ORF7, respectively. The primers used are listed in Table S2. All cDNA constructs were verified by DNA sequencing.

pNF-κB-Luc was purchased from Stratagene, and IFN-β-Luc (p125-Luc) was kindly provided by T. Fujita (Laboratory of Molecular Genetics, Institute for Virus Research, Kyoto University, Kyoto, Japan). The cDNA expression construct for the p65 subunit of NF-κB has been described previously [43].

### Luciferase Reporter Gene Assay

The indicated plasmids and miRNA mimics or inhibitors were transfected into cells using Lipofectamine 2000 (Invitrogen, Carlsbad, CA) according to the manufacturer’s protocol. For luciferase reporter gene assay, subconfluent MARC-145 cells cultured in 24-well plates were co-transfected with 100 ng/well of the indicated reporter plasmid and 50 ng/well of pRL-TK (as an internal control to normalize the transfection efficiency, Promega), along with the indicated amount of miR-125b mimic or inhibitor. Cells were lysed 24 h later for determination of firefly luciferase and Renilla luciferase activities using the Dual-luciferase reporter assay system (Promega) as per manufacturer’s suggestions. Data are presented as relative firefly luciferase activity normalized to Renilla luciferase activity and are representative of three independently conducted experiments.

### Western Blotting

Briefly, MARC-145 cells were transfected with miR-125b mimic or the control mimic prior to PRRSV infection. Cells were collected at 30 h post-infection by adding 250 µL 2× lysis buffer A (LBA) (65 mM Tris-HCl [pH 6.8], 4% sodium dodecyl sulfate, 3% DL-dithiothreitol, and 40% glycerol). Cell lysates were then analyzed for expression of PRRSV nonstructural protein 2 (nsp2) by Western blotting using a specific monoclonal antibody (MAb) against PRRSV Nsp2 as the primary antibody (1∶1,000). The monoclonal antibody used for the detection of PRRSV Nsp2 was produced from hybridoma cells derived from Sp2/0 myeloma cells and spleen cells of BALB/c mice immunized with recombinant Nsp2 protein of PRRSV strain WUH3. Beta-actin was detected with an anti-beta-actin monoclonal antibody (MAb) (Beyotime, China) to demonstrate equal protein sample loading.

### Plaque Assay for Determination of PRRSV Titers

MARC-145 cells were transfected with the mimic or inhibitor of the indicated miRNAs. At 24 h post-transfection, cells were infected with PRRSV (WUH3 strain) at a multiplicity of infection (MOI) of 0.1. Cells were collected 48 h later and stored at −80°C until analysis of viral replication by plaque assay. Plaque assay was essentially performed as described previously [44]. Briefly, 95% confluent MARC-145 cells grown in six-well tissue culture plates were infected for 1 h with 10-fold serial dilutions (1000 µl) of PRRSV-containing samples. After three washes with PBS (pH 7.4), cells were overlaid with 1.8% (w/v) Bacto agar mixed 1∶1 with 2×DMEM containing 0.05 mg/ml neutral red. Plaques were counted 4 days post-infection. The average plaque number and standard deviations were calculated from three independent experiments. Virus titers were expressed as plaque forming units (PFU)/mL.

### qPCR for miRNA Quantification

A commercial Bulge-Loop™ miRNA qRT-PCR Kit was purchased from Ribobio Co. (Guangzhou, China) and used for measuring miRNA abundance. Briefly, MARC-145 cells infected with PRRSV at a MOI of 0.1 were collected at the indicated time points and total RNA was extracted using TRIzol reagent (Invitrogen). Two micrograms of this total RNA were then used for programming reverse transcription using a miR-125b specific RT-primer. The abundance of the miRNA of interest in the resulting cDNA was determined by qPCR using a universal reverse primer and a miRNA-specific forward primer. The PCR procedure comprised pre-denaturation at 95°C for 2 min, and 40 cycles of 94°C for 10 seconds, 58°C for 15 seconds and 72°C for 20 seconds. The ubiquitously expressed U6 small nuclear RNA was used for normalization purpose. All primers used for qPCR were included in the commercial kit.

### Confocal Microscopy

The PAMs were transfected with miR-125b mimic or NC mimic (60 nM), followed by PRRSV infection (MOI = 0.1). Cells were fixed at 24 h post-infection and subsequently immunostained with a fluorescein isothiocyanate (FITC)-conjugated monoclonal antibody against the PRRSV N protein (SR30-F, Rural Technologies). Cellular nuclei were counterstained with 1 µg/mL of 4′,6′-diamidino-2-phenylindole (DAPI) for 5 min. After washing with PBS, cells were examined under an LSM 510 Meta confocal laser scanning microscope (Carl Zeiss, Göttingen, Germany).

### Statistical Analysis

All experiments were performed at least three times with reproducible results. Data are presented as mean ± standard deviation (SD). Student’s t-test was used to analyze the difference between two experimental groups. A *P*-value of <0.05 was considered statistically significant and a *P*-value of <0.01 was considered highly statistically significant.

## Results

### Identification of miR-125b as an Antiviral miRNA Against PRRSV Replication

**Figure 1:**
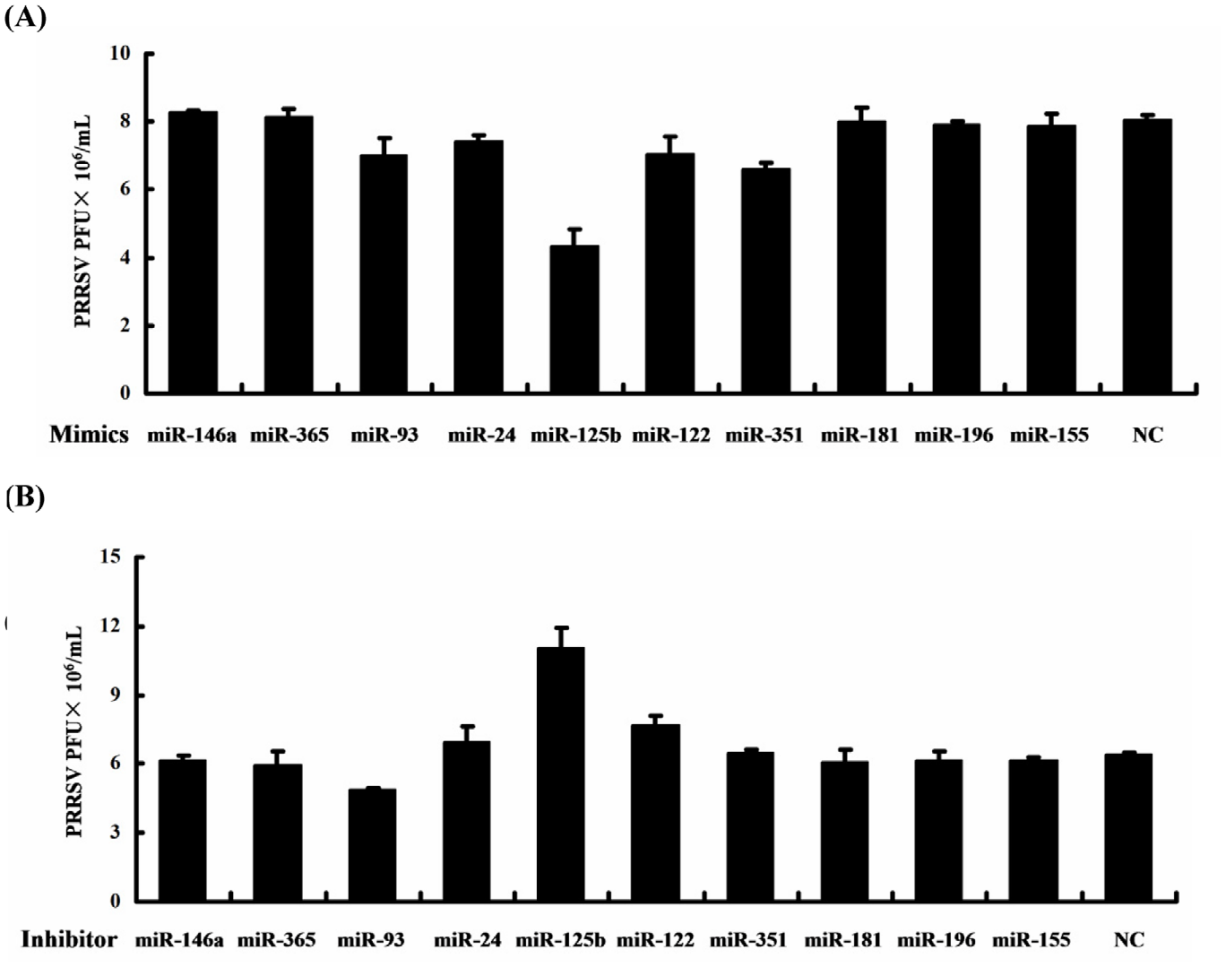
MicroRNA (miRNA) screening identifies miR-125b as an inhibitor of porcine reproductive and respiratory syndrome virus (PRRSV) replication. Confluent MARC-145 cells were transfected with the indicated mimics (A) or inhibitors (B) of the indicated miRNAs. 24 h later, cells were infected with PRRSV strain WUH3 at a multiplicity of infection (MOI) of 0.1. Cells were collected 48 h later for plaque assays on MARC-145 cells. Virus titers were expressed as plaque forming units (PFU)/mL.

To screen the potential miRNAs which can reduce PRRSV replication, the mimics or inhibitors of 10 miRNAs (Table S1), including miR-24, miR-93, miR-122, miR-125b, miR-146a, miR-155, miR-181, miR-196, miR-351, and miR-365, were chosen and synthetized. These miRNAs were selected because they are well-conserved among different host species and were implicated in innate immunity and/or antiviral function in previous studies [45], [46]. MARC-145 cells were transfected with the mimic or inhibitor of each miRNA (30 nM), followed by infection with PRRSV (WUH3 strain) at an MOI of 0.1. Cells were collected at 48 h post-infection to determine viral propagation (Figure 1). Among the miRNAs tested, the overexpression of miR-125b mimic significantly reduced progeny PRRSV production as determined by plaque assay (Figure 1A). Conversely, transfection of the miR-125b inhibitor demonstrated the opposite effects (Figure 1B), indicating that miR-125b has antiviral activity against PRRSV replication. All the other microRNA mimics/inhibitors tested in this study had no demonstrable effect on progeny virus yield in MARC-145 cells (Figure 1A and 1B). However, we could not exclude the possibility that no anti-PRRSV effect of the miRNA mimics/inhibitors may be due to the highly/lowly expressed endogenous miRNAs.

To further exclude the possibility that the reduction effects of miR-125b on PRRSV replication resulted from cellular toxicity, MARC-145 cells were transfected with miR-125b mimic or inhibitor at different doses (30 nM, 60 nM, and 120 nM). No appreciable effect of either the mimic or inhibitor of miR-125b (at up to 120 nM) on cellular viability and morphology could be observed (data not shown). Thus, we focused our subsequent investigations on miR-125b.

**Figure 2:**
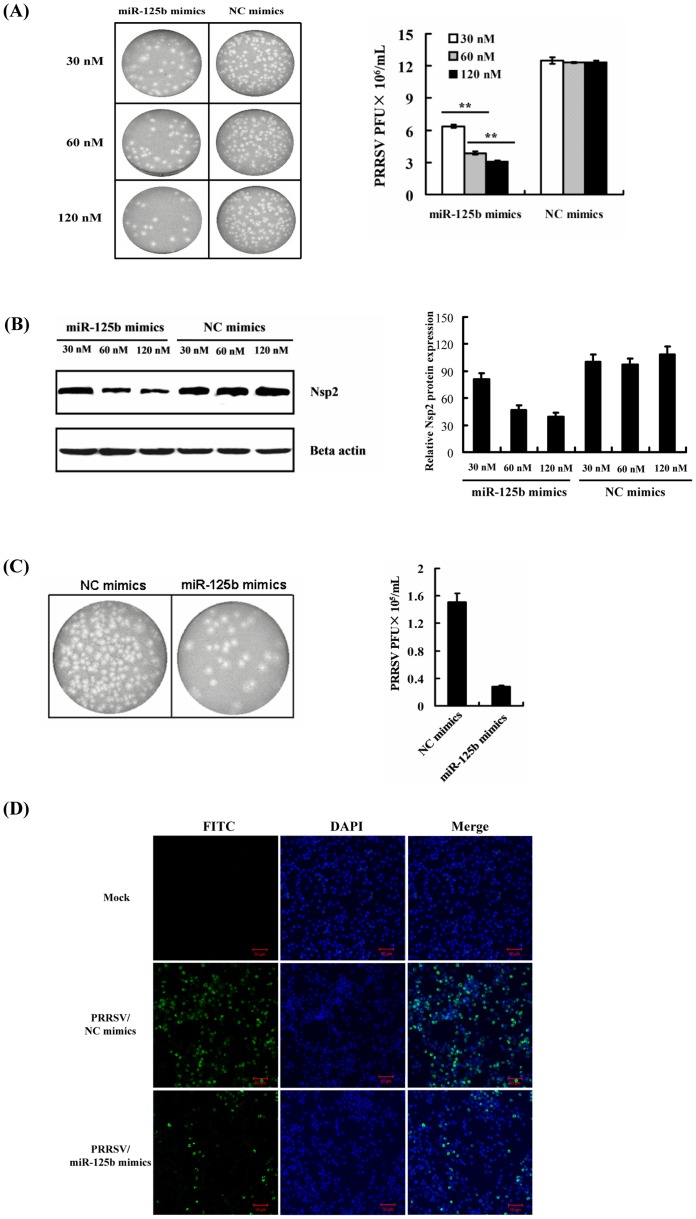
miR-125b reduces PRRSV replication in MARC-145 cells and porcine alveolar macrophages (PAMs). (A) Overexpression of miR-125b mimic reduced PRRSV replication in a dose-dependent manner. MARC-145 cells were transfected with miR-125b mimic or a control mimic (NC) at the indicated dose (30, 60, 120 nM), followed by PRRSV infection (MOI = 0.1). The infected cells were collected 48 h later for plaque assays on MARC-145 cells. The plaque results shown on the left were representative of three independent experiments (right). “**” denotes significant difference (P<0.01). (B) Overexpression of miR-125b mimic reduced the expression of PRRSV Nsp2 protein. MARC-145 cells were transfected with miR-125b mimic or the control mimic prior to PRRSV infection. Cells were collected at 30 h post-infection for western blot analysis of nsp2 expression using a specific monoclonal antibody against Nsp2 as the primary antibody. Expression of β-actin was analyzed as a loading control (left). The nsp2 expression levels were quantitated by densitometry using a Gel-Pro analyzer (Right). Data shown were representative of three independent experiments. (C) Overexpression of miR-125b mimic reduced PRRSV replication in PAMs. PAMs were transfected with miR-125b mimic or negative control (60 nM), followed by PRRSV infection (MOI = 0.1). At 48 h post-infection, cells were collected for plaque assay. Representative plaque results were shown in left panel and the data from three independent experiments were plotted in the right panel. (D) Immunofluorescence staining confirms the reduction effect of miR-125b on PRRSV replication in PAMs. The PAMs were transfected with miR-125b mimic or negative control (60 nM), followed by PRRSV infection (MOI = 0.1). Cells were fixed at 24 h post-infection and immunostained with a fluorescein isothiocyanate (FITC)-conjugated monoclonal antibody against the PRRSV N protein. Cellular nuclei were counterstained with 1 µg/mL of DAPI. Fluorescence was observed under an LSM-510 Meta confocal fluorescence microscope (Zeiss).

To corroborate our findings with miR-125b, MARC-145 cells were transfected with increasing concentrations of miR-125b mimic (30, 60, 120 nM), followed by PRRSV infection for 48 h. Virus plaque assays demonstrated that ectopic expression of miR-125b mimic, but not of a control mimic, reduced PRRSV replication in a dose-dependent manner (Figure 2A). Consistent with this, miR-125b mimic dose-dependently reduced the accumulation of PRRSV nonstructural protein 2 (nsp2), a viral replicase protein, in western blot assay (Figure 2B). Because miR-125b is highly conserved among different species, we further determined the effect of miR-125b on PRRSV replication in PAMs, the target cells of PRRSV infection *in vivo*. The PAMs transfected with miR-125b mimic (60 nM) yielded significantly lower PRRSV titers than those transfected with the control mimic (Figure 2C). Furthermore, immunofluorescence assays using a FITC-conjugated monoclonal antibody against the PRRSV N protein also supported this observation (Figure 2D). Collectively, these data unequivocally confirm that miR-125b reduces PRRSV replication.

### miR-125b does not Directly Target the PRRSV Genome

**Figure 3:**
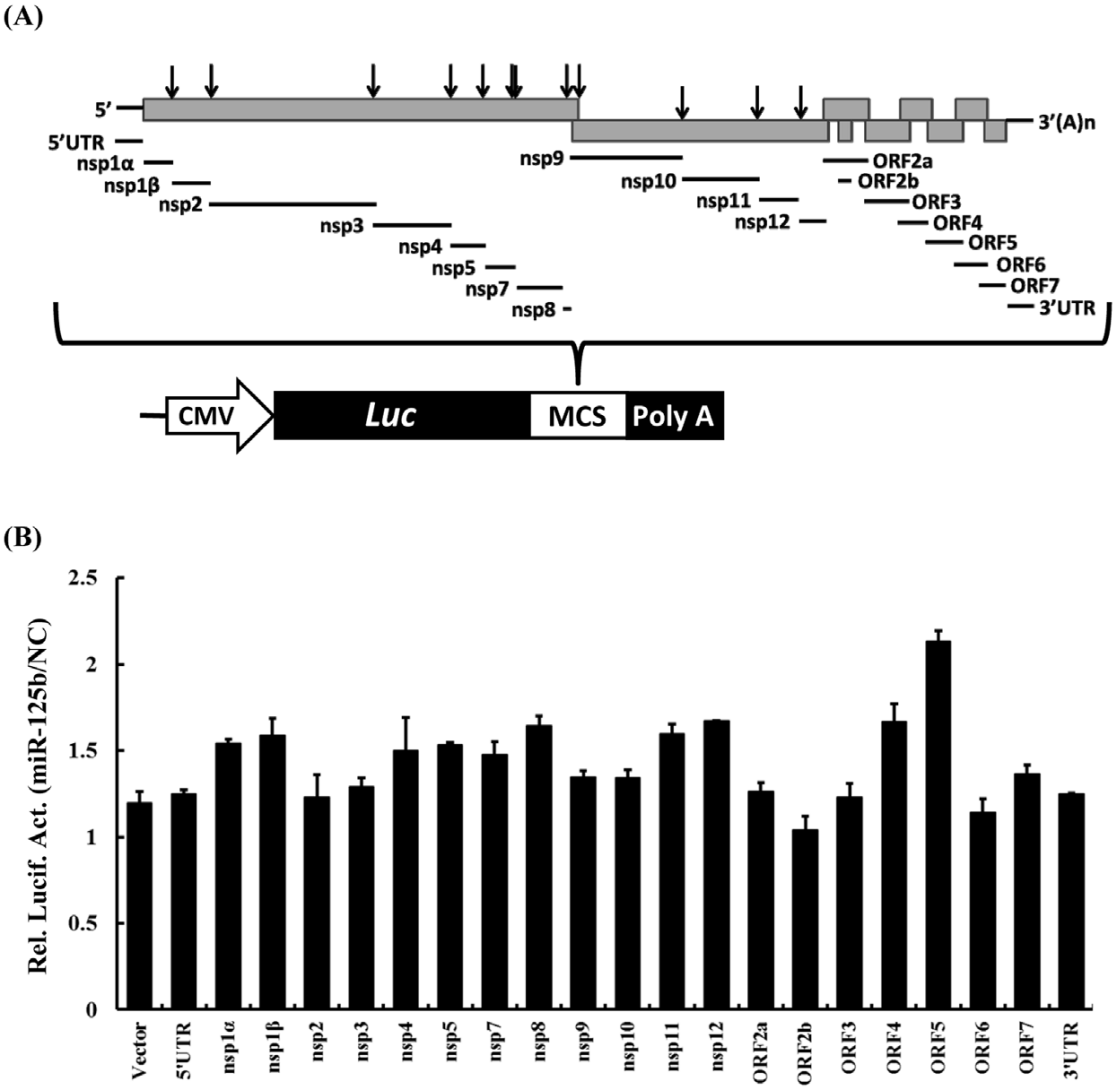
miR-125b does not directly target the PRRSV genome. (A) Schematic representation of the PRRSV genome. Viral cDNA fragments used for constructing the pMIR-REPORT-derived luciferase reporters are as indicated. (B) MARC-145 cells were co-transfected with 0.1 µg of indicated constructing luciferase reporter, 0.05 µg of pRL-TK, and 30 nM of miR-125b mimic or negative control of mimic. At 24 h post-transfection, cells were lysed for dual-luciferase assay. The relative luciferase activities (miR-125b/NC) refer to fold change in luciferase activity in miR-125b mimic-transfected cells relative to respective NC mimic-transfected controls.

Targeting a specific sequence in viral genome represents an efficient strategy of miRNAs to inhibit viral replication [22]–[25]. We determined whether miR-125b specifically targets the PRRSV genome to exert its antiviral effect. To this end, the 21 cDNA fragments representing the 5′UTR, 3′UTR and various coding regions of PRRSV genome were amplified and cloned into the reporter vector pMIR-REPORT (Ambion) downstream of the firefly luciferase gene (Figure 3A). If the PRRSV cDNA insert contains miR-125b target sequence, the expression of luciferase reporter will be subjected to regulation by miR-125b. miR-125b mimic or control mimic was co-transfected with the individual reporter vectors into MARC-145 cells, along with an internal control vector, pRL-TK (to normalize the transfection efficiency). The relative luciferase activity was determined at 24 h post-transfection. As shown in Figure 3B, the relative luciferase activities for different vectors containing various PRRSV cDNA segments were not significantly different between cells transfected with miR-125b mimic and control mimic (Figure 3B). Thus, miR-125b does not appear to directly target the PRRSV genome.

### The Anti-viral Effect of miR-125b is Independent of the Interferon (IFN) Pathway

**Figure 4:**
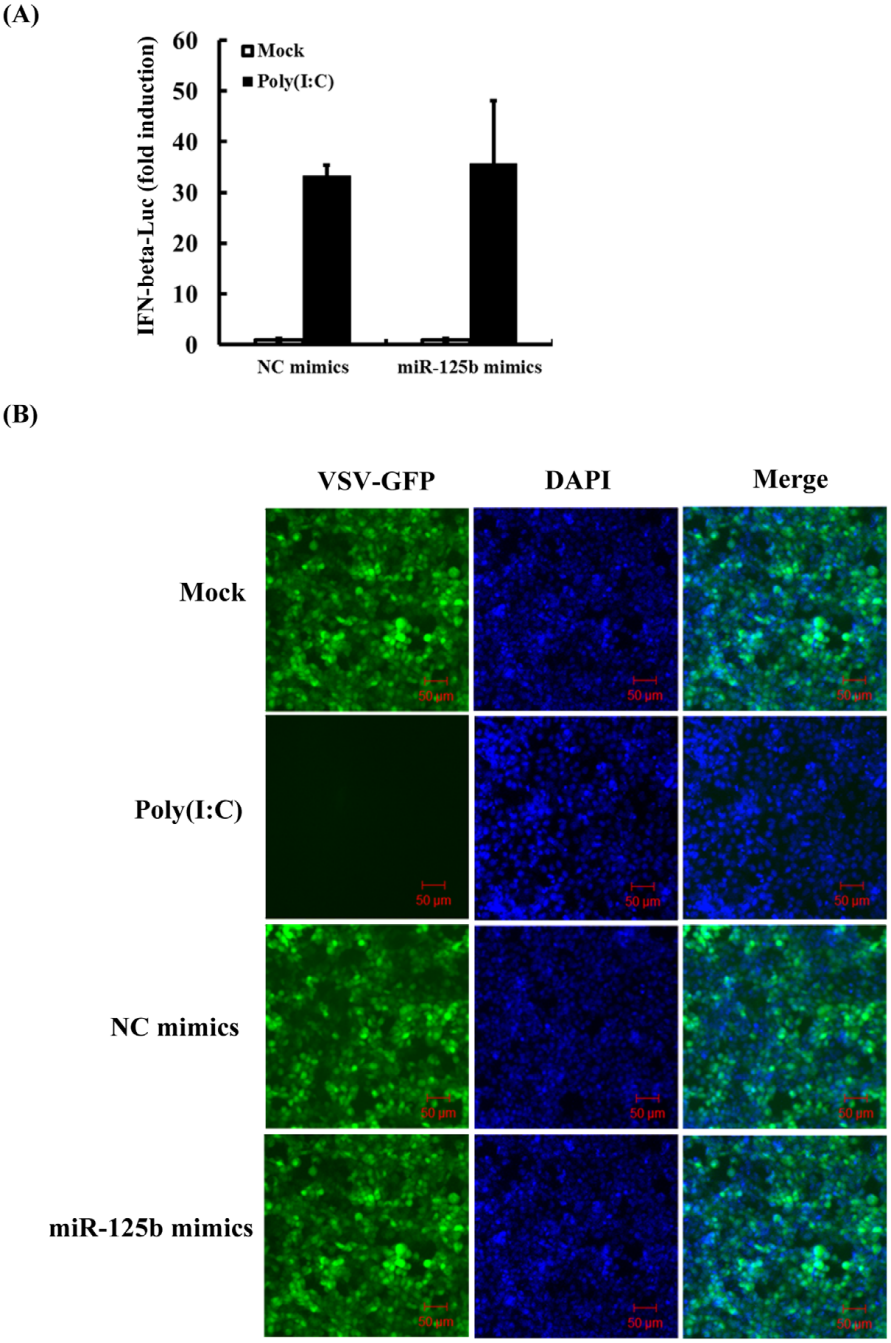
miR-125b does not induce the IFN pathway. (A) MARC-145 cells were co-transfected with IFN-β-Luc, pRL-TK, and 30 nM of miR-125b mimic or NC mimic. 24 h post-transfection, selected wells were transfected with poly(I:C) (2 µg/mL). After another 24 h, cells were lysed for dual-luciferase assay. (B) MARC-145 cells were transfected with miR-125b mimic (30 nM), NC mimic (30 nM), poly(I:C) (1 µg/mL), or mock-transfected. At 24 h post transfection, cells were infected with VSV-GFP at MOI of 0.0001. Cells were fixed at 24 h post-infection and cellular nuclei were counterstained with 1 µg/mL of DAPI. Fluorescence was observed under an LSM-510 Meta confocal fluorescence microscope (Zeiss).

Type I interferons (IFNs, mainly IFN-β and IFN-α) are known to play an important role in the antiviral innate immune response [47]. Thus, it is possible that the antiviral effect of miR-125b against PRRSV resulted from activation of the IFN response. To test this possibility, MARC-145 cells were co-transfected with the IFN-β luciferase reporter and either miR-125b mimic or NC mimic, followed by mock-stimulation or stimulation by poly(I:C). As shown in Figure 4A, when compared to the control mimic, miR-125b mimic had not demonstrable effect on the basal activity of the IFN-β promoter, nor did it affect the activation of the promoter activation by poly(I:C). Furthermore, the replication of VSV-GFP, a virus extremely sensitive to IFN’s antiviral action, was not affected in cells transfected with miR-125b mimics when compared with cells transfected with the control mimic (Figure 4B). In contrast, the replication of VSV-GFP was almost completely inhibited in cells transfected with poly(I:C), a known interferon inducer. Taken together, these data suggest that the antiviral activity of miR-125b does not involve the activation of IFN response.

### miR-125b Negatively Regulates NF-κB Activation in MARC-145 Cells

**Figure 5:**
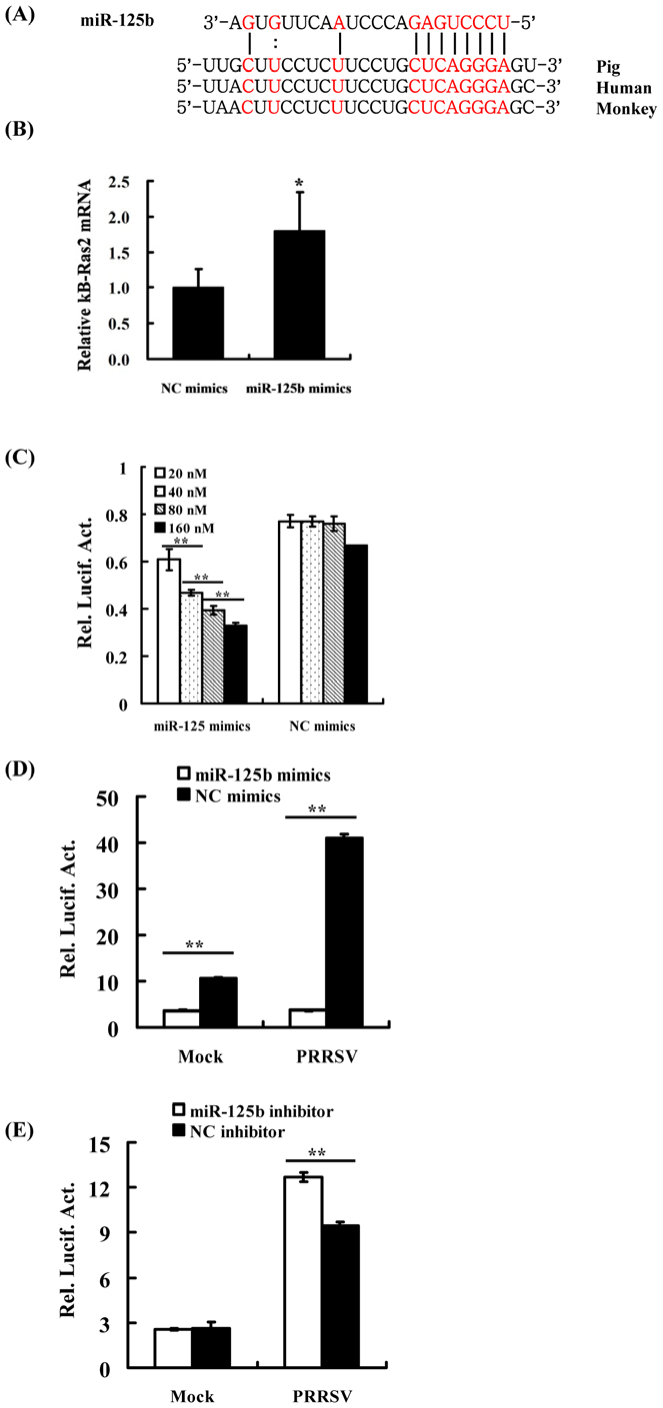
miR-125b negatively regulates NF-κB activation. (A) Alignment of miR-125b sequence with the 3′UTR of human (GenBank accession no. NM_001001349), monkey (GenBank accession no. XM_001093984) and predicted porcine (GenBank accession no. AK348039) κB-Ras2. Solid lines indicate Watson-Crick base pairs. Dotted line indicates GU wobble pairs. (B) MARC-145 cells were transfected with miR-125b mimic or NC mimic, and analyzed for κB-Ras2 mRNA expression 48 h later by qPCR. *p<0.05 *vs* NC mimic. (C) MARC-145 cells were co-transfected with pNF-κB-Luc, pRL-TK, and the indicated dose of miR-125b mimic or NC mimic. At 48 h post-transfection, cells were lysed for dual-luciferase assay. (D, E) miR-125b reduces PRRSV-induced NF-κB activation. MARC-145 cells were co-transfected with 0.1 µg of pNF-κB-Luc, 0.05 µg of pRL-TK, and 60 nM of miR-125b mimic (D) or inhibitor (E), followed by PRRSV infection 24 h later. Cells were lysed at 48 h post-infection for dual-luciferase assay. **P<0.01 as compared with NC mimic or inhibitor.

Because miR-125b does not target directly the PRRSV genome or affect cellular interferon responses, we next reasoned that miR-125b might target other proviral cellular factor(s) to reduce PRRSV replication. When we were making efforts to choose potential cellular targets of miR-125b for further study, Murphy et al. reported that miR-125b negatively regulates NF-κB by stabilizing the mRNA encoding κB-Ras2 (NF-κB inhibitor interacting Ras-like 2), a key inhibitor of NF-κB signaling [48]. If miR-125b reduces PRRSV replication by down-regulating NF-κB, activation of NF-κB should promote PRRSV replication. Previous studies by our group and others have shown that PRRSV infection activates NF-κB [49], [50]. Thus, it is plausible that PRRSV infection activates NF-κB, which, in turn, enhances PRRSV replication, and that miR-125b reduces PRRSV replication by down-regulating NF-κB activation. To test this hypothesis, we first analyzed the 3′UTR sequence of κB-Ras2 for miR-125b and found it was highly conserved between human, monkey and pig (Figure 5A). Consistent with this, ectopic expression of miR-125b mimic upregulated the abundance of κB-Ras2 transcript in MARC-145 cells (Figure 5B), presumably by stabilizing the κB-Ras2 mRNA [48]. Having confirmed that the miR-125b mimic had the desired effect on κB-Ras2 expression, we performed an NF-κB reporter assay to determine if miR-125b negatively regulated NF-κB activation in MARC-145 cells. As shown in Figure 5C, the ecotopically expressed miR-125b mimic down-regulated the basal NF-κB activity in a dose-dependent manner in MARC-145 cells, which was in agreement with the reported effect of miR-125b in human macrophages [48]. We next investigated whether miR-125b affects PRRSV-induced NF-κB activation. To this end, MARC-145 cells were co-transfected with the NF-κB luciferase reporter and either miR-125b mimic or NC mimic, followed by PRRSV infection, to detect the NF-κB promoter activity. As previously reported [49], [50], we found PRRSV infection substantially stimulated NF-κB activity in cells transfected with the NC mimic. Importantly, ectopic expression of the miR-125b mimic not only reduced the basal NF-κB activity but also ablated that activated by PRRSV (Figure 5D). Conversely, transfection of the miR-125b inhibitor significantly augmented PRRSV-induced NF-κB (Figure 5E). Collectively, these data demonstrate that miR-125b negatively regulates cellular basal NF-κB activity as well as that induced by PRRSV infection.

### The Inter-relationship among miR-125b, NF-κB Activation and PRRSV Replication

**Figure 6:**
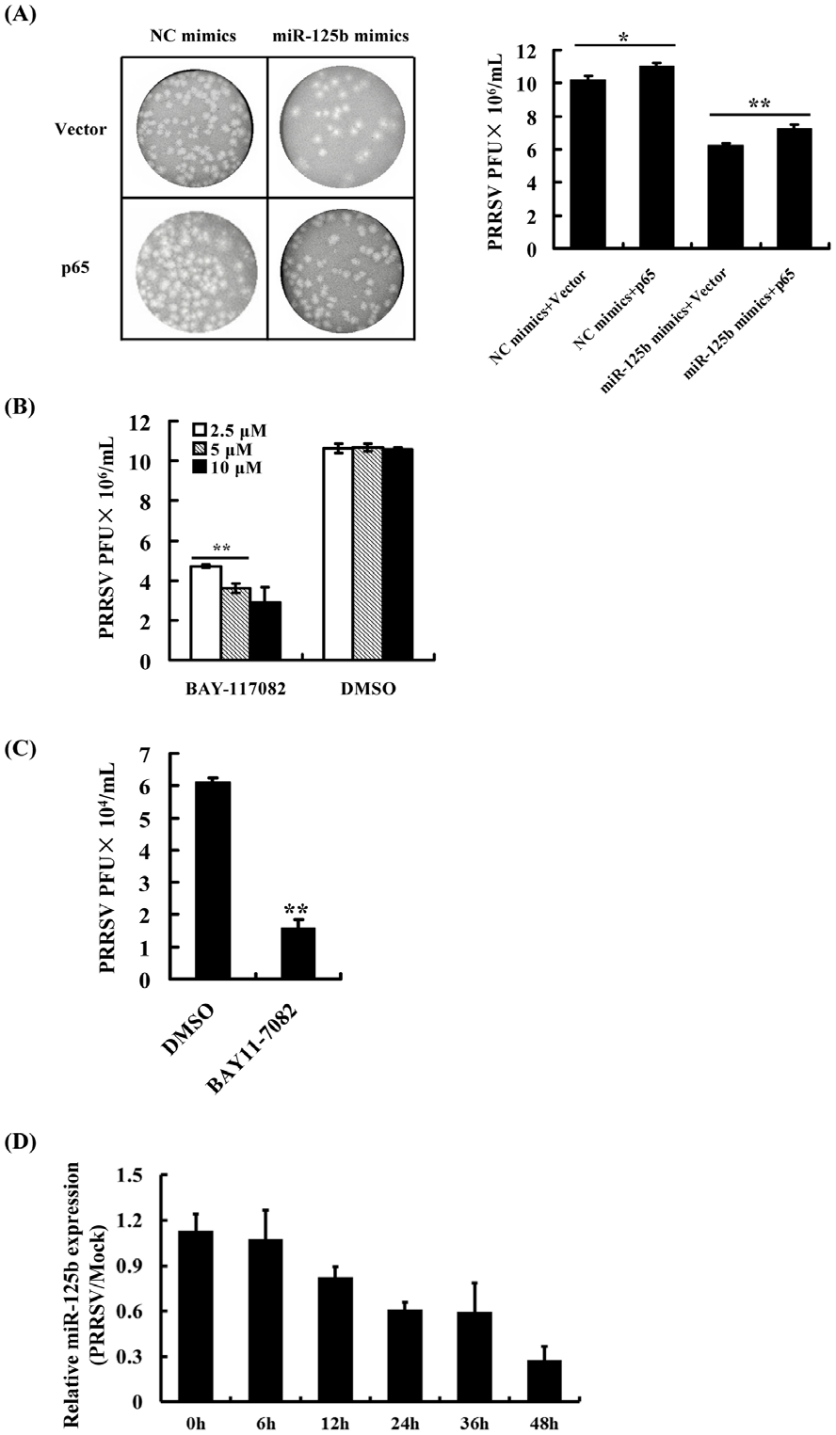
The inter-relationship among miR-125b, NF-κB activation and PRRSV replication. (A) Overexpression of the NF-κB p65 subunit promotes PRRSV replication and partially antagonizes miR-125b’s effect on PRRSV. MARC-145 cells were cotransfected with a control vector or vector encoding p65 (1.0 µg) and 60 nM of miR-125b mimic or inhibitor. The transfected cells were infected with PRRSV WUH3 strain (MOI = 0.01) 24 h later. Cells were collected at 48 h post-infection for plaque assay on MARC-145 cells. Virus titers were expressed as PFU/mL. Representative plaque results from three independent experiments are shown in left panel and the average results are illustrated on the right. **P<0.01 and *P<0.05 as compared with cells transfected with the control vector. (B, C) Pretreatment with the NF-κB inhibitor BAY11-7082 reduces PRRSV replication in MARC-145 cells (2.5 µM, 5.0 µM and 10µM of BAY11-7082, panel B) and PAMs (5 µM, panel C). Cells were pretreated with BAY11-7082 for 1 h prior to PRRSV infection. At 48 h post-infection, cells were collected and virus titers were determined by plaque assay on MARC-145 cells. (D) The time-course expression of miR-125b after PRRSV infection. MARC-145 cells infected with PRRSV at a MOI of 0.1 were collected at the indicated time points and qRT-PCR analysis was performed to detect miR-125b expression. The miR-125b expression level at 6 h in mock-infected cells was used as the baseline (1.0) for comparison.

It was previously shown that the activation of NF-κB by PRRSV infection involves IκB degradation and nuclear translocation of p65, a key subunit of NF-κB [49]. To further investigate the inter-relations among miR-125b, NF-κB and PRRSV replication, the DNA construct encoding p65 was co-transfected with miR-125b mimic into MARC-145 cells prior to PRRSV infection. Viral plaque assays showed that coexpression of p65 partially reversed the reduction effect of miR-125b on PRRSV replication (Figure 6A). Of note, in the absence of miR-125b mimic, overexpression of p65 also increased PRRSV replication compared to cells transfected with the control vector. Additionally, we examined whether NF-κB was required for optimal PRRSV replication. MARC-145 cells pretreated with BAY11-7082, a specific NF-κB inhibitor, for 1 h prior to PRRSV infection yielded significantly lower progeny PRRSV titers than those pretreated with DMSO (Figure 6B). Similar results were obtained in PRRSV-infected PAMs (Figure 6C).

Based on the results above and those from previous studies [49], [50], it appears that PRRSV infection induces NF-κB activation, which facilitates PRRSV replication, while miR-125b negatively regulates NF-κB signaling, thereby reducing PRRSV replication. If that is really the case, PRRSV infection should down-regulate miR-125b expression for its survival advantage. Therefore, we analyzed the temporal kinetics of miR-125b expression in PRRSV infected MARC-145 cells by qPCR (primer sequences shown in Table S2). As predicted, significantly decreased miR-125b expression was first observed at 12 h post PRRSV infection, and the miR-125b abundance was further reduced gradually as the infection progressed. At 48 h post-infection, the miR-125b abundance had decreased to nearly 30% of the pre-infection level (Figure 6D). In aggregate, these data support the notion that PRRSV infection down-regulates miR-125b to negate the latter’s inhibitory effect on NF-κB, thereby facilitating viral replication.

## Discussion

There is a growing body of evidence that cellular miRNAs serve as critical effectors in intricate networks of host–pathogen interactions [8]–[12]. Herein, we provide data demonstrating that miR-125b is a novel antiviral host factor against PRRSV, an economically important animal virus that devastates the swine industry worldwide. Ectopically expressed miR-125b reduced PRRSV progeny virus production. Conversely, inhibition of miR-125b substantially enhanced PRRSV propagation (Figure 1). Importantly, we observed this not only in MARC-145 cells but also in PAMs (Figure 2), the main target cell type for PRRSV replication *in vivo*, confirming the biological relevance of this finding. Because targeting host factors for developing antiviral drugs has the advantage of higher genetic barrier to the emergence of viral escape mutants, the identification and characterization of miR-125 as an inhibitor of PRRSV replication may open new ways of controlling future PRRS outbreaks, for which effective control measures remain scanty.

**Figure 7:**
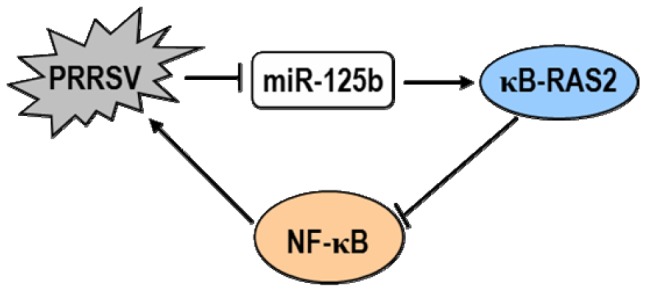
The proposed model for the inter-relationship among miR-125b, NF-κB activation and PRRSV replication. PRRSV infection down-regulates the expression of miR-125b, which relieves the stabilizing effect on κB-Ras2 mRNA, resulting in subsequent NF-κB activation. The activated NF-κB promotes PRRSV replication.

To date, the mechanisms by which cellular miRNAs regulate viral replication have been shown to fall into two categories. The first one is to target a sequence in the genome of virus [17]–[25]. This strategy is very efficient because it is direct and sequence specific. The second mechanism involves the regulation of cellular pathways perturbing the viral life cycle. In particular, the activation or enhancement of innate antiviral immune pathways has been suggested to be responsible for the antiviral effect of certain miRNAs [26]. This mechanism is indirect and complex and usually involves the participation of many cellular signaling molecules. In the current study, the reduction of PRRSV replication by miR-125b did not appear to involve direct targeting of the PRRSV genome (Figure 3), nor did it result from activation/augmentation of the IFN response (Figure 4). These data led us to reason that miR-125b might act on a proviral cellular factor(s)/pathway(s) to reduce PRRSV replication. Considering that miR-125b negatively regulates NF-κB by stabilizing the mRNA encoding κB-Ras2 in human cells and that PRRSV infection activates NF-κB, we studied the relationship of miR-125b expression, NF-κB activation, and PRRSV replication. We found that overexpression of miR-125b stabilized κB-Ras2 mRNA and down-regulated of NF-κB activation in MARC-145 cells (Figure 5), and that pharmacological inhibition of NF-κB impaired PRRSV replication (Figure 6). Therefore, we propose a working model in which miR-125b negatively regulates NF-κB, thereby reducing PRRSV replication (Figure7). However, it is important to recognize the possibility that miR-125b may also target other unrecognized cellular factor(s)/pathway(s) pivotal for PRRSV replication as part of it antiviral mechanisms. Of note, in addition to acting on κB-Ras2, miR-125b is also predicted (by TargetScan, miRanda and PicTar) to target a number of cellular mRNAs encoding proteins involved in apoptosis and inflammation processes, such as NAIF1 (nuclear apoptosis inducing factor 1), BAK1 (BCL2-antagonist/killer 1), RIT1 (Ras-like without CAAX 1), KSR2 (kinase suppressor of Ras 2), BCL2L12 (BCL2-like 12), and IRF4 (IFN regulatory factor 4), several of which have been validated as miR-125b targets recently [48], [51]–[56]. Whether any of these potential targets are involved in the antiviral program of miR-125b against PRRSV will require future investigation.

Although NF-κB has long been considered a key transcription factor for the expression of a variety of antiviral cytokines [57], some pathogens redirect the activity of NF-κB into a virus-supportive function [58], [59]. For example, influenza viruses replicated to higher titers in cells with pre-activated NF-κB, and conversely, progeny virus production was reduced when NF-κB signaling was impaired [60], [61]. Williams et al [62] reported that sustained induction of NF-κB is required for efficient expression of latent HIV type 1. We have shown in this study that optimal replication of PRRSV, an Arterivirus, also relies on NF-κB. However, the exact role of NF-κB in the life cycle of PRRSV is elusive. Data on the interplay between PRRSV and the NF-κB pathway have also been somewhat controversial. Lee et al. [49] firstly demonstrated that PRRSV infection activated NF-κB signaling in MARC-145 cells and PAMs that involved IκB degradation and p65 nuclear translocation. Our group also showed that PRRSV infection triggered NF-κB activation [50] and the nucleocapsid (N) protein of PRRSV could activate NF-κB when ectopically expressed in MARC-145 cells [63]. However, the activated NF-κB could only be detected after 24 h postinfection. In contrast, the ectopic expression of several PRRSV nsps as an individual protein, such as nsp1α, nsp1β, nsp2, and nsp11, was reported to negatively regulate NF-κB activation. For example, Sun et al [40] reported that PRRSV nsp2 inhibited the NF-κB signaling pathway by interfering with the polyubiquitination process of IκBα. Song et al [64] documented that PRRSV nsp1α could inhibit NF-κB activation and suppress IFN-β production, but they also found that NF-κB activity was increased at 1 and 2 days post-PRRSV infection in MARC-145 cells using an NF-κB-luciferase reporter assay, which is consistent with the findings of Lee et al [49] and our group [50]. Taken collectively, it is reasonable to conclude that PRRSV activates NF-κB at late phases of infection, and that PRRSV may have developed sophisticated strategies to either activate or inhibit NF-κB for its own benefit in different stages of its life cycle. The elaborate mechanisms by which PRRSV regulates NF-κB activation and how the latter promotes PRRSV replication warrant further studies.

It has been reported that miR-125b is highly expressed and enriched in macrophages [51]. We compared the basal expression levels of miR-125b, miR-155, miR-23a and miR-365 in PAMs by qPCR and found that miR-125b was among the most highly expressed miRNAs examined (data not shown). Interestingly, we found that PRRSV infection down-regulated the expression of miR-125b as the infection progressed. Significant down-regulation was first observed at 12 h, and further reductions in miR-125b abundance took place at later time points (Figure 6D). It is plausible to speculate that PRRSV infection gradually decreases miR-125b mRNA expression, which, in turn, relieves the stabilizing effect on κB-Ras2 mRNA, ultimately leading to subsequent NF-κB activation. In addition, our group showed that toll-like receptors (TLRs) signaling cascade may be also involved in PRRSV-induced NF-κB activation [65].

In summary, our data demonstrate that miR-125b is an antiviral host factor that restricts PRRSV replication. Instead of directly targeting the PRRSV genome, miR-125b exerts it antiviral effect by negatively regulating cellular NF-κB signaling, which we have shown to be a proviral factor for PRRSV replication (Figure 7). As a survival strategy, PRRSV downregulates the expression of miR-125b post-infection and activates NF-κB to facilitate its own multiplication. Our study reveals an example of manipulation of a cellular miRNA by an arterivirus to re-orchestrate host gene expression for viral propagation and sheds new light on targeting host factors to develop effective control measures for PRRS.

## Supporting Information

Table S1 **The sequences of microRNA (miRNA) mimics and inhibitors used in this study.**
(DOC)

Table S2 **Sequence of oligonucleotide primers used in this study.**
(DOC)

## References

[1] Ambros V (2004) The functions of animal microRNAs. Nature 431: 350–355. 15372042 10.1038/nature02871

[2] Lee Y, Jeon K, Lee JT, Kim S, Kim VN (2002) MicroRNA maturation: stepwise processing and subcellular localization. Embo J 21: 4663–4670. 12198168 10.1093/emboj/cdf476 PMC126204

[3] Yi R, Qin Y, Macara IG, Cullen BR (2003) Exportin-5 mediates the nuclear export of pre-microRNAs and short hairpin RNAs. Genes Dev 17: 3011–3016. 14681208 10.1101/gad.1158803 PMC305252

[4] Lee Y, Ahn C, Han J, Choi H, Kim J, et al (2003) The nuclear RNase III Drosha initiates microRNA processing. Nature 425: 415–419. 14508493 10.1038/nature01957

[5] Bartel DP (2004) MicroRNAs: genomics, biogenesis, mechanism, and function. Cell 116: 281–297. 14744438 10.1016/s0092-8674(04)00045-5

[6] Kiriakidou M, Nelson PT, Kouranov A, Fitziev P, Bouyioukos C, et al (2004) A combined computational-experimental approach predicts human microRNA targets. Genes Dev 18: 1165–1178. 15131085 10.1101/gad.1184704 PMC415641

[7] Lewis BP, Shih IH, Jones-Rhoades MW, Bartel DP, Burge CB (2003) Prediction of mammalian microRNA targets. Cell 115: 787–798. 14697198 10.1016/s0092-8674(03)01018-3

[8] Boss IW, Renne R (2010) Viral miRNAs: tools for immune evasion. Curr Opin Microbiol 13: 540–545. 20580307 10.1016/j.mib.2010.05.017 PMC2920354

[9] Gottwein E, Cullen BR (2008) Viral and cellular microRNAs as determinants of viral pathogenesis and immunity. Cell Host Microbe 3: 375–387. 18541214 10.1016/j.chom.2008.05.002 PMC3079432

[10] Muller S, Imler JL (2007) Dicing with viruses: microRNAs as antiviral factors. Immunity 27: 1–3. 17663977 10.1016/j.immuni.2007.07.003

[11] Roberts AP, Jopling CL (2010) Targeting viral infection by microRNA inhibition. Genome Biol 11: 201. 20122293 10.1186/gb-2010-11-1-201 PMC2847709

[12] Sarnow P, Jopling CL, Norman KL, Schutz S, Wehner KA (2006) MicroRNAs: expression, avoidance and subversion by vertebrate viruses. Nat Rev Microbiol 4: 651–659. 16912711 10.1038/nrmicro1473

[13] Chang J, Guo JT, Jiang D, Guo H, Taylor JM, et al (2008) Liver-specific microRNA miR-122 enhances the replication of hepatitis C virus in nonhepatic cells. J Virol 82: 8215–8223. 18550664 10.1128/JVI.02575-07 PMC2519557

[14] Norman KL, Sarnow P (2010) Modulation of hepatitis C virus RNA abundance and the isoprenoid biosynthesis pathway by microRNA miR-122 involves distinct mechanisms. J Virol 84: 666–670. 19846523 10.1128/JVI.01156-09 PMC2798415

[15] Skalsky RL, Cullen BR (2010) Viruses, microRNAs, and host interactions. Annu Rev Microbiol 64: 123–141. 20477536 10.1146/annurev.micro.112408.134243 PMC3621958

[16] Wang FZ, Weber F, Croce C, Liu CG, Liao X, et al (2008) Human cytomegalovirus infection alters the expression of cellular microRNA species that affect its replication. J Virol 82: 9065–9074. 18596100 10.1128/JVI.00961-08 PMC2546905

[17] Jangra RK, Yi M, Lemon SM (2010) Regulation of hepatitis C virus translation and infectious virus production by the microRNA miR-122. J Virol 84: 6615–6625. 20427538 10.1128/JVI.00417-10 PMC2903297

[18] Jopling CL, Yi M, Lancaster AM, Lemon SM, Sarnow P (2005) Modulation of hepatitis C virus RNA abundance by a liver-specific MicroRNA. Science 309: 1577–1581. 16141076 10.1126/science.1113329

[19] Murakami Y, Aly HH, Tajima A, Inoue I, Shimotohno K (2009) Regulation of the hepatitis C virus genome replication by miR-199a. J Hepatol 50: 453–460. 19144437 10.1016/j.jhep.2008.06.010

[20] Pedersen IM, Cheng G, Wieland S, Volinia S, Croce CM, et al (2007) Interferon modulation of cellular microRNAs as an antiviral mechanism. Nature 449: 919–922. 17943132 10.1038/nature06205 PMC2748825

[21] Lecellier CH, Dunoyer P, Arar K, Lehmann-Che J, Eyquem S, et al (2005) A cellular microRNA mediates antiviral defense in human cells. Science 308: 557–560. 15845854 10.1126/science.1108784

[22] Song L, Liu H, Gao S, Jiang W, Huang W (2010) Cellular microRNAs inhibit replication of the H1N1 influenza A virus in infected cells. J Virol 84: 8849–8860. 20554777 10.1128/JVI.00456-10 PMC2919005

[23] Huang J, Wang F, Argyris E, Chen K, Liang Z, et al (2007) Cellular microRNAs contribute to HIV-1 latency in resting primary CD4+ T lymphocytes. Nat Med 13: 1241–1247. 17906637 10.1038/nm1639

[24] Zhang GL, Li YX, Zheng SQ, Liu M, Li X, et al (2010) Suppression of hepatitis B virus replication by microRNA-199a-3p and microRNA-210. Antiviral Res 88: 169–175. 20728471 10.1016/j.antiviral.2010.08.008

[25] Otsuka M, Jing Q, Georgel P, New L, Chen J, et al (2007) Hypersusceptibility to vesicular stomatitis virus infection in Dicer1-deficient mice is due to impaired miR24 and miR93 expression. Immunity 27: 123–134. 17613256 10.1016/j.immuni.2007.05.014

[26] Wang P, Hou J, Lin L, Wang C, Liu X, et al (2010) Inducible microRNA-155 feedback promotes type I IFN signaling in antiviral innate immunity by targeting suppressor of cytokine signaling 1. J Immunol 185: 6226–6233. 20937844 10.4049/jimmunol.1000491

[27] Rossow KD (1998) Porcine reproductive and respiratory syndrome. Vet Pathol 35: 1–20. 9545131 10.1177/030098589803500101

[28] Meulenberg JJ (2000) PRRSV, the virus. Vet Res 31: 11–21. 10726635 10.1051/vetres:2000103

[29] Murtaugh MP, Stadejek T, Abrahante JE, Lam TT, Leung FC (2010) The ever-expanding diversity of porcine reproductive and respiratory syndrome virus. Virus Res 154: 18–30. 20801173 10.1016/j.virusres.2010.08.015

[30] Darwich L, Diaz I, Mateu E (2010) Certainties, doubts and hypotheses in porcine reproductive and respiratory syndrome virus immunobiology. Virus Res 154: 123–132. 20659507 10.1016/j.virusres.2010.07.017

[31] Meng XJ (2000) Heterogeneity of porcine reproductive and respiratory syndrome virus: implications for current vaccine efficacy and future vaccine development. Vet Microbiol 74: 309–329. 10831854 10.1016/S0378-1135(00)00196-6 PMC7117501

[32] Calzada-Nova G, Schnitzlein WM, Husmann RJ, Zuckermann FA (2011) North American porcine reproductive and respiratory syndrome viruses inhibit type I interferon production by plasmacytoid dendritic cells. J Virol 85: 2703–2713. 21191013 10.1128/JVI.01616-10 PMC3067927

[33] Kimman TG, Cornelissen LA, Moormann RJ, Rebel JM, Stockhofe-Zurwieden N (2009) Challenges for porcine reproductive and respiratory syndrome virus (PRRSV) vaccinology. Vaccine 27: 3704–3718. 19464553 10.1016/j.vaccine.2009.04.022

[34] Yoo D, Song C, Sun Y, Du Y, Kim O, et al (2010) Modulation of host cell responses and evasion strategies for porcine reproductive and respiratory syndrome virus. Virus Res 154: 48–60. 20655963 10.1016/j.virusres.2010.07.019 PMC7114477

[35] Beura LK, Sarkar SN, Kwon B, Subramaniam S, Jones C, et al (2010) Porcine reproductive and respiratory syndrome virus nonstructural protein 1beta modulates host innate immune response by antagonizing IRF3 activation. J Virol 84: 1574–1584. 19923190 10.1128/JVI.01326-09 PMC2812326

[36] Chen Z, Zhou X, Lunney JK, Lawson S, Sun Z, et al (2010) Immunodominant epitopes in nsp2 of porcine reproductive and respiratory syndrome virus are dispensable for replication, but play an important role in modulation of the host immune response. J Gen Virol 91: 1047–1057. 19923257 10.1099/vir.0.016212-0

[37] Kim O, Sun Y, Lai FW, Song C, Yoo D (2010) Modulation of type I interferon induction by porcine reproductive and respiratory syndrome virus and degradation of CREB-binding protein by non-structural protein 1 in MARC-145 and HeLa cells. Virology 402: 315–326. 20416917 10.1016/j.virol.2010.03.039 PMC7157927

[38] Murtaugh MP, Xiao Z, Zuckermann F (2002) Immunological responses of swine to porcine reproductive and respiratory syndrome virus infection. Viral Immunol 15: 533–547. 12513925 10.1089/088282402320914485

[39] Patel D, Nan Y, Shen M, Ritthipichai K, Zhu X, et al (2010) Porcine reproductive and respiratory syndrome virus inhibits type I interferon signaling by blocking STAT1/STAT2 nuclear translocation. J Virol 84: 11045–11055. 20739522 10.1128/JVI.00655-10 PMC2953160

[40] Sun Z, Chen Z, Lawson SR, Fang Y (2010) The cysteine protease domain of porcine reproductive and respiratory syndrome virus nonstructural protein 2 possesses deubiquitinating and interferon antagonism functions. J Virol 84: 7832–7846. 20504922 10.1128/JVI.00217-10 PMC2897636

[41] Wensvoort G, Terpstra C, Pol JM, ter Laak EA, Bloemraad M, et al (1991) Mystery swine disease in The Netherlands: the isolation of Lelystad virus. Vet Q 13: 121–130. 1835211 10.1080/01652176.1991.9694296

[42] Li B, Xiao S, Wang Y, Xu S, Jiang Y, et al (2009) Immunogenicity of the highly pathogenic porcine reproductive and respiratory syndrome virus GP5 protein encoded by a synthetic ORF5 gene. Vaccine 27: 1957–1963. 19368777 10.1016/j.vaccine.2009.01.098

[43] Wang D, Fang L, Luo R, Ye R, Fang Y, et al (2010) Foot-and-mouth disease virus leader proteinase inhibits dsRNA-induced type I interferon transcription by decreasing interferon regulatory factor 3/7 in protein levels. Biochem Biophys Res Commun 399: 72–78. 20638368 10.1016/j.bbrc.2010.07.044

[44] Luo R, Fang L, Jin H, Jiang Y, Wang D, et al (2011) Antiviral activity of type I and type III interferons against porcine reproductive and respiratory syndrome virus (PRRSV). Antiviral Res 91: 99–101. 21569798 10.1016/j.antiviral.2011.04.017

[45] Asirvatham AJ, Gregorie CJ, Hu Z, Magner WJ, Tomasi TB (2008) MicroRNA targets in immune genes and the Dicer/Argonaute and ARE machinery components. Mol Immunol 45: 1995–2006. 18061676 10.1016/j.molimm.2007.10.035 PMC2678893

[46] Wessner B, Gryadunov-Masutti L, Tschan H, Bachl N, Roth E (2010) Is there a role for microRNAs in exercise immunology? A synopsis of current literature and future developments. Exerc Immunol Rev 16: 22–39. 20839489

[47] Kawai T, Akira S (2006) Innate immune recognition of viral infection. Nat Immunol 7: 131–137. 16424890 10.1038/ni1303

[48] Murphy AJ, Guyre PM, Pioli PA (2010) Estradiol suppresses NF-kappa B activation through coordinated regulation of let-7a and miR-125b in primary human macrophages. J Immunol 184: 5029–5037. 20351193 10.4049/jimmunol.0903463 PMC2882792

[49] Lee SM, Kleiboeker SB (2005) Porcine arterivirus activates the NF-kappaB pathway through IkappaB degradation. Virology 342: 47–59. 16129468 10.1016/j.virol.2005.07.034 PMC7111765

[50] Luo R, Xiao S, Jiang Y, Jin H, Wang D, et al (2008) Porcine reproductive and respiratory syndrome virus (PRRSV) suppresses interferon-beta production by interfering with the RIG-I signaling pathway. Mol Immunol 45: 2839–2846. 18336912 10.1016/j.molimm.2008.01.028 PMC7112510

[51] Chaudhuri AA, So AY, Sinha N, Gibson WS, Taganov KD, et al (2011) MicroRNA-125b Potentiates Macrophage Activation. J Immunol 187: 5062–5068. 22003200 10.4049/jimmunol.1102001 PMC3208133

[52] Malumbres R, Sarosiek KA, Cubedo E, Ruiz JW, Jiang X, et al (2009) Differentiation stage-specific expression of microRNAs in B lymphocytes and diffuse large B-cell lymphomas. Blood 113: 3754–3764. 19047678 10.1182/blood-2008-10-184077 PMC2670792

[53] Kong F, Sun C, Wang Z, Han L, Weng D, et al (2011) miR-125b confers resistance of ovarian cancer cells to cisplatin by targeting pro-apoptotic Bcl-2 antagonist killer 1. J Huazhong Univ Sci Technolog Med Sci 31: 543–549. 21823019 10.1007/s11596-011-0487-z

[54] Surdziel E, Cabanski M, Dallmann I, Lyszkiewicz M, Krueger A, et al (2011) Enforced expression of miR-125b affects myelopoiesis by targeting multiple signaling pathways. Blood 117: 4338–4348. 21368288 10.1182/blood-2010-06-289058

[55] Shi XB, Xue L, Ma AH, Tepper CG, Kung HJ, et al (2011) miR-125b promotes growth of prostate cancer xenograft tumor through targeting pro-apoptotic genes. Prostate 71: 538–549. 20886540 10.1002/pros.21270 PMC3017658

[56] Shi XB, Xue L, Yang J, Ma AH, Zhao J, et al (2007) An androgen-regulated miRNA suppresses Bak1 expression and induces androgen-independent growth of prostate cancer cells. Proc Natl Acad Sci U S A 104: 19983–19988. 18056640 10.1073/pnas.0706641104 PMC2148409

[57] Hayden MS, Ghosh S (2008) Shared principles in NF-kappaB signaling. Cell 132: 344–362. 18267068 10.1016/j.cell.2008.01.020

[58] Asamitsu K, Yamaguchi T, Nakata K, Hibi Y, Victoriano AF, et al (2008) Inhibition of human immunodeficiency virus type 1 replication by blocking IkappaB kinase with noraristeromycin. J Biochem 144: 581–589. 18713798 10.1093/jb/mvn104

[59] Ludwig S, Planz O (2008) Influenza viruses and the NF-kappaB signaling pathway - towards a novel concept of antiviral therapy. Biol Chem 389: 1307–1312. 18713017 10.1515/BC.2008.148

[60] Nimmerjahn F, Dudziak D, Dirmeier U, Hobom G, Riedel A, et al (2004) Active NF-kappaB signalling is a prerequisite for influenza virus infection. J Gen Virol 85: 2347–2356. 15269376 10.1099/vir.0.79958-0

[61] Wurzer WJ, Ehrhardt C, Pleschka S, Berberich-Siebelt F, Wolff T, et al (2004) NF-kappaB-dependent induction of tumor necrosis factor-related apoptosis-inducing ligand (TRAIL) and Fas/FasL is crucial for efficient influenza virus propagation. J Biol Chem 279: 30931–30937. 15143063 10.1074/jbc.M403258200

[62] Williams SA, Kwon H, Chen LF, Greene WC (2007) Sustained induction of NF-kappa B is required for efficient expression of latent human immunodeficiency virus type 1. J Virol 81: 6043–6056. 17376917 10.1128/JVI.02074-06 PMC1900291

[63] Luo R, Fang L, Jiang Y, Jin H, Wang Y, et al (2010) Activation of NF-kappaB by nucleocapsid protein of the porcine reproductive and respiratory syndrome virus. Virus Genes 42: 76–81. 21063763 10.1007/s11262-010-0548-6

[64] Song C, Krell P, Yoo D (2010) Nonstructural protein 1alpha subunit-based inhibition of NF-kappaB activation and suppression of interferon-beta production by porcine reproductive and respiratory syndrome virus. Virology 407: 268–280. 20850164 10.1016/j.virol.2010.08.025

[65] Song S, Bi J, Wang D, Fang L, Zhang L, et al.. (2012) Porcine reproductive and respiratory syndrome virus infection activates IL-10 production through NF-κB and p38 MAPK pathways in porcine alveolar macrophages. Dev Comp Immunol doi: 10.1016/j.dci.2012.10.001. 10.1016/j.dci.2012.10.001 23085400

