# Correction: MiR-125b Reduces Porcine Reproductive and Respiratory Syndrome Virus Replication by Negatively Regulating the NF-κB Pathway

**DOI:** 10.1371/journal.pone.0314312

**Published:** 2024-11-18

**Authors:** Dang Wang, Lu Cao, Zheng Xu, Liurong Fang, Yao Zhong, Quangang Chen, Rui Luo, Huanchun Chen, Kui Li, Shaobo Xiao

There are errors in Fig 4B and Fig 6A. Please see the correct Fig 4B and Fig 6A here.

**Fig 4:**
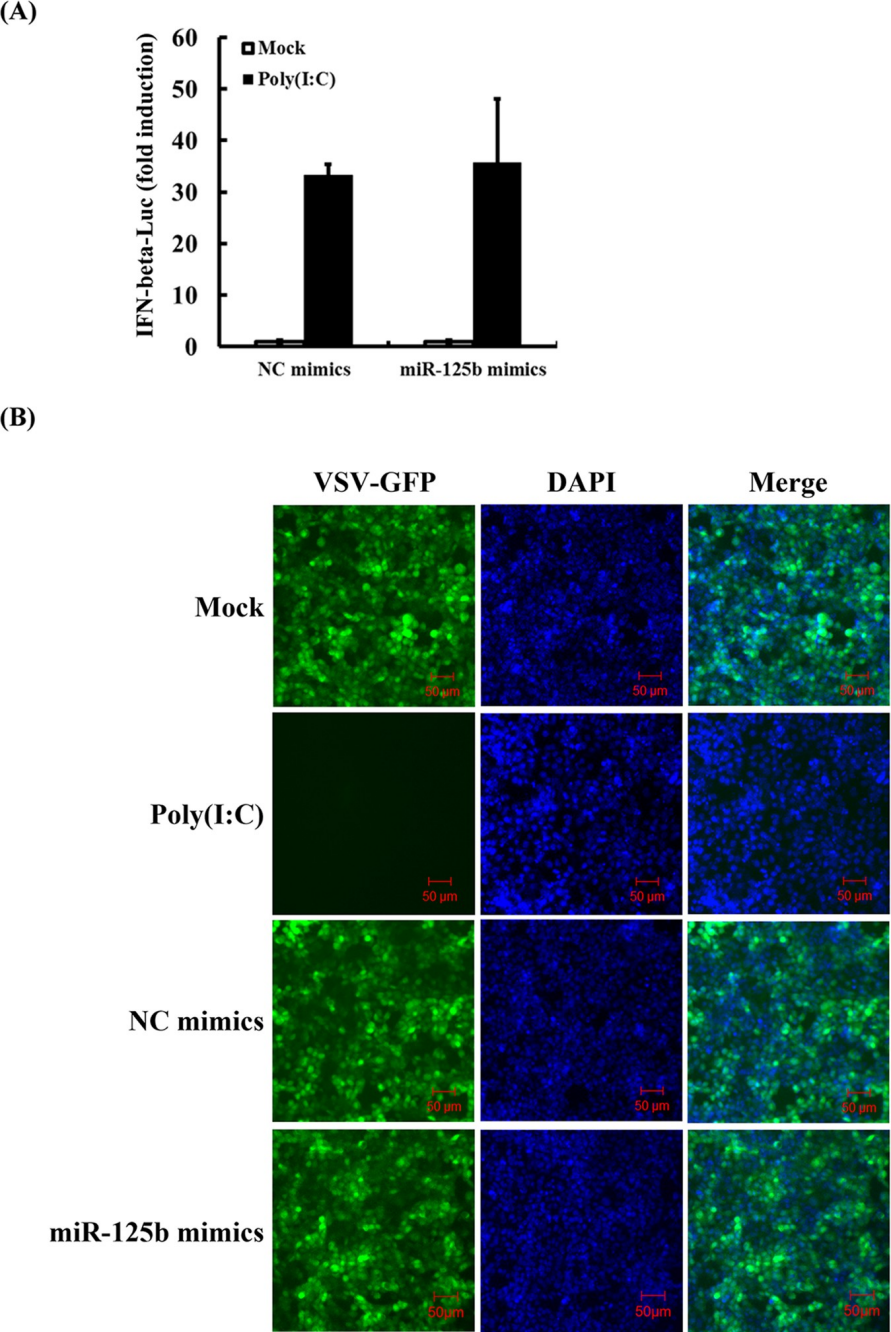
miR-125b does not induce the IFN pathway. (A) MARC-145 cells were co-transfected with IFN-β-Luc, pRL-TK, and 30 nM of miR-125b mimic or NC mimic. 24 h post-transfection, selected wells were transfected with poly(I:C) (2 µg/mL). After another 24 h, cells were lysed for dual-luciferase assay. (B) MARC-145 cells were transfected with miR-125b mimic (30 nM), NC mimic (30 nM), poly(I:C) (1 µg/mL), or mock-transfected. At 24 h post transfection, cells were infected with VSV-GFP at MOI of 0.0001. Cells were fixed at 24 h post-infection and cellular nuclei were counterstained with 1 µg/mL of DAPI. Fluorescence was observed under an LSM-510 Meta confocal fluorescence microscope (Zeiss).

**Fig 6:**
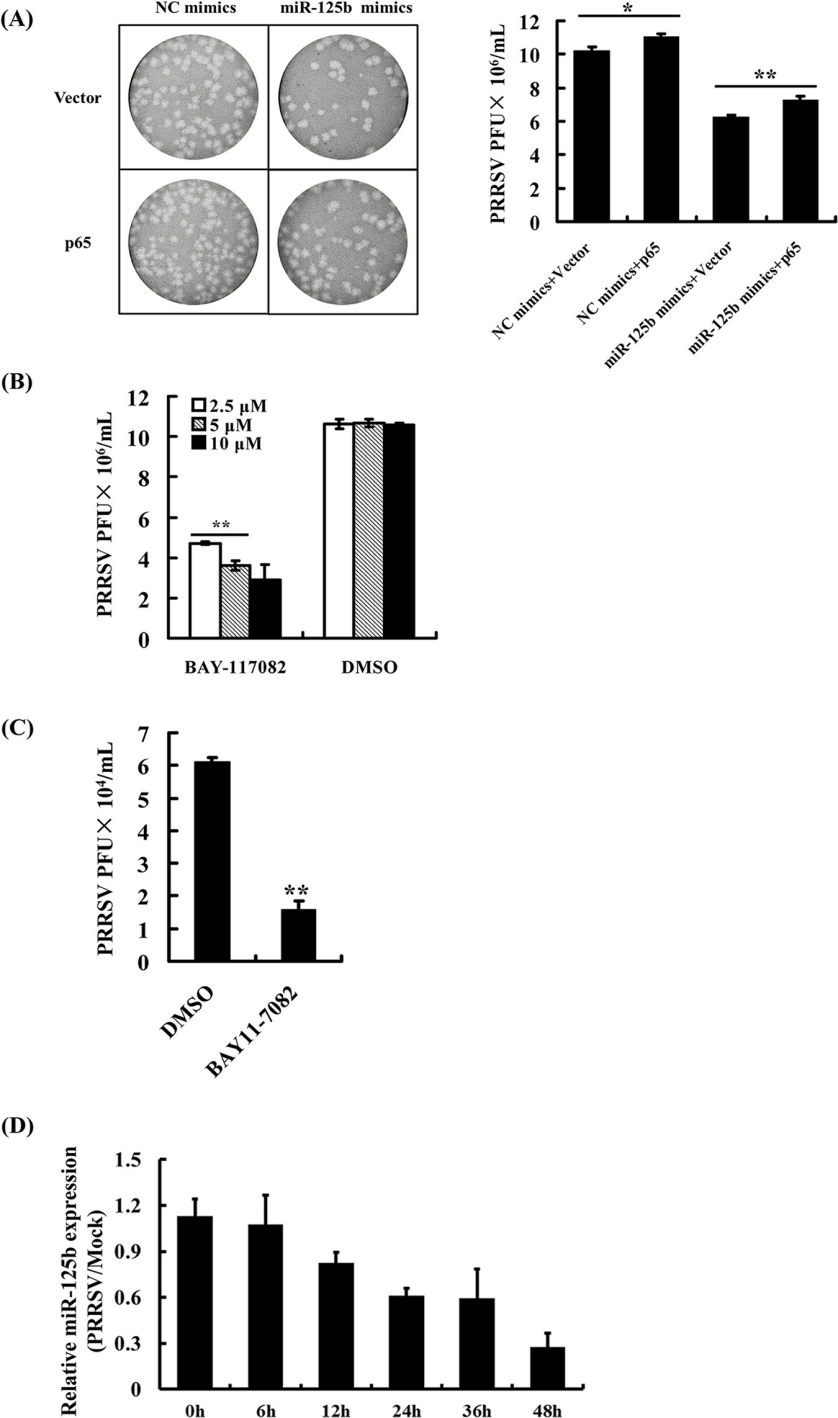
The inter-relationship among miR-125b, NF-κB activation and PRRSV replication. (A) Overexpression of the NF-κB p65 subunit promotes PRRSV replication and partially antagonizes miR-125b’s effect on PRRSV. MARC-145 cells were cotransfected with a control vector or vector encoding p65 (1.0 µg) and 60 nM of miR-125b mimic or inhibitor. The transfected cells were infected with PRRSV WUH3 strain (MOI  =  0.01) 24 h later. Cells were collected at 48 h post-infection for plaque assay on MARC-145 cells. Virus titers were expressed as PFU/mL. Representative plaque results from three independent experiments are shown in left panel and the average results are illustrated on the right. **P<0.01 and *P<0.05 as compared with cells transfected with the control vector. (B, C) Pretreatment with the NF-κB inhibitor BAY11-7082 reduces PRRSV replication in MARC-145 cells (2.5 µM, 5.0 µM and 10µM of BAY11-7082, panel B) and PAMs (5 µM, panel C). Cells were pretreated with BAY11-7082 for 1 h prior to PRRSV infection. At 48 h post-infection, cells were collected and virus titers were determined by plaque assay on MARC-145 cells. (D) The time-course expression of miR-125b after PRRSV infection. MARC-145 cells infected with PRRSV at a MOI of 0.1 were collected at the indicated time points and qRT-PCR analysis was performed to detect miR-125b expression. The miR-125b expression level at 6 h in mock-infected cells was used as the baseline (1.0) for comparison.
