# Expression of Concern: MiR-125b Reduces Porcine Reproductive and Respiratory Syndrome Virus Replication by Negatively Regulating the NF-κB Pathway

**DOI:** 10.1371/journal.pone.0354311

**Published:** 2026-07-22

**Authors:** The PLOS One Editors

Following the publication of this article [1,2], concerns were raised regarding the methodology and multiple figures. Specifically,

- The miRNA mimics and inhibitors, and qPCR primers described in [1] do not align with available sequence information for the species from which the MARC-145 cells were derived, African Green monkey (*Chlorocebus pygerythrus*) [3].
- In Fig 4B, the three Mock panels appear similar to the three miR-125b mimics panels despite representing different experimental conditions.
- The NC mimics and miR-125b mimics panels in Fig 2C appear to partially overlap and fully overlap respectively with the NC mimics p65 and miR-125b mimics Vector panels in Fig 6A.
- No background could be detected for the panels in Fig 2B.

The corresponding author stated that the NC mimics p65 and miR-125b mimics Vector panels in Fig 6A, and the three miR-125b mimics panels in Fig 4B are incorrect. Correct versions of Figs 4 and 6 are provided here where the NC mimics p65 and miR-125b mimics Vector panels in Fig 6A and the three miR-125b mimics panels in Fig 4B have been updated with the correct images from the original experiments. Original and repeat data underlying Fig 6A are provided in S1 and S4 Files, original and repeat data underlying Fig 2C are provided in S3 and S4 Files and original data underlying Fig 4B are provided in S2 File.

Regarding the plaque assays, the corresponding author stated that six-well cell culture plates (9.6 cm^2^/well) were used, the plaques in all replicate wells were counted, and for each group, plaques of one experiment were scanned to capture the representative images. They also stated that the well of the six-well cell culture plate used in these experiments was approximately 17 mm in depth, which caused shadows around the edges when images were captured by scanning. To remove the shadows, they stated that they cropped out the edges in the images at the same scale.

Original and repeat data underlying Fig 2B are provided in S3 and S4 Files. The corresponding author stated that the raw images for Fig 2B in S3 File are cropped because the software used to expose the membranes for image capture can only expose a selected area, and they selected the area near the target band’s molecular mass. The corresponding author also stated that the molecular weight of PRRSV nsp2 (~150 kDa) is larger than that of β-actin (42 kDa), and that in order to simultaneously detect nsp2 and β-actin using the same membrane blot, they cut the polyvinylidene difluoride (PVDF) membrane into two parts after protein transfer was complete. They stated that each cropped PVDF membrane piece was then incubated with primary antibodies against PRRSV nsp2 and β-actin, respectively.

The corresponding author acknowledged that miRNAs and primers were designed based on macaque monkey, human or mouse sequences, stating that there was no available miRNA sequence information corresponding to African green monkey at the time of this study. PLOS received contradictory expert input on whether miRNAs and primers designed for macaque, human and mouse would work in cells of a different species (African green monkey) given:

- miR-125b is highly conserved in macaque, human and mouse.
- Without detection of miR-125 and other African green monkey-specific miRNA expression in the MARC-145 cell line, it is not known if inhibitors are working due to targeted miRNAs as non-expression genes cannot be inhibited.
- The African green monkey cells were used as hosts for the virus, which was the target of miRNAs, and therefore the miRNAs ability to interact with the PRRSV genome and inhibit the virus remains the same.

Members of the *PLOS One* Editorial Board also raised the following concerns with [1]:

- The transfected miRNAs were not measured via qPCR to verify whether transfection had been successful or show efficiency of miR depletion.
- An internal control of non-coding RNA is needed to support the model in Fig 7, that PPRSV suppresses miR-125b.
- Fig 2C does not include statistical analysis.
- Fig 6A does not appear to show a statistical difference between plaques on cells with NC mimics+ Vector and plaques on cells with NC mimics +p65.
- The immunofluorescent studies do not appear to have been quantified.

The corresponding author stated that [1] does not examine the expression of other African green monkey-specific miRNAs in MARC-145 cells, nor evaluate the transfection efficiency of miR-125b mimics by qPCR, but does examine the expression of miR-125b in MARC-145 cells in Fig 6D. They provided additional data (S5 File) evaluating the effect of the miR-125b inhibitor on endogenous miR-125b expression. The corresponding author stated that in Fig 6D, the relative expression level of miR-125b was quantified by qPCR using U6 small nuclear RNA as the internal control for normalization as described in the qPCR for miRNA Quantification section of the Materials and Methods.

The corresponding author also stated that the numerical difference between plaques on cells with NC mimics+ Vector and plaques on cells with NC mimics +p65 in Fig 6A is small, but the intra-group variability is low. They also stated that the immunofluorescent studies were intended to provide representative visual evidence supporting the antiviral effect of miR-125b.

The article [1] reports the use of macrophages and viruses isolated from pigs, and antibodies raised in BALB/c mice, but the article does not state that ethics approval was obtained for the use of animals. The corresponding author stated that these materials were produced for earlier studies, including [4].

The corresponding author stated that the remainder of the underlying data for [1] are still available and can be provided upon request.

The Correction [2] was erroneously published before PLOS concluded the assessment of this article [1].

The *PLOS One* Editors issue this Expression of Concern due to the above concerns about the validity of the transfection and qPCR experiments due to the miRNA and primer design.

## Supporting information

S1 File Original and repeat underlying data from the time of the original experiments in support of Figure 6A. (ZIP)

S2 File Original underlying data in support of Figure 4B. (ZIP)

S3 File Original underlying data in support of Figure 2B, and original and repeat underlying data from the time of the original experiments in support of Figure 2C. (ZIP)

S4 File Underlying data from later repeat experiments in support of Figures 2C and 6A, and the western blots in Figure 2B. (ZIP)

S5 File Additional data from earlier experiments evaluating the effect of the miR-125b inhibitor on endogenous miR-125b expression. (XLS)

S6 File miR-125b primer sequences for qPCR. (DOCX)

**Fig 4:**
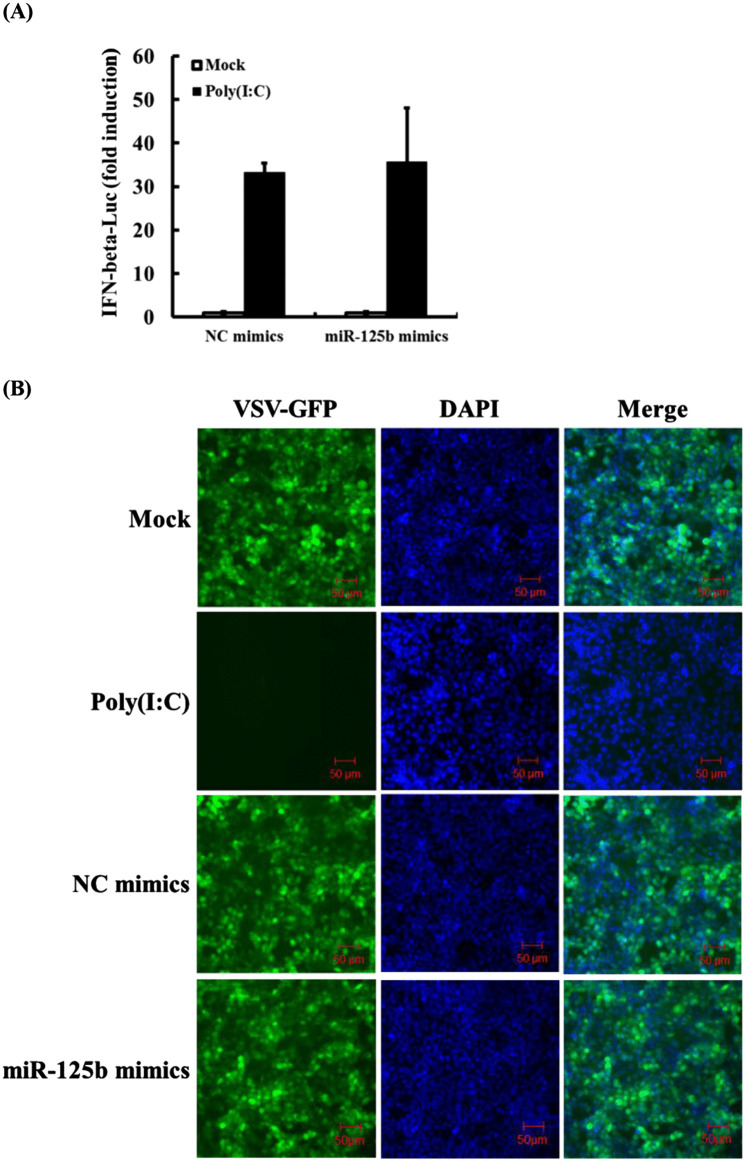
miR-125b does not induce the IFN pathway. (A) MARC-145 cells were co-transfected with IFN-β-Luc, pRL-TK, and 30 nM of miR-125b mimic or NC mimic. 24 h post-transfection, selected wells were transfected with poly(I:C) (2 µg/mL). After another 24 h, cells were lysed for dualluciferase assay. (B) MARC-145 cells were transfected with miR-125b mimic (30 nM), NC mimic (30 nM), poly(I:C) (1 µg/mL), or mock-transfected. At 24 h post transfection, cells were infected with VSV-GFP at MOI of 0.0001. Cells were fixed at 24 h post-infection and cellular nuclei were counterstained with 1 µg/mL of DAPI. Fluorescence was observed under an LSM-510 Meta confocal fluorescence microscope (Zeiss).

**Fig 6:**
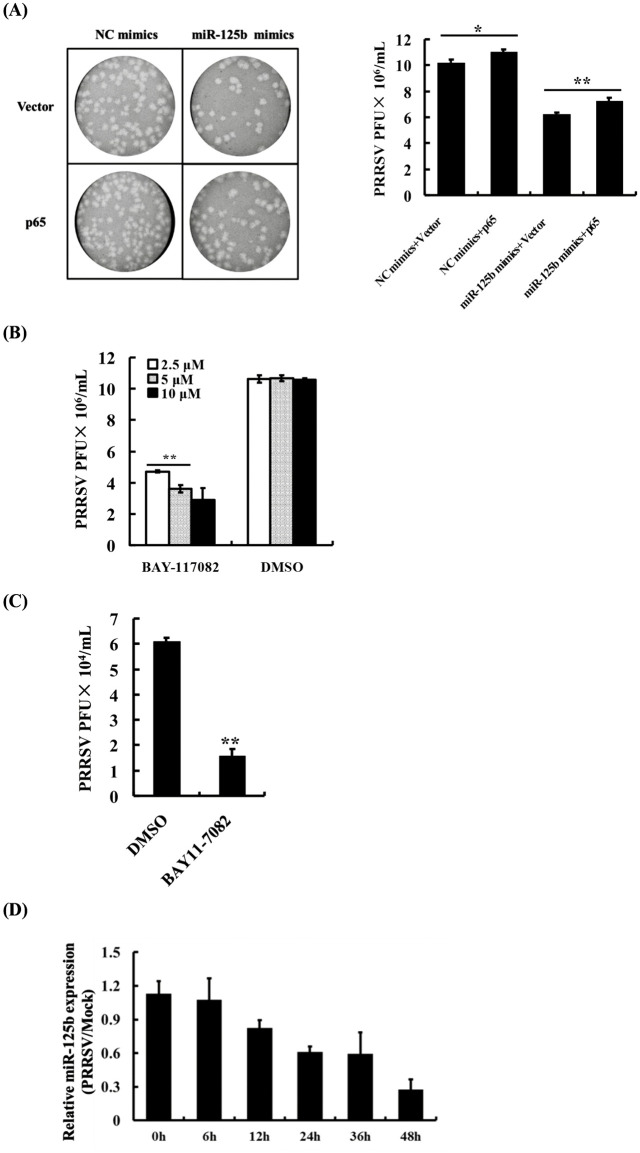
The inter-relationship among miR-125b, NF-κB activation and PRRSV replication. (A) Overexpression of the NF-κB p65 subunit promotes PRRSV replication and partially antagonizes miR-125b’s effect on PRRSV. MARC-145 cells were cotransfected with a control vector or vector encoding p65 (1.0 µg) and 60 nM of miR-125b mimic or inhibitor. The transfected cells were infected with PRRSV WUH3 strain (MOI = 0.01) 24 h later. Cells were collected at 48 h post-infection for plaque assay on MARC-145 cells. Virus titers were expressed as PFU/mL. Representative plaque results from three independent experiments are shown in left panel and the average results are illustrated on the right. **P < 0.01 and *P < 0.05 as compared with cells transfected with the control vector. (B, C) Pretreatment with the NF-κB inhibitor BAY11-7082 reduces PRRSV replication in MARC-145 cells (2.5 µM, 5.0 µM and 10µM of BAY11-7082, panel B) and PAMs (5 µM, panel C). Cells were pretreated with BAY11-7082 for 1 h prior to PRRSV infection. At 48 h post-infection, cells were collected and virus titers were determined by plaque assay on MARC-145 cells. (D) The time-course expression of miR-125b after PRRSV infection. MARC-145 cells infected with PRRSV at a MOI of 0.1 were collected at the indicated time points and qRT-PCR analysis was performed to detect miR-125b expression. The miR-125b expression level at 6 h in mock-infected cells was used as the baseline (1.0) for comparison.
