# Supplementary material for: MiR-125b Reduces Porcine Reproductive and Respiratory Syndrome Virus Replication by Negatively Regulating the NF-κB Pathway
Source: PLoS One. 2013 Feb 7;8(2):e55838. doi: 10.1371/journal.pone.0055838 (PMC3566999; doi:10.1371/journal.pone.0055838)
Supplement: Table S1 — The sequences of microRNA (miRNA) mimics and inhibitors used in this study. (DOC) [file pone.0055838.s001.doc]

**Table S1.** The sequences of microRNA (miRNA) mimics and inhibitors used in this study.

| miRNA | Mimic sequence (5’−3’) | Inhibitor sequence (5’−3’) |
| --- | --- | --- |
| miR-146a | ugagaacugaauuccauggguu | AACCCAUGGAAUUCAGUUCUCA |
| miR-351 | ucccugaggagcccuuugagccug | CAGGCUCAAAGGGCUCCUCAGGGA |
| miR-181a | aacauucaacgcugucggugagu | CUCACCGACAGCGUUGAAUGUU |
| miR-155 | uuaaugcuaauugugauaggggu | ACCCCUAUCACAAUUAGCAUUAA |
| miR-125b | UCCCUGAGACCCUAACUUGUGA | UCACAAGUUAGGGUCUCAGGGA |
| miR-122a | UGGAGUGUGACAAUGGUGUUUG | CAAACACCAUUGUCACACACUCCA |
| miR-93 | AAAGUGCUGUUCGUGCAGGUAG | CUACCUGCACGAACAGCACUUU |
| miR-196b | uagguaguuuccuguuguuggg | CCCAACAACAGGAAACUACCUA |
| miR-24 | UGGCUCAGUUCAGCAGGAACAG | CUGUUCCUGCUGAACUGAGCCA |
| miR-365 | UAAUGCCCCUAAAAAUCCUUAU | AUAAGGAUUUUUAGGGGCAUUA |
| Control | UUCUCCGAACGUGUCACGUTT | CAGUACUUUUGUGUAGUACAA |
