# Supplementary material for: MiR-125b Reduces Porcine Reproductive and Respiratory Syndrome Virus Replication by Negatively Regulating the NF-κB Pathway
Source: PLoS One. 2013 Feb 7;8(2):e55838. doi: 10.1371/journal.pone.0055838 (PMC3566999; doi:10.1371/journal.pone.0055838)
Supplement: Table S2 — Sequence of oligonucleotide primers used in this study. (DOC) [file pone.0055838.s002.doc]

**Table S2.** Sequence of oligonucleotide primers used in this study.

| Primer | Sequence (5’−3’) |
| --- | --- |
| 5’UTR-F | GCGACTAGTATGACGTATAGGTGTTGGC |
| 5’UTR-R | ATAAAGCTTTGGTTAAAGGGGTGGAGAG |
| nsp1α-F | GCGGAGCTCATGTCTGGGATACTTGATCGGTGCAC |
| nsp1α-R | ATAAAGCTTCTGCGGGAGCGGCAAGTTGGTTAAC |
| nsp1β-F | GCGACTAGTAGGCCCAAACCTGAGGACTTTTGCCC |
| nsp1β-R | ATAAAGCTTACCGTACCACTTATGACTGCCAAACC |
| nsp2-F | ACAACTAGTGCCGGAAAGAGAGCAAGGAAAACACG |
| nsp2-R | ACAACGCGTGCCCAGTAACCTGCCAAGAATGGCAA |
| nsp3-F | GCGACTAGTGGGGCACGCTACATCTGGCACTTT |
| nsp3-R | ATAAAGCTTCTCAAGGAGGGACCCGAGCTGAGA |
| nsp4-F | ATAACTAGTGGCGCTTTCAGAACTCAAAAGCCCTC |
| nsp4-R | ATAAAGCTTTTCCAGTTCGGGTTTGGCAGCAAGCA |
| nsp5-F | ATAACTAGTGGAGGCCTTTCCACAGTTCAACTTCT |
| nsp5-R | GCGAAGCTTCTCGGCAAAGTATCGCAAGAAGAAAG |
| nsp6-F | GCGACTAGTATGACGTATAGGTGTTGGC |
| nsp6-R | ATAAAGCTTTGGTTAAAGGGGTGGAGAG |
| nsp7-F | ATAACTAGTTCGCTGACTGGTGCCCTCGCCATGAG |
| nsp7-R | GCGAAGCTTTTCCCACTGAGCTCTTCTATTCTCG |
| nsp8-F | ATAACTAGTGCCGCCAAGCTTTCCGTGGAGCAAG |
| nsp8-R | GCGACGCGTCTAGCAGTTTAAACACTGCTCCTTAG |
| nsp9-F | ACAACTAGTGGAGCAGTGTTTAAACTGCTAGCCGC |
| nsp9-R | GAGAAGCTTCTCATGATTGGACCTGAGTTTTTCCC |
| nsp10-F | GCGACTAGTGGGAAGAAGTCCAGAATGTGCGGGTA |
| nsp10-R | ATAAAGCTTTTCCAGGTCTGCGCAAATAGCGCGGA |
| nsp11-F | ATAACTAGTGGGTCGAGCTCCCCGCTCCCCAAG |
| nsp11-R | GCGAAGCTTTTCAAGTTGGAAATAGGCCGTCTTG |
| nsp12-F | AGAACTAGTGGCCGCCATTTTACCTGGTATCAACT |
| nsp12-R | TCAAAGCTTTCAATTCAGGCCTAAAGTTGGTTCAA |
| ORF2a-F | GAGACTAGTATGAAATGGGGTCTATGCAAAGCCTC |
| ORF2a-R | GAGACGCGTTCACCATGAGTTCAAAAGAAAAGTTG |
| ORF2b-F | TATACTAGTATGGGGTCTATGCAAAGCCTCTTTGA |
| ORF2b-R | GCGAAGCTTTCATAAGATCTTCTGTAATTGCTCAG |
| ORF3-F | GGCACTAGTATGGCTAATAGCTGTACATTCCTCCA |
| ORF3-R | TCAAAGCTTCTATCGCCGTGCGGCACTGAGAAATT |
| ORF4-F | GGCACTAGTATGGCTGCGTCCTTTCTTTTCCTCTT |
| ORF4-R | TCGAAGCTTTCAAATTGCCAGTAGGGATGGCAAAA |
| ORF5-F | ATAACTAGTATGTTGGGGAAGTGCTTGACCGCGTG |
| ORF5-R | TAAAAGCTTCTAGAGACGACCCCATAGTTCCGCTG |
| ORF6-F | ATAACTAGTGAACTATGGGGTCGTCTCTAG |
| ORF6-R | GGCAAGCTTCTTGCCGTTGTTATTTGGCAT |
| ORF7-F | GGCACTAGTATGCCAAATAACAACGGCAAGCAG |
| ORF7-R | TAAAAGCTTTCATGCTGAGGGTGATGCTGTGGC |
| 3'UTR-F | GGCGAGCTCTGGGCTGGCATTCTTT |
| 3'UTR-R | GGCAAGCTTTTAATTACGGCCGCATGG |

UTR, untranslated region
