# Supplementary figures and images for: Expression of Concern: MiR-125b Reduces Porcine Reproductive and Respiratory Syndrome Virus Replication by Negatively Regulating the NF-κB Pathway
Source: PLoS One. 2026 Jul 22;21(7):e0354311. doi: 10.1371/journal.pone.0354311 (PMC13390827; doi:10.1371/journal.pone.0354311)

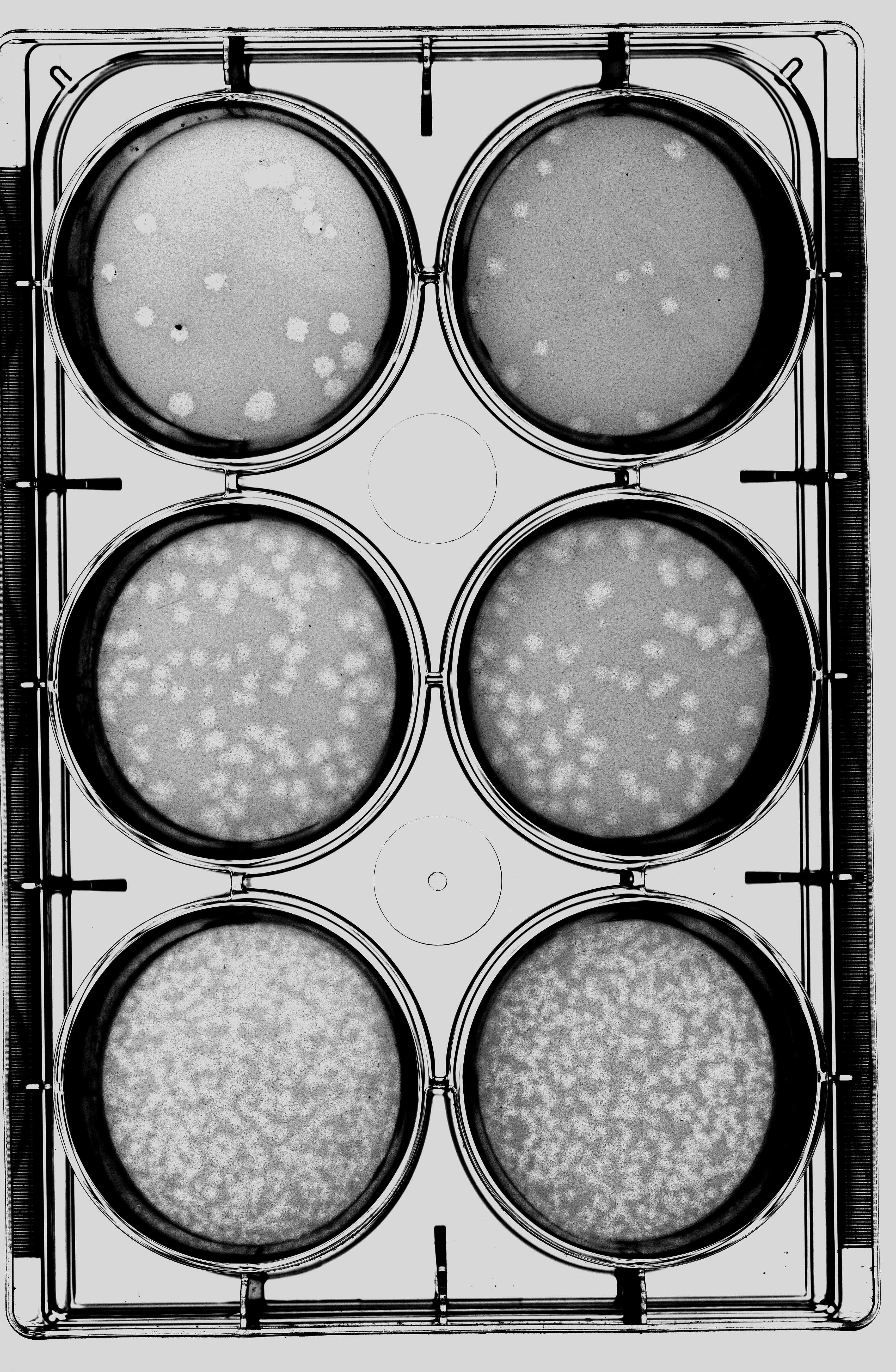

Supplement: S1 File — (ZIP) [file pone.0354311.s001.zip › Figure 6/Fig 6A_plaque assay_p65 reversed PRRSV replication/Fig 6A_plaque assay image-1_p65 reversed PRRSV replication.jpg]

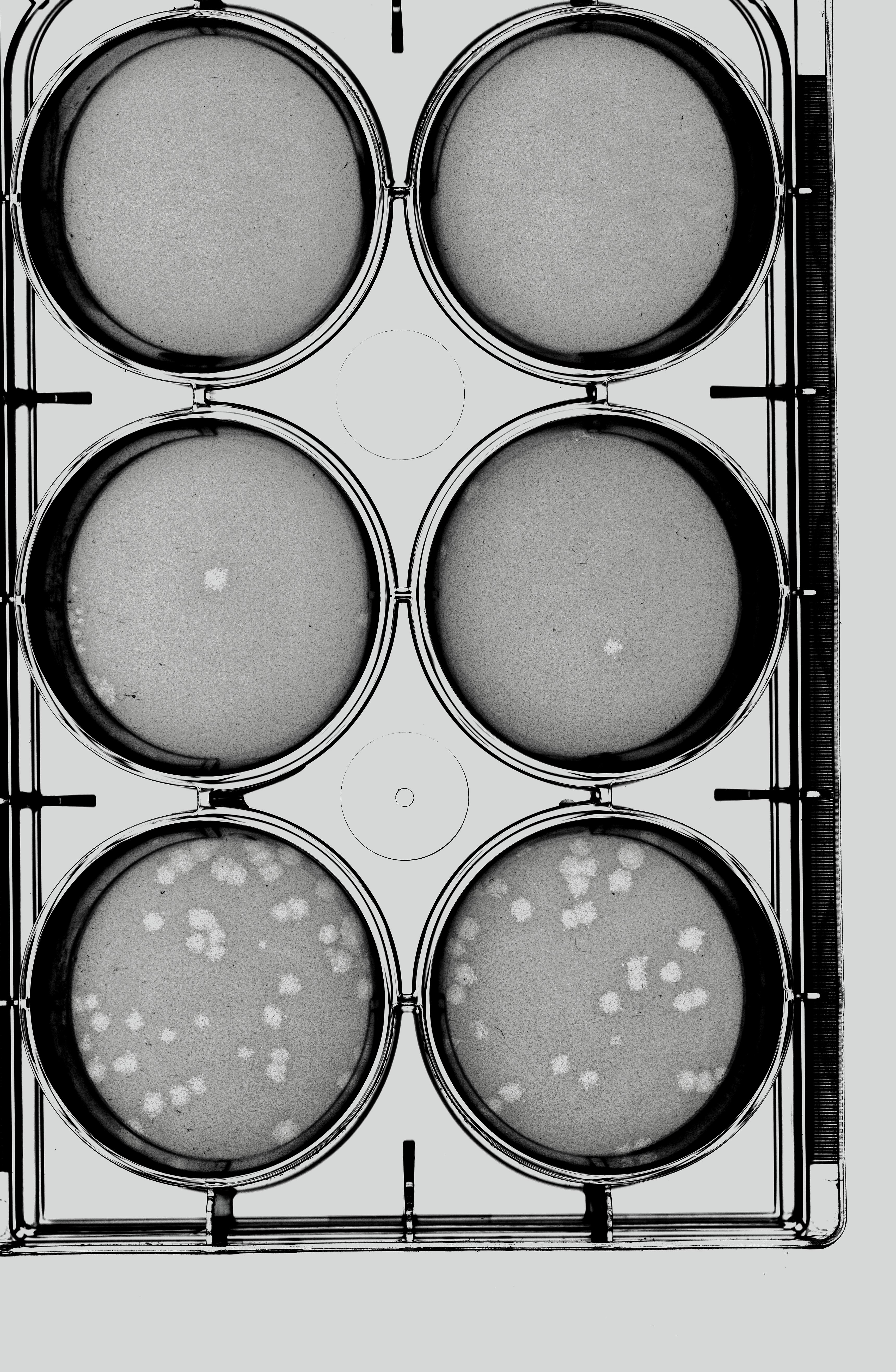

Supplement: S1 File — (ZIP) [file pone.0354311.s001.zip › Figure 6/Fig 6A_plaque assay_p65 reversed PRRSV replication/Fig 6A_plaque assay image-2_p65 reversed PRRSV replication.jpg]

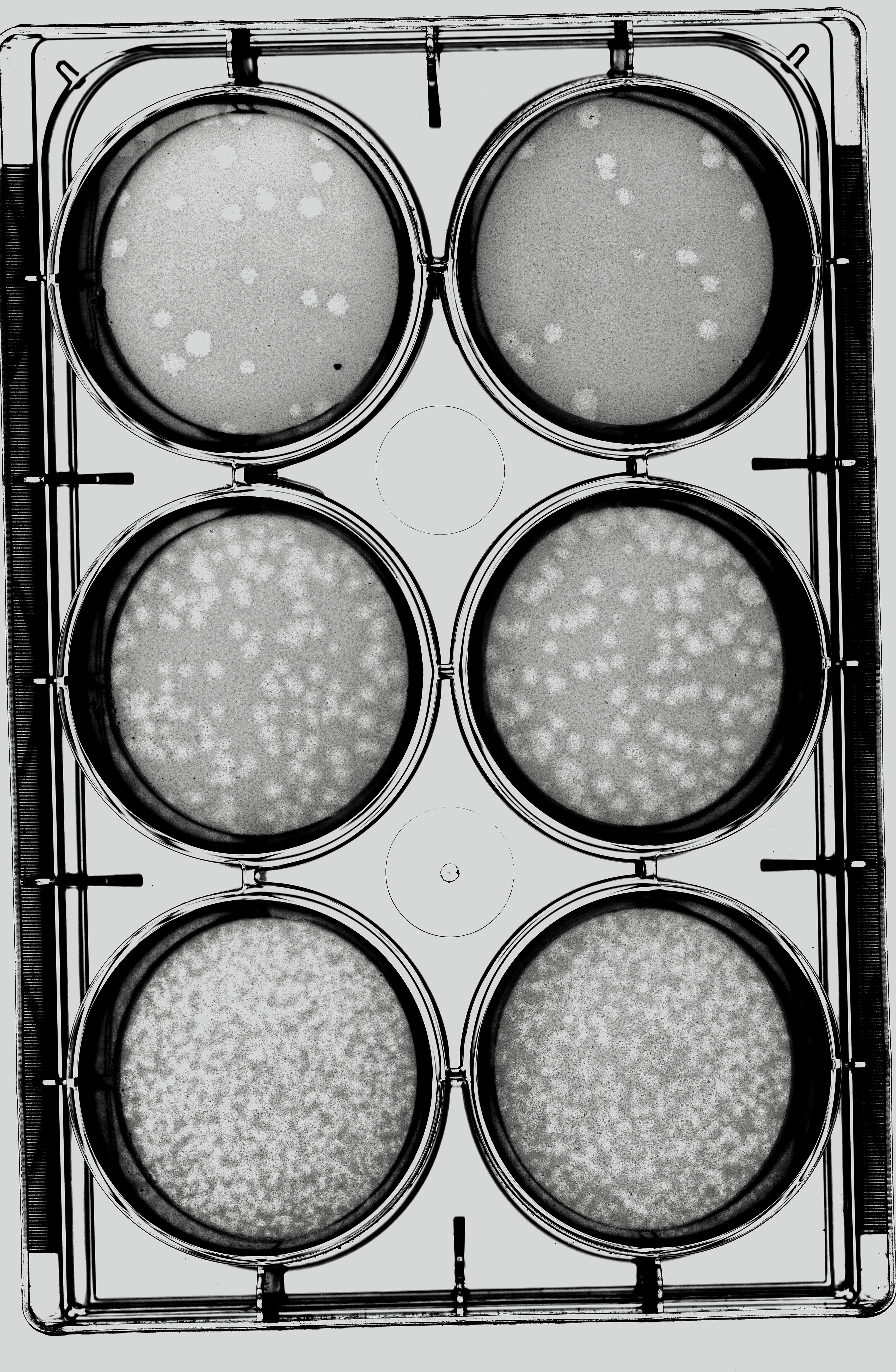

Supplement: S1 File — (ZIP) [file pone.0354311.s001.zip › Figure 6/Fig 6A_plaque assay_p65 reversed PRRSV replication/Fig 6A_plaque assay image-3_p65 reversed PRRSV replication.jpg]

## Slide 1
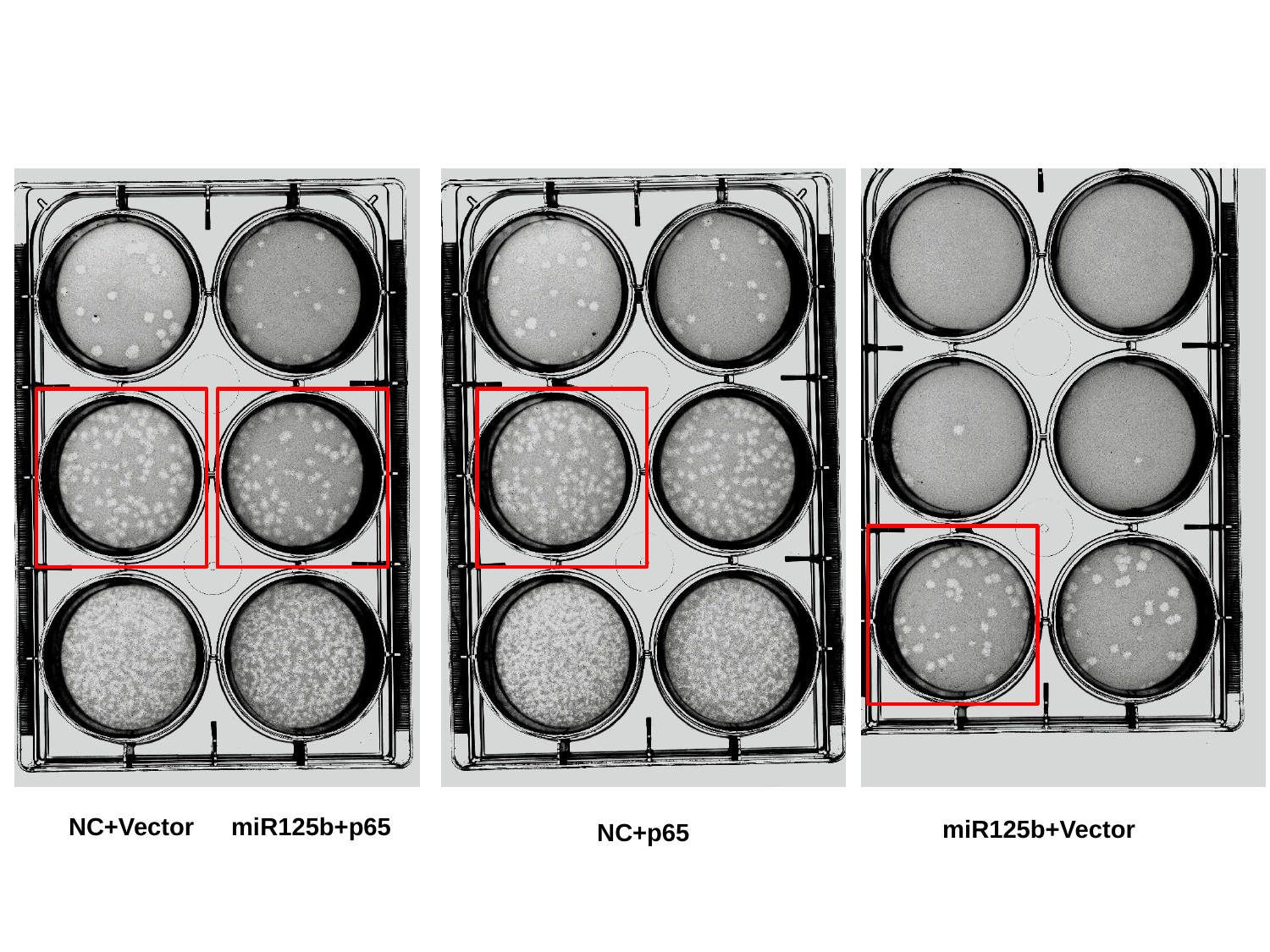

NC+Vector
miR125b+p65
miR125b+Vector
NC+p65

Supplement: S1 File — (ZIP) [file pone.0354311.s001.zip › Figure 6/Fig 6A_plaque assay_p65 reversed PRRSV replication/Fig 6A_plaque assay image_description_p65 reversed PRRSV replication.pptx]

## Slide 1
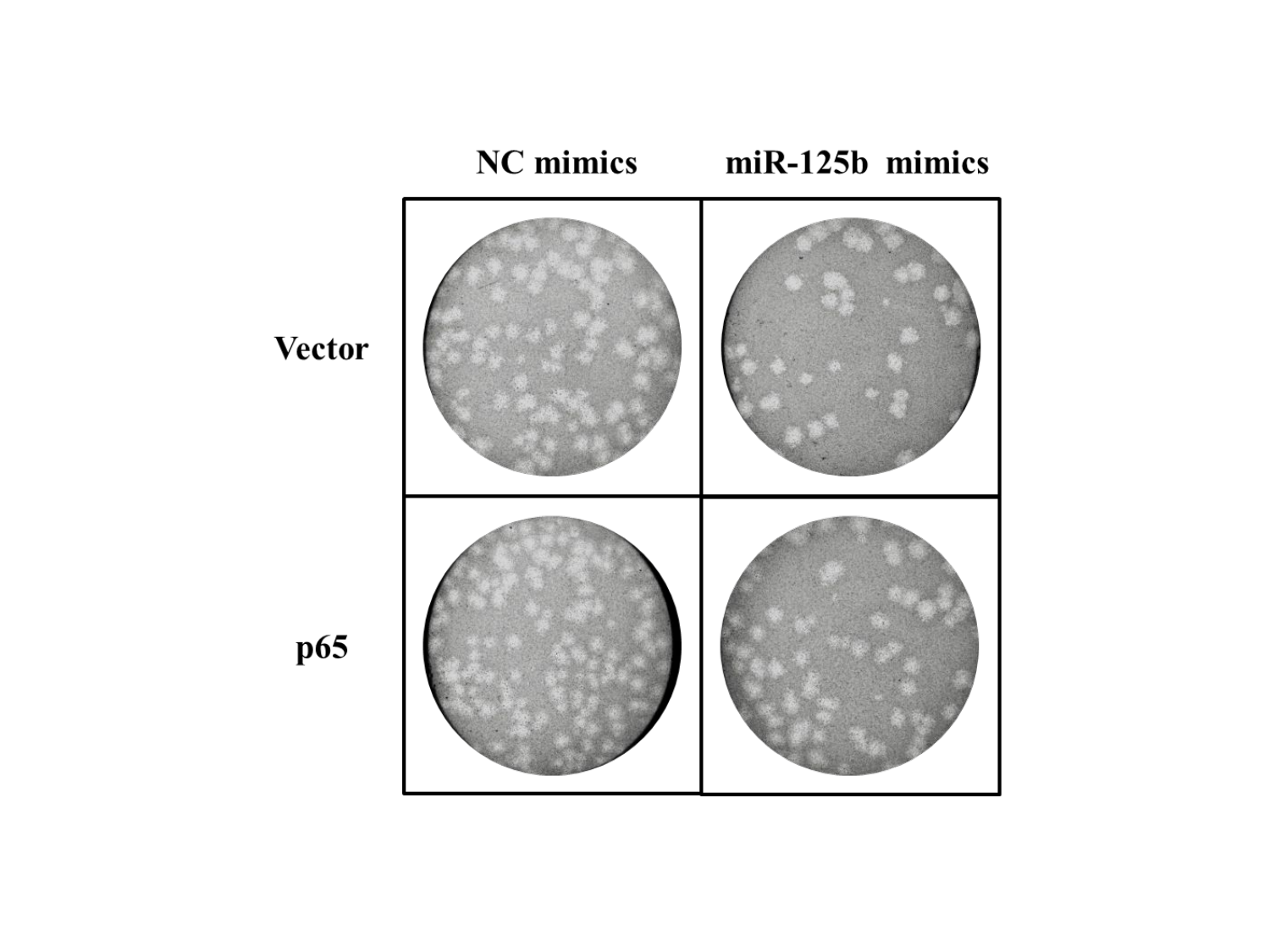

Supplement: S1 File — (ZIP) [file pone.0354311.s001.zip › Figure 6/Fig 6A_plaque assay_p65 reversed PRRSV replication/Updated Figure 6A (plaque assay image).pptx]

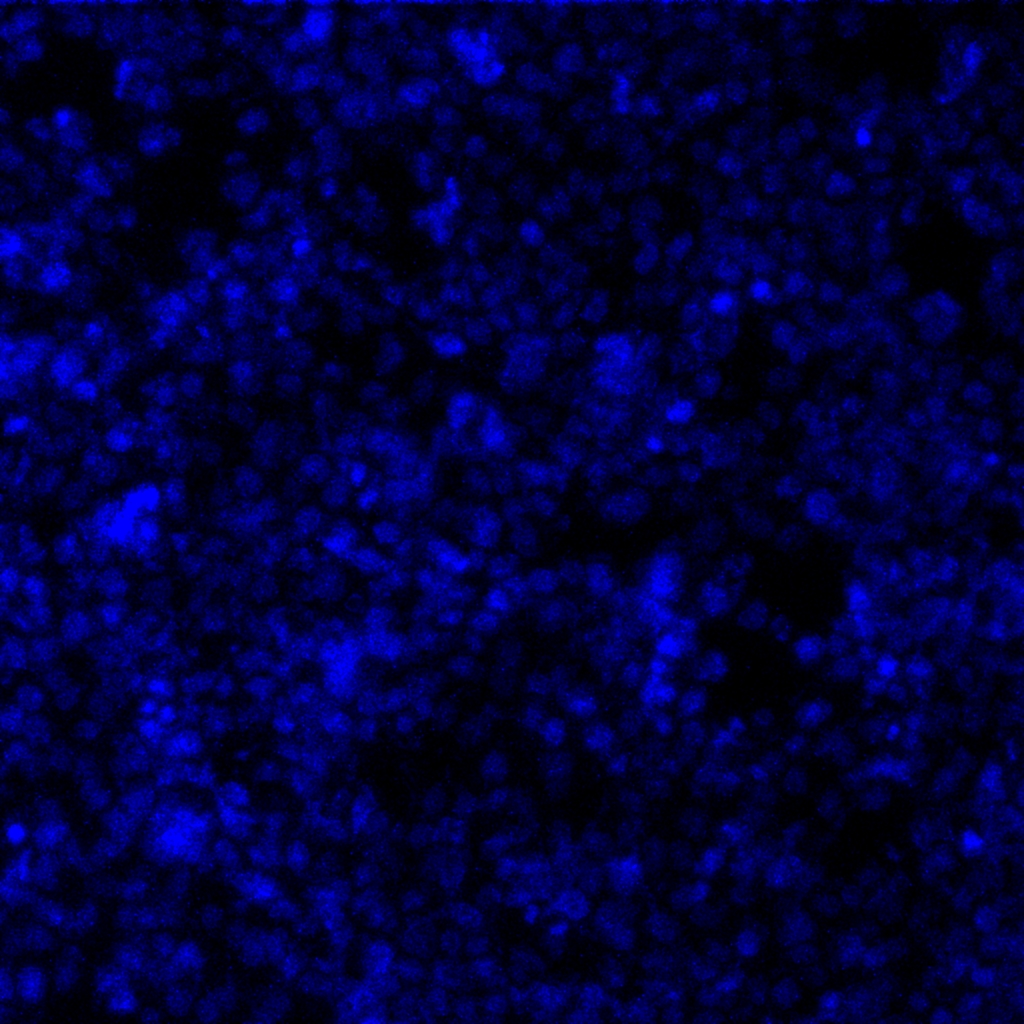

Supplement: S2 File — (ZIP) [file pone.0354311.s002.zip › Figure 4/Fig 4B_IFA_miR-125b does not inhibit VSV-GFP replication/Fig 4B_Mock_IFA_miR-125b does not inhibit VSV-GFP replication/Fig 4B_Mock_DAPI.jpg]

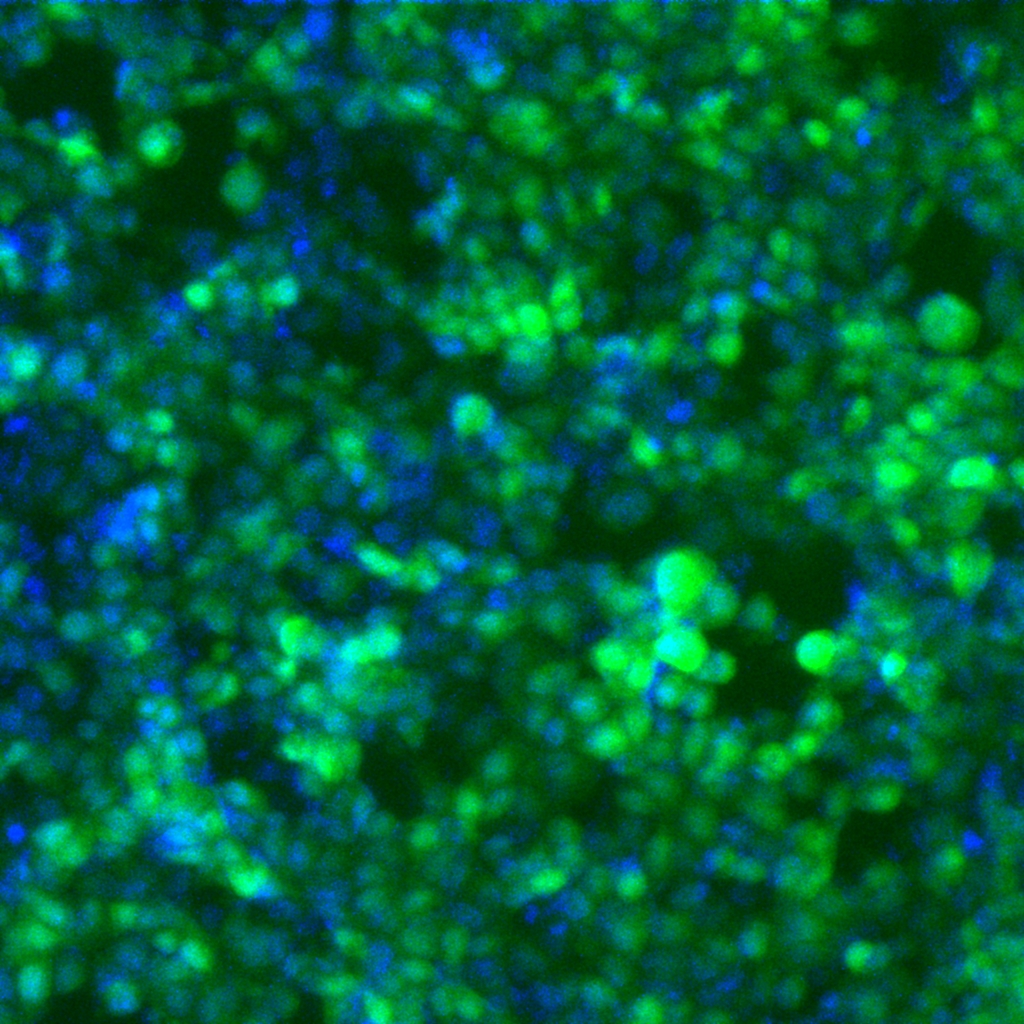

Supplement: S2 File — (ZIP) [file pone.0354311.s002.zip › Figure 4/Fig 4B_IFA_miR-125b does not inhibit VSV-GFP replication/Fig 4B_Mock_IFA_miR-125b does not inhibit VSV-GFP replication/Fig 4B_Mock_Merge.jpg]

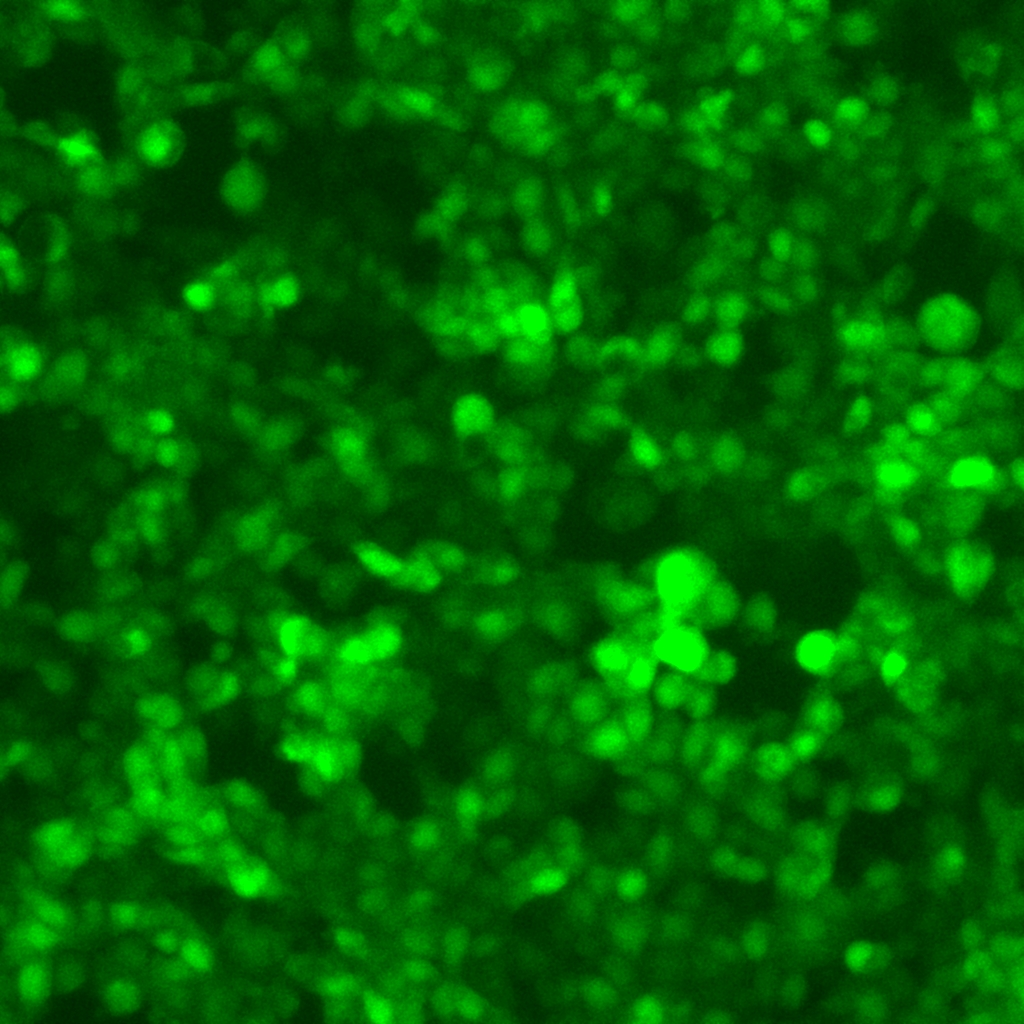

Supplement: S2 File — (ZIP) [file pone.0354311.s002.zip › Figure 4/Fig 4B_IFA_miR-125b does not inhibit VSV-GFP replication/Fig 4B_Mock_IFA_miR-125b does not inhibit VSV-GFP replication/Fig 4B_Mock_VSV-GFP.jpg]

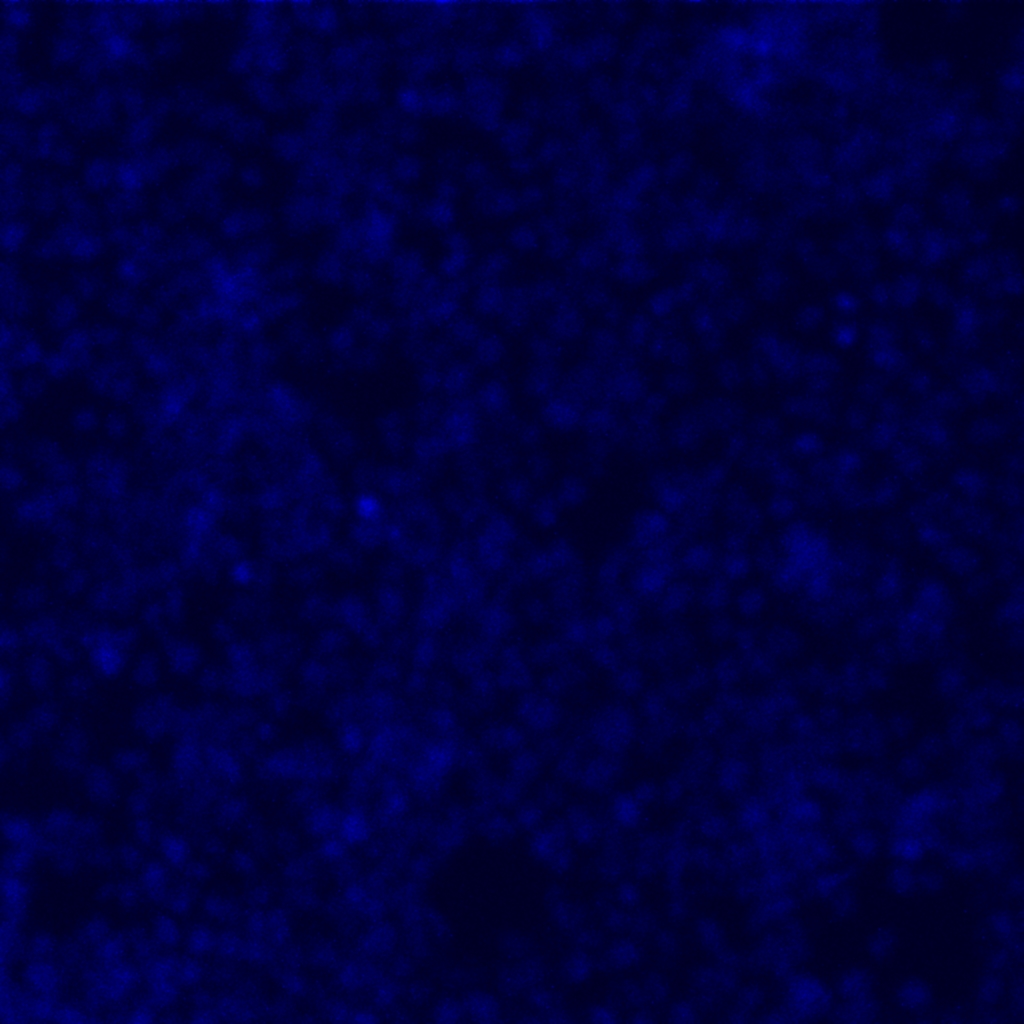

Supplement: S2 File — (ZIP) [file pone.0354311.s002.zip › Figure 4/Fig 4B_IFA_miR-125b does not inhibit VSV-GFP replication/Fig 4B_NC mimics_IFA_miR-125b does not inhibit VSV-GFP replication/Fig 4B_NC mimics_DAPI.jpg]

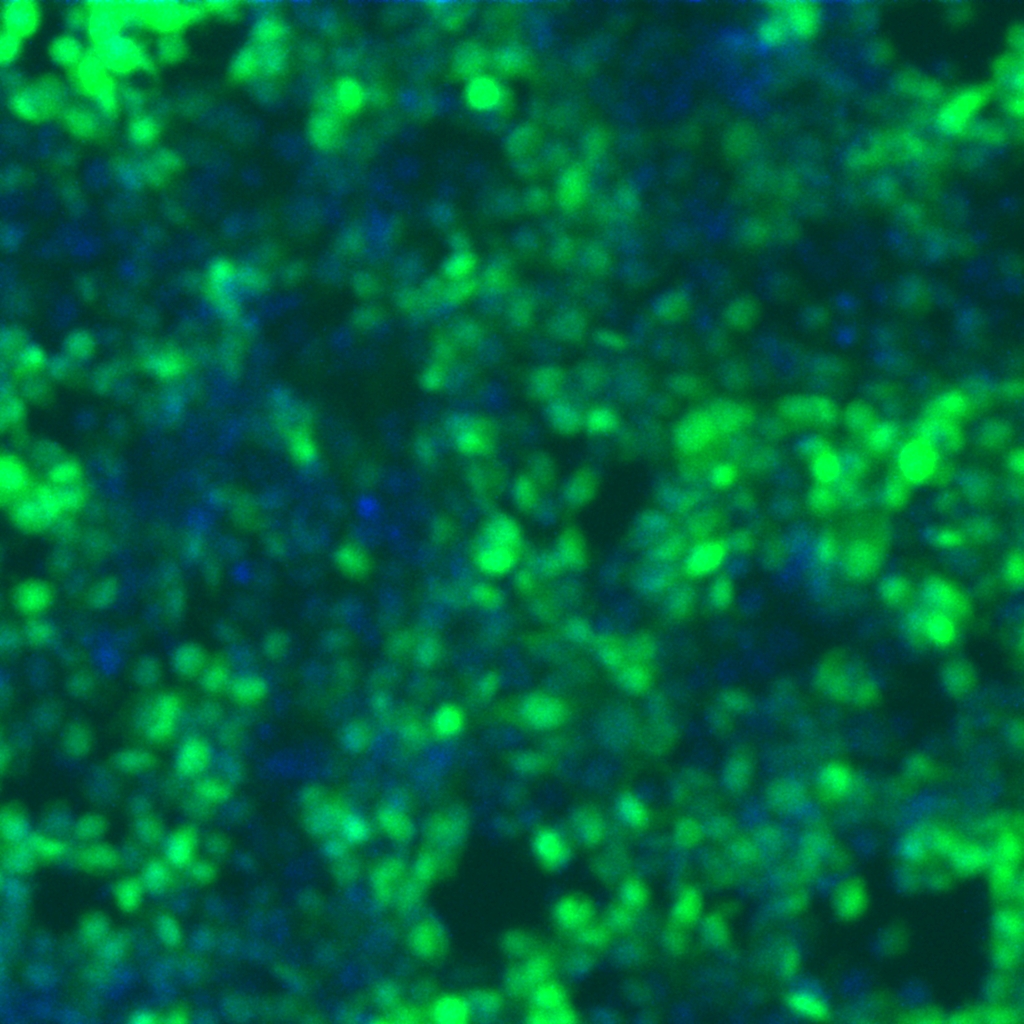

Supplement: S2 File — (ZIP) [file pone.0354311.s002.zip › Figure 4/Fig 4B_IFA_miR-125b does not inhibit VSV-GFP replication/Fig 4B_NC mimics_IFA_miR-125b does not inhibit VSV-GFP replication/Fig 4B_NC mimics_Merge.jpg]

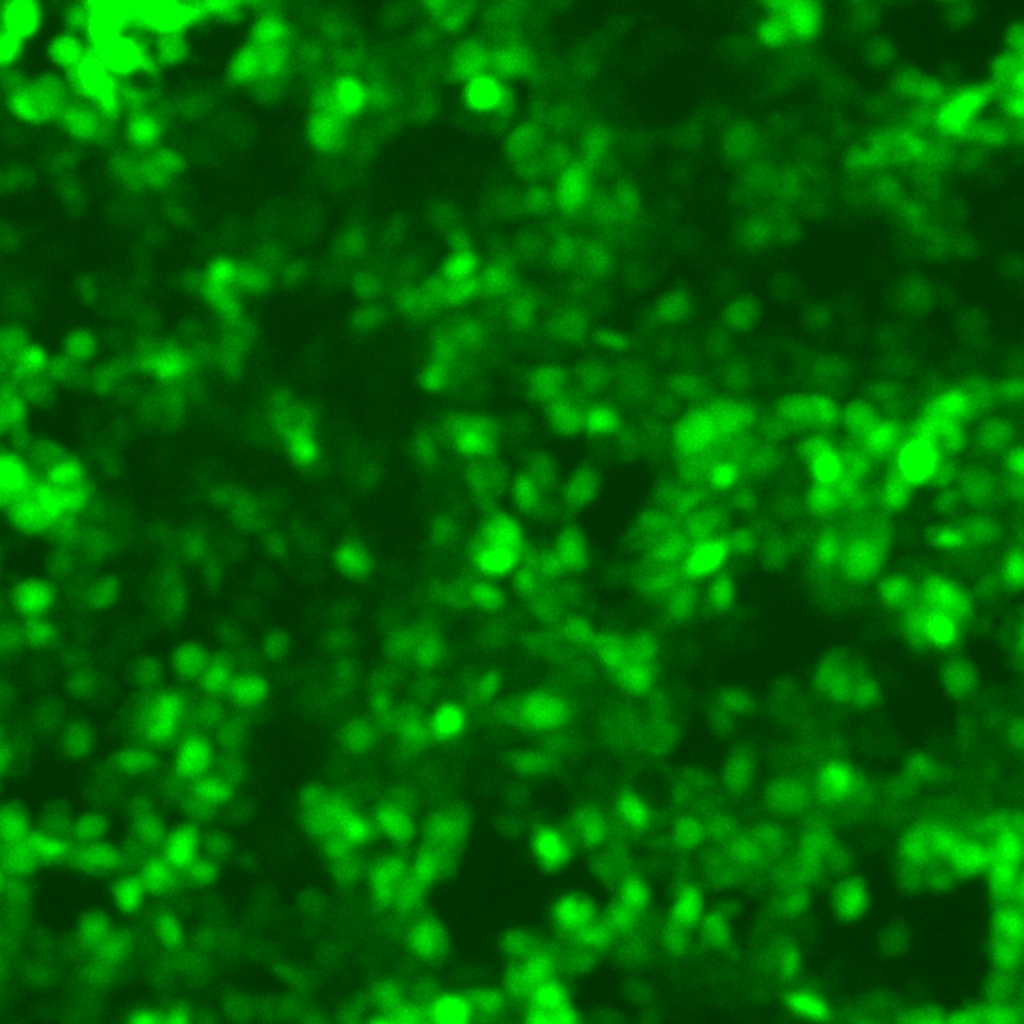

Supplement: S2 File — (ZIP) [file pone.0354311.s002.zip › Figure 4/Fig 4B_IFA_miR-125b does not inhibit VSV-GFP replication/Fig 4B_NC mimics_IFA_miR-125b does not inhibit VSV-GFP replication/Fig 4B_NC mimics_VSV-GFP.jpg]

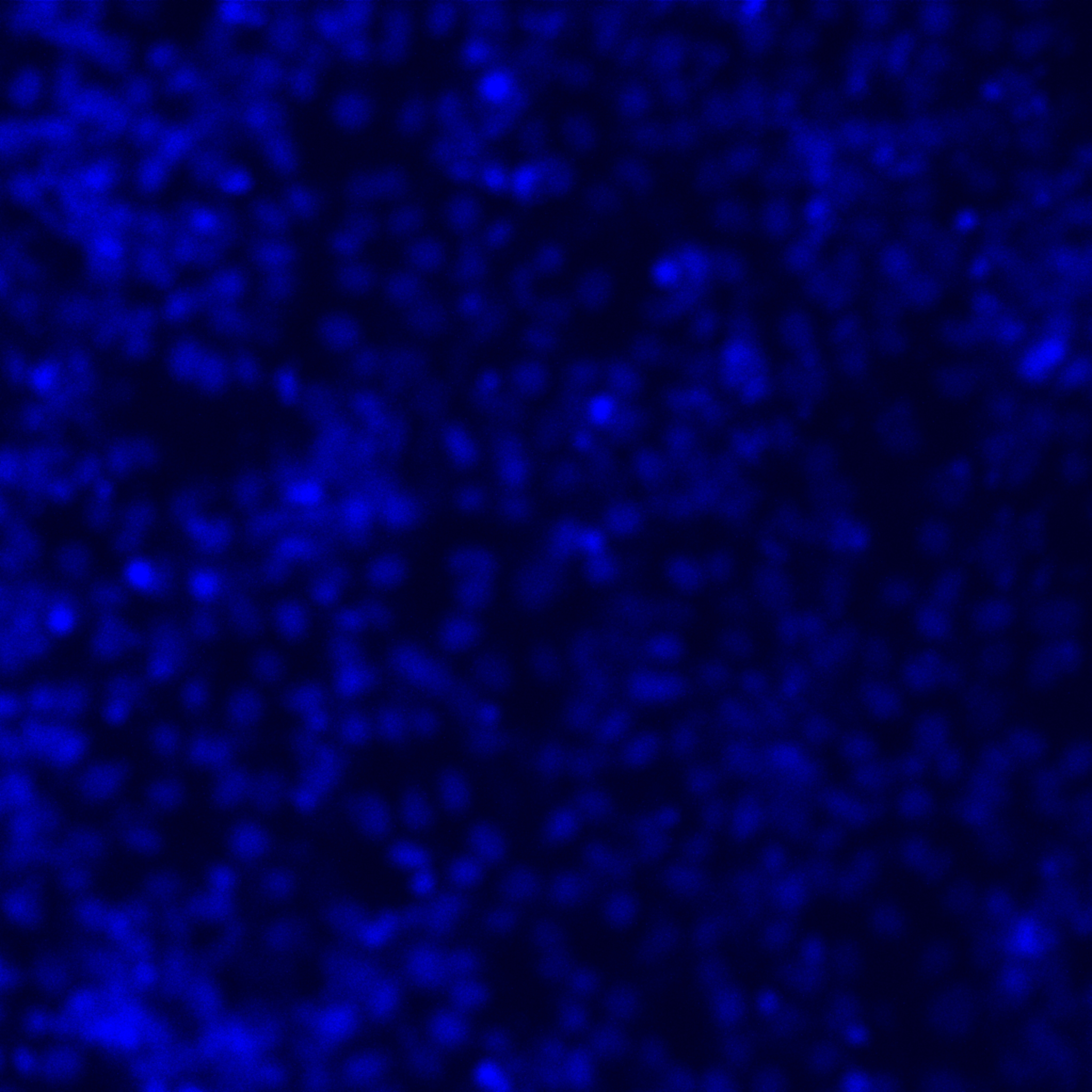

Supplement: S2 File — (ZIP) [file pone.0354311.s002.zip › Figure 4/Fig 4B_IFA_miR-125b does not inhibit VSV-GFP replication/Fig 4B_Poly(IC)_IFA_miR-125b does not inhibit VSV-GFP replication/Fig 4B_Poly(IC)_DAPI.jpg]

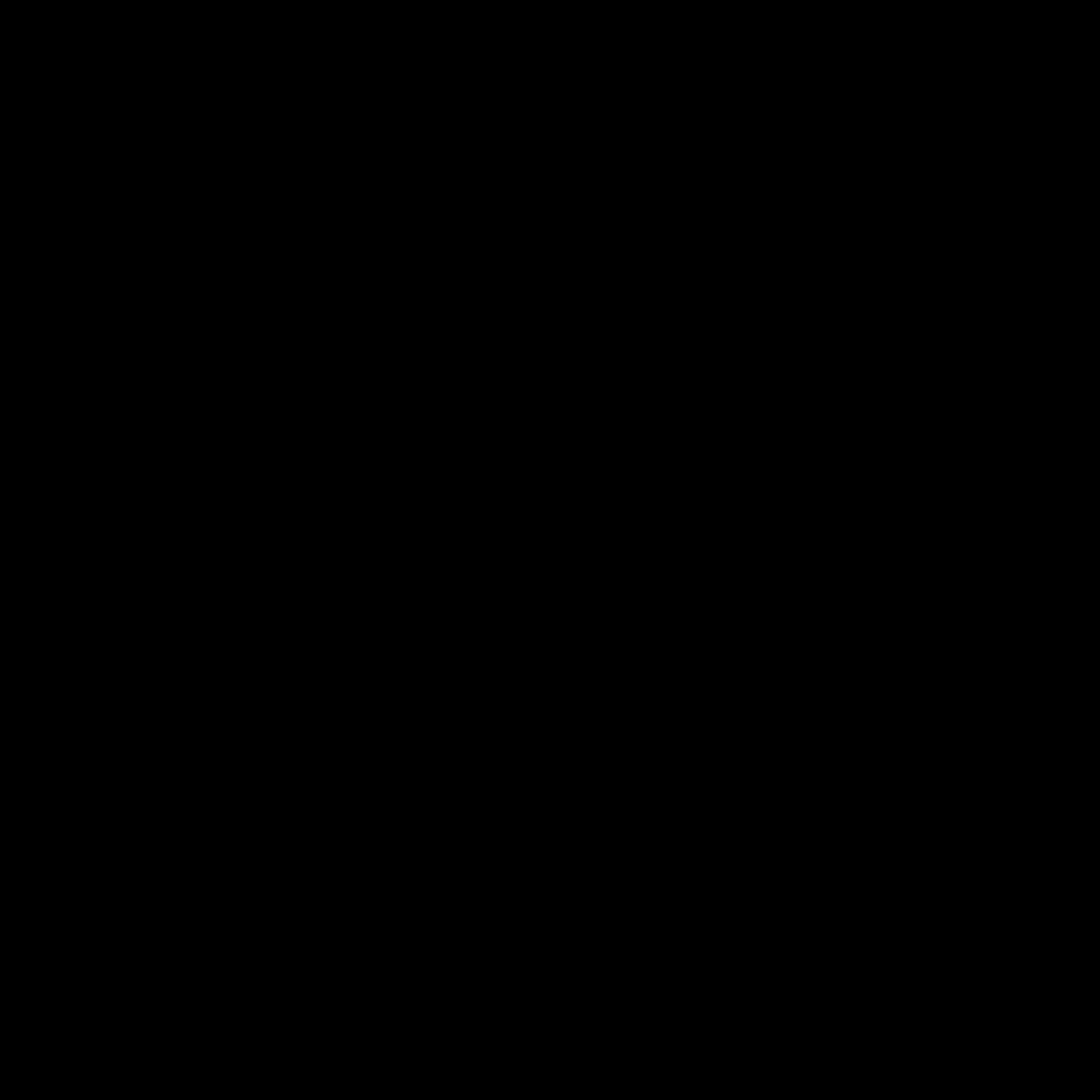

Supplement: S2 File — (ZIP) [file pone.0354311.s002.zip › Figure 4/Fig 4B_IFA_miR-125b does not inhibit VSV-GFP replication/Fig 4B_Poly(IC)_IFA_miR-125b does not inhibit VSV-GFP replication/Fig 4B_Poly(IC)_VSV-GFP.jpg]

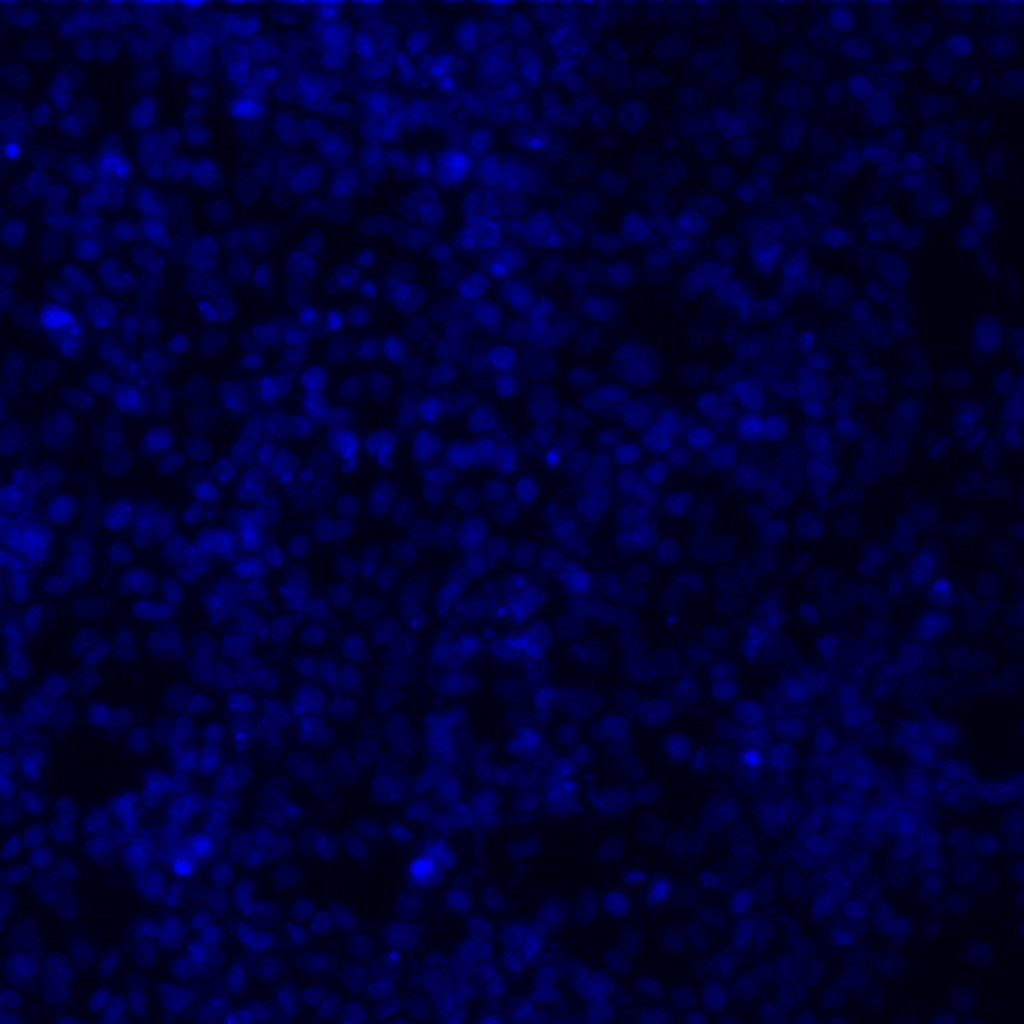

Supplement: S2 File — (ZIP) [file pone.0354311.s002.zip › Figure 4/Fig 4B_IFA_miR-125b does not inhibit VSV-GFP replication/Fig 4B_miR-125b mimics_IFA_miR-125b does not inhibit VSV-GFP replication/Fig 4B_miR-125b mimics_DAPI.jpg]

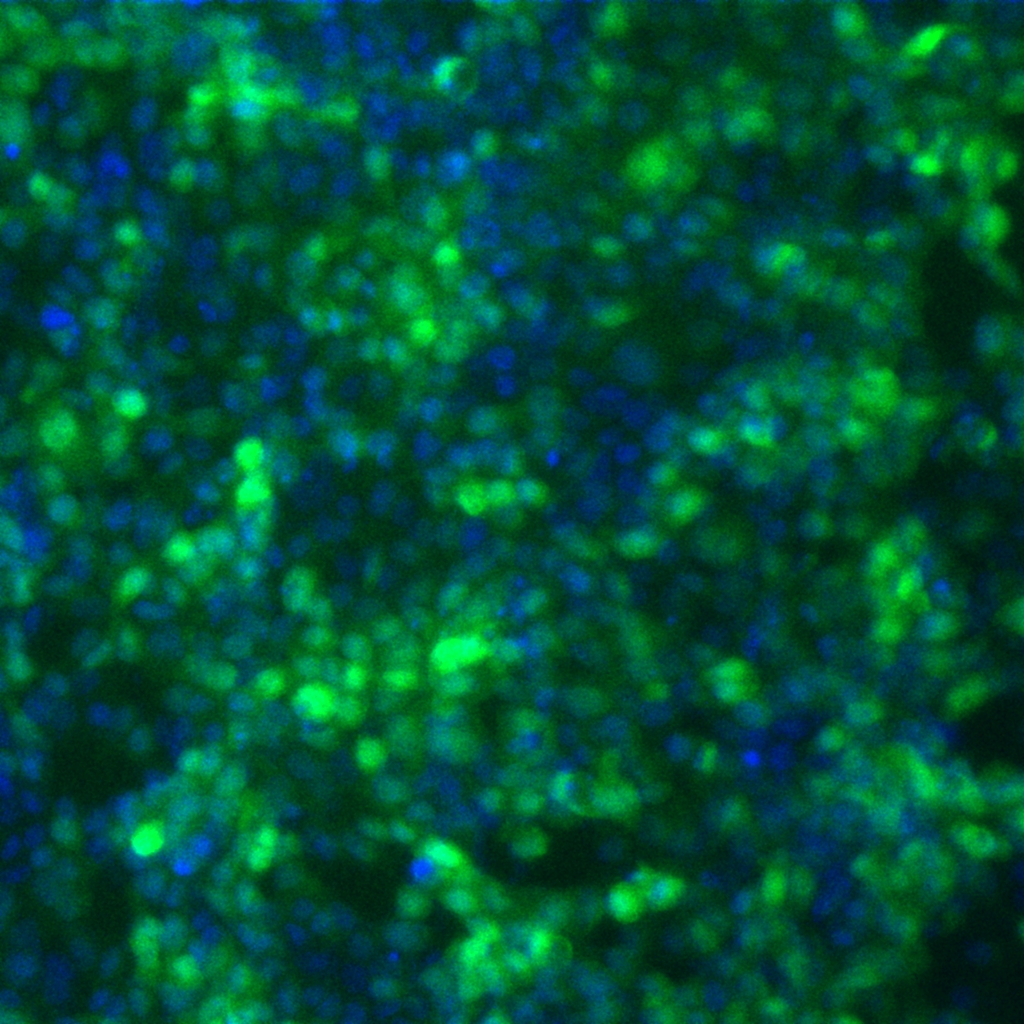

Supplement: S2 File — (ZIP) [file pone.0354311.s002.zip › Figure 4/Fig 4B_IFA_miR-125b does not inhibit VSV-GFP replication/Fig 4B_miR-125b mimics_IFA_miR-125b does not inhibit VSV-GFP replication/Fig 4B_miR-125b mimics_Merge.jpg]

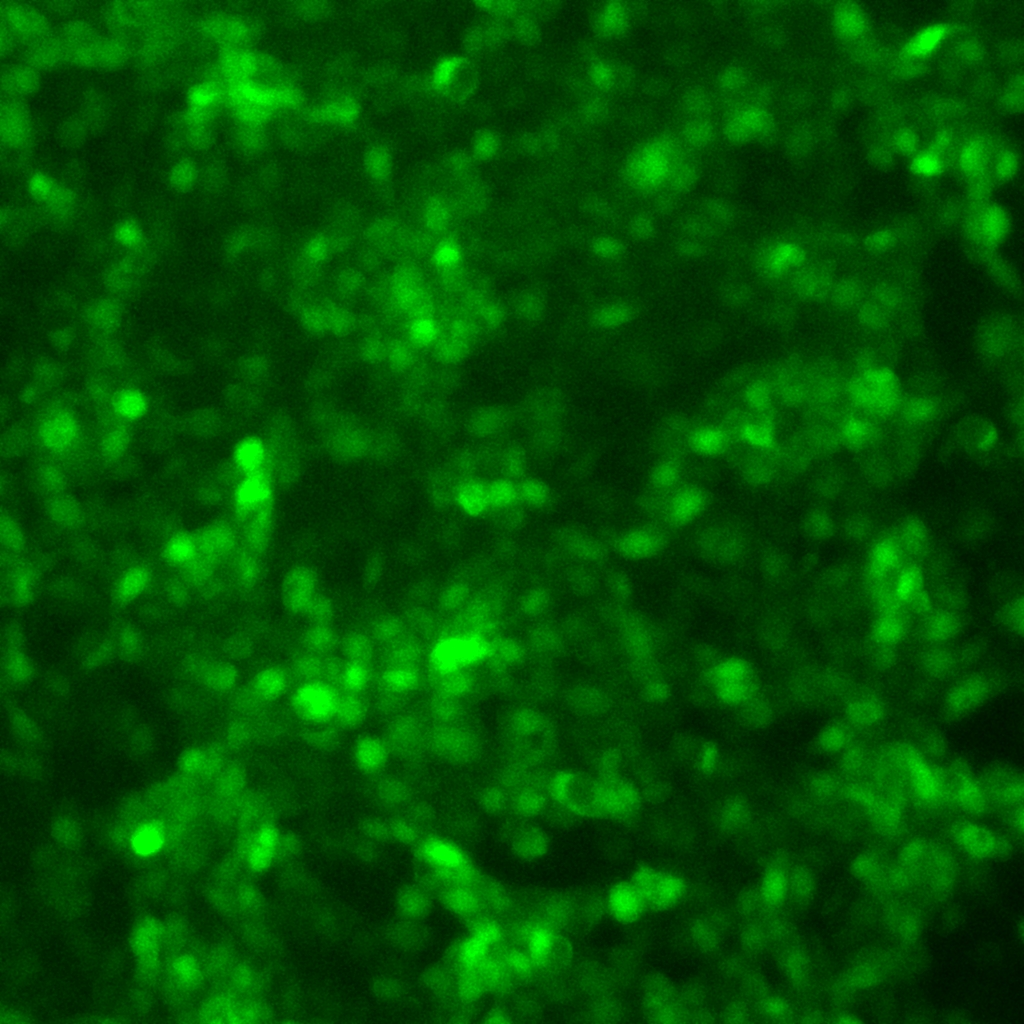

Supplement: S2 File — (ZIP) [file pone.0354311.s002.zip › Figure 4/Fig 4B_IFA_miR-125b does not inhibit VSV-GFP replication/Fig 4B_miR-125b mimics_IFA_miR-125b does not inhibit VSV-GFP replication/Fig 4B_miR-125b mimics_VSV-GFP.jpg]

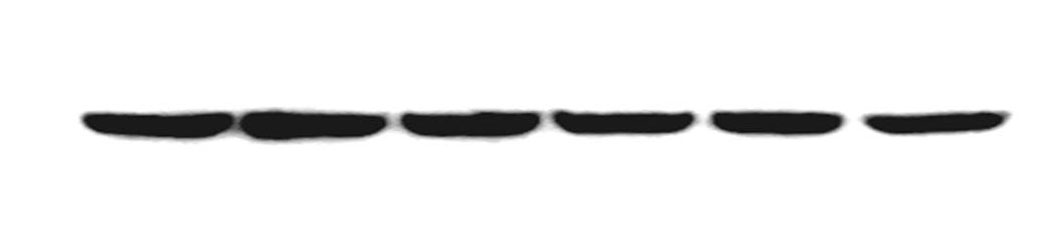

Supplement: S3 File — (ZIP) [file pone.0354311.s003.zip › Figure 2/Fig 2B_blot_miR-125b inhibits PRRSV in MARC-145/Fig 2B_Beta actin blot_miR-125b inhibits PRRSV in MARC-145.jpg]

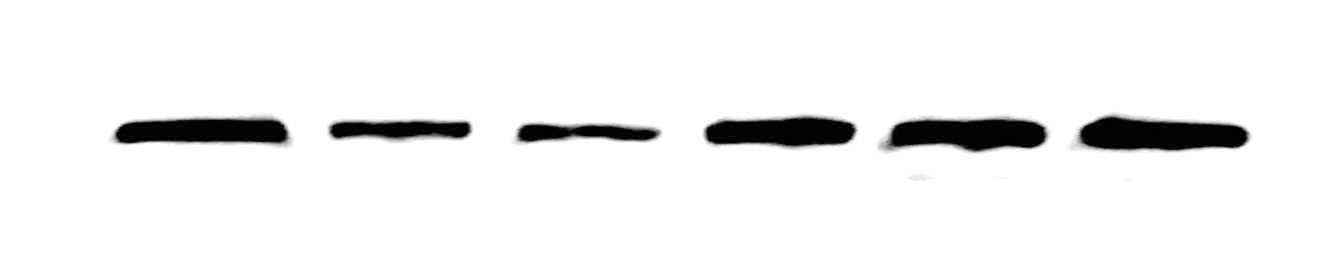

Supplement: S3 File — (ZIP) [file pone.0354311.s003.zip › Figure 2/Fig 2B_blot_miR-125b inhibits PRRSV in MARC-145/Fig 2B_nsp2 blot_miR-125b inhibits PRRSV in MARC-145.jpg]

## Slide 1
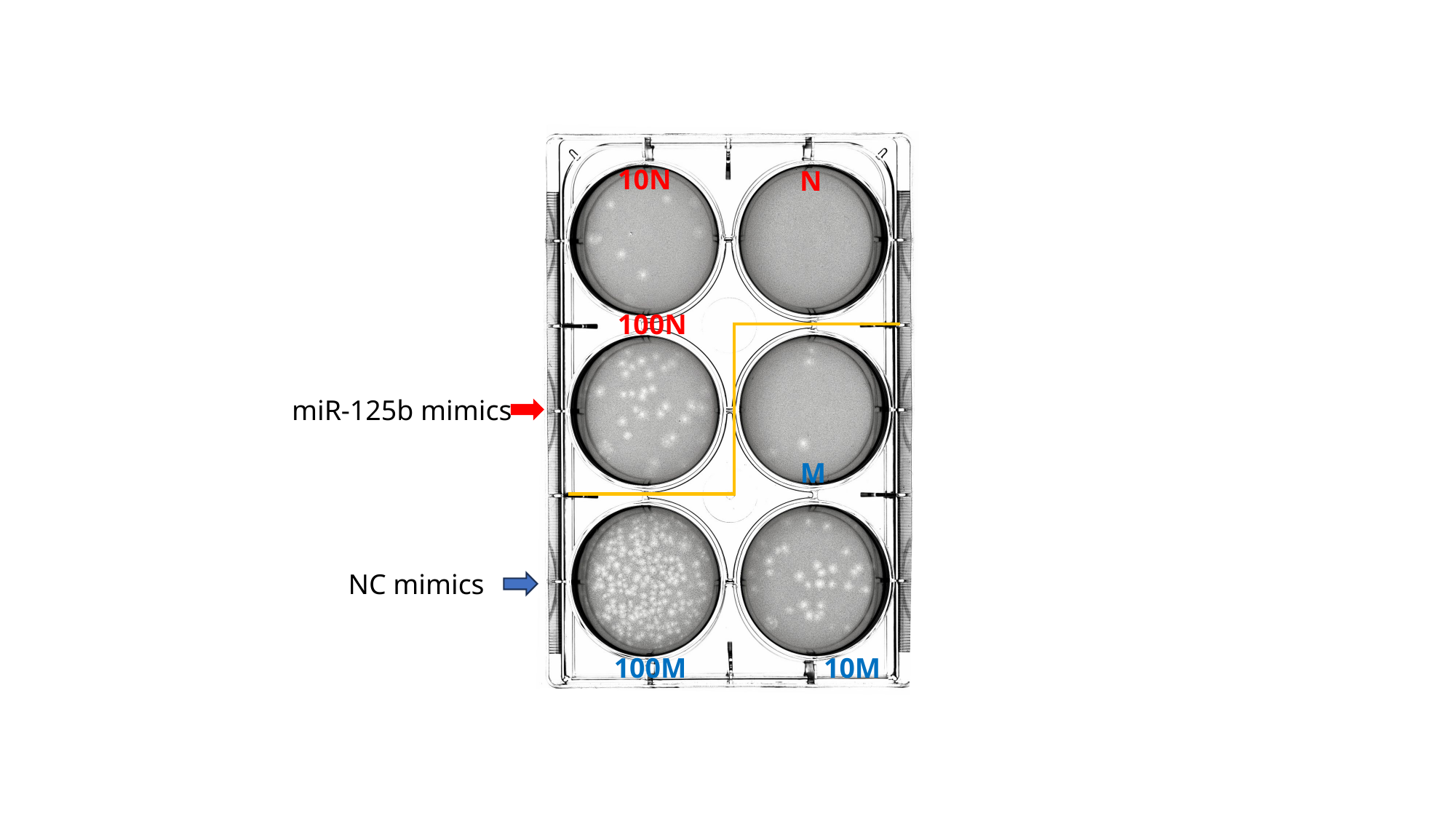

10N
N
100N
miR-125b mimics
M
NC mimics
10M
100M

Supplement: S3 File — (ZIP) [file pone.0354311.s003.zip › Figure 2/Fig 2C_plaque assay_miR-125b inhibits PRRSV in PAMs/Fig 2C_plaque assay image_description_miR-125b inhibits PRRSV in PAMs.pptx]

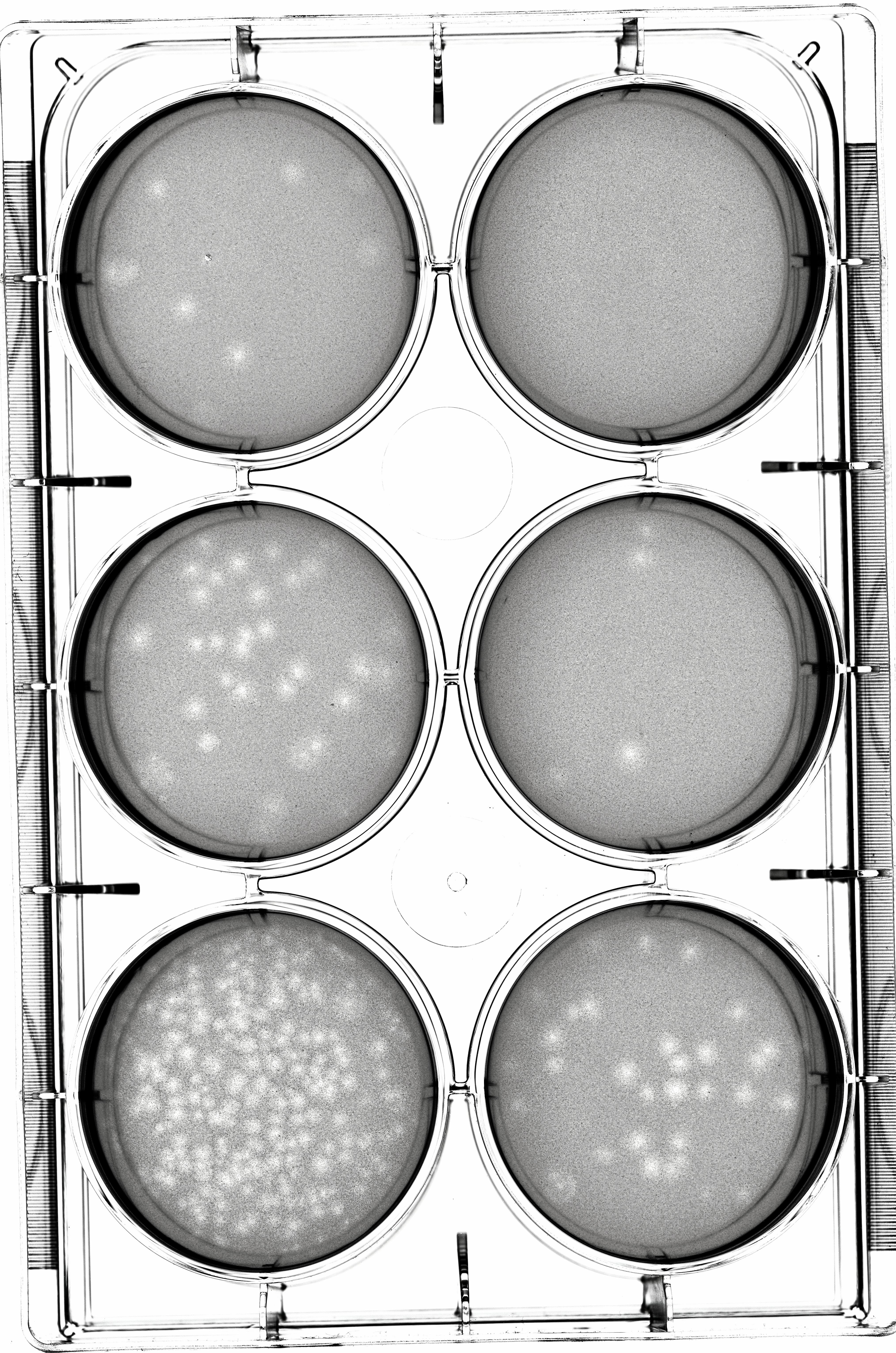

Supplement: S3 File — (ZIP) [file pone.0354311.s003.zip › Figure 2/Fig 2C_plaque assay_miR-125b inhibits PRRSV in PAMs/Fig 2C_plaque assay image_miR-125b inhibits PRRSV in PAMs.jpg]

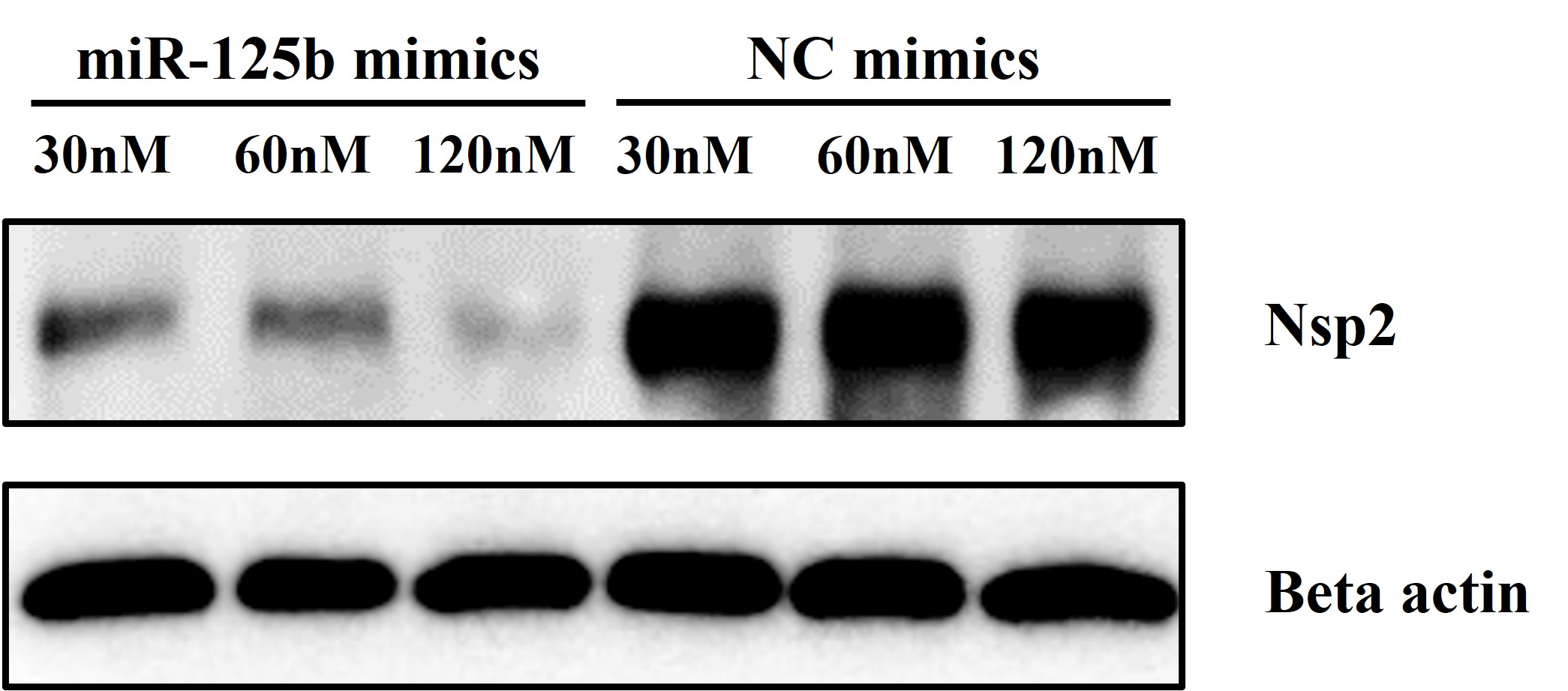

Supplement: S4 File — (ZIP) [file pone.0354311.s004.zip › Later repeat experiments/Figrue 2B/Fig 2B.jpg]

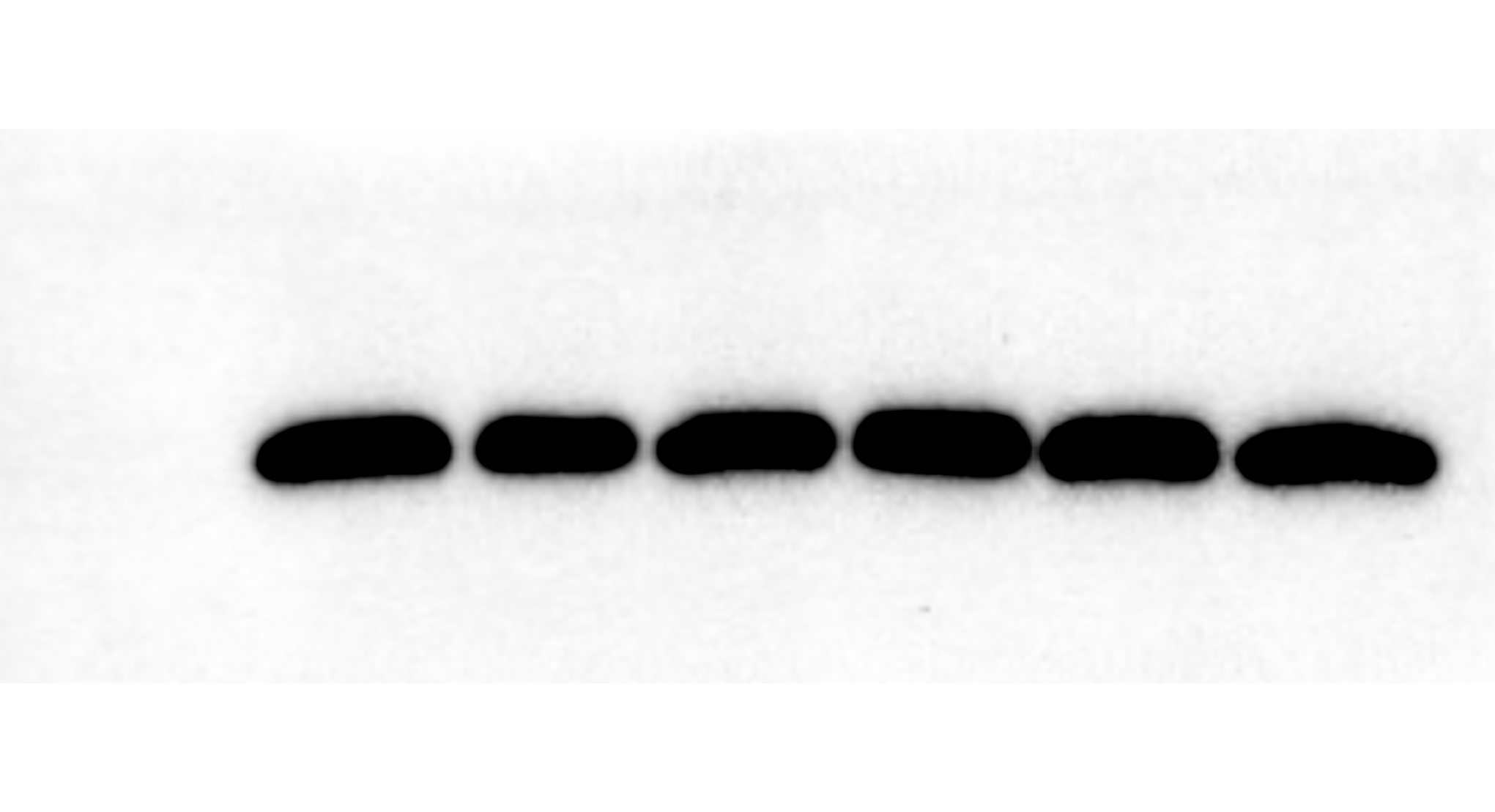

Supplement: S4 File — (ZIP) [file pone.0354311.s004.zip › Later repeat experiments/Figrue 2B/Fig 2B_Beta actin blot.tif]

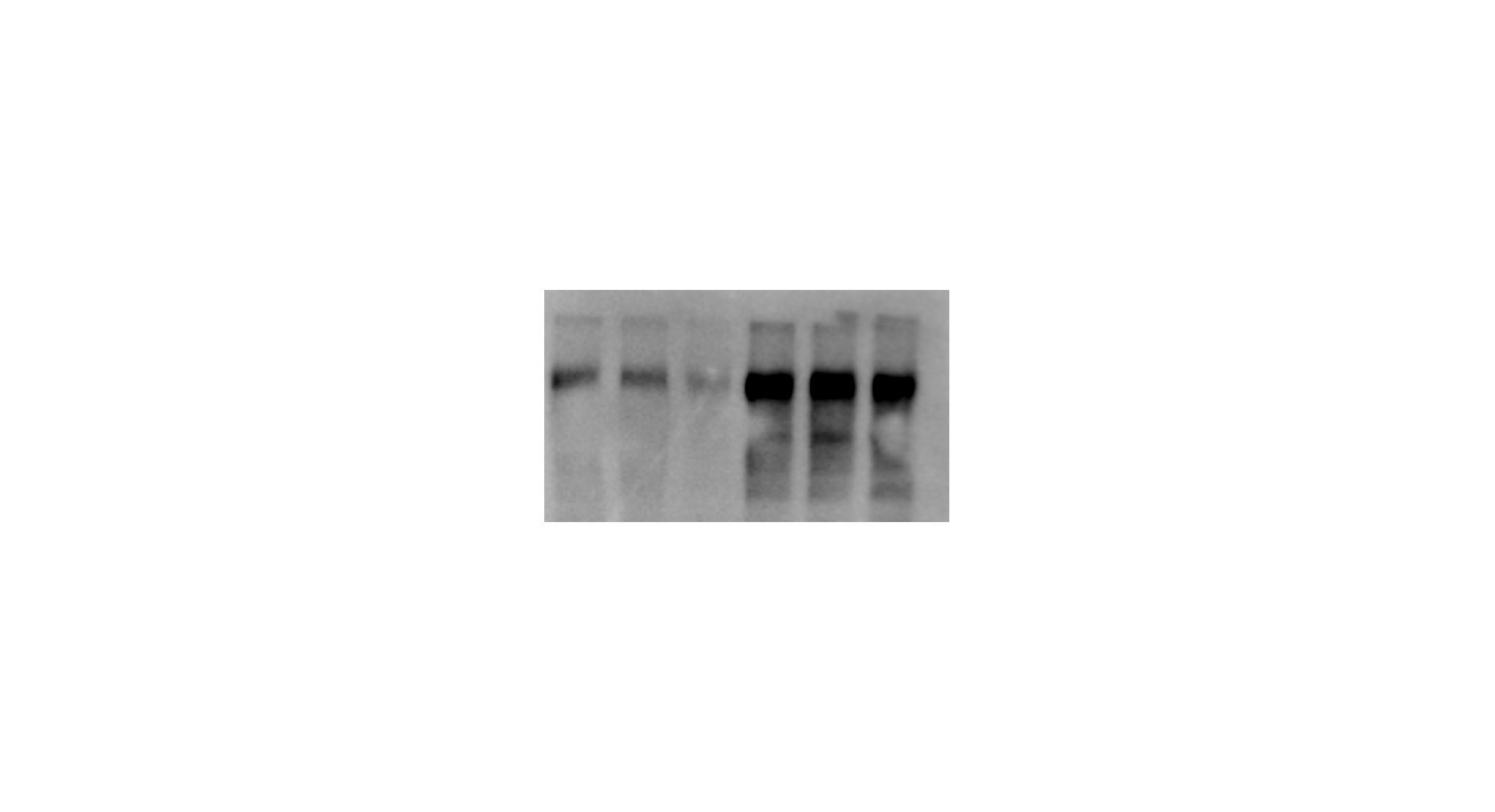

Supplement: S4 File — (ZIP) [file pone.0354311.s004.zip › Later repeat experiments/Figrue 2B/Fig 2B_Nsp2 blot.tif]

## Slide 1
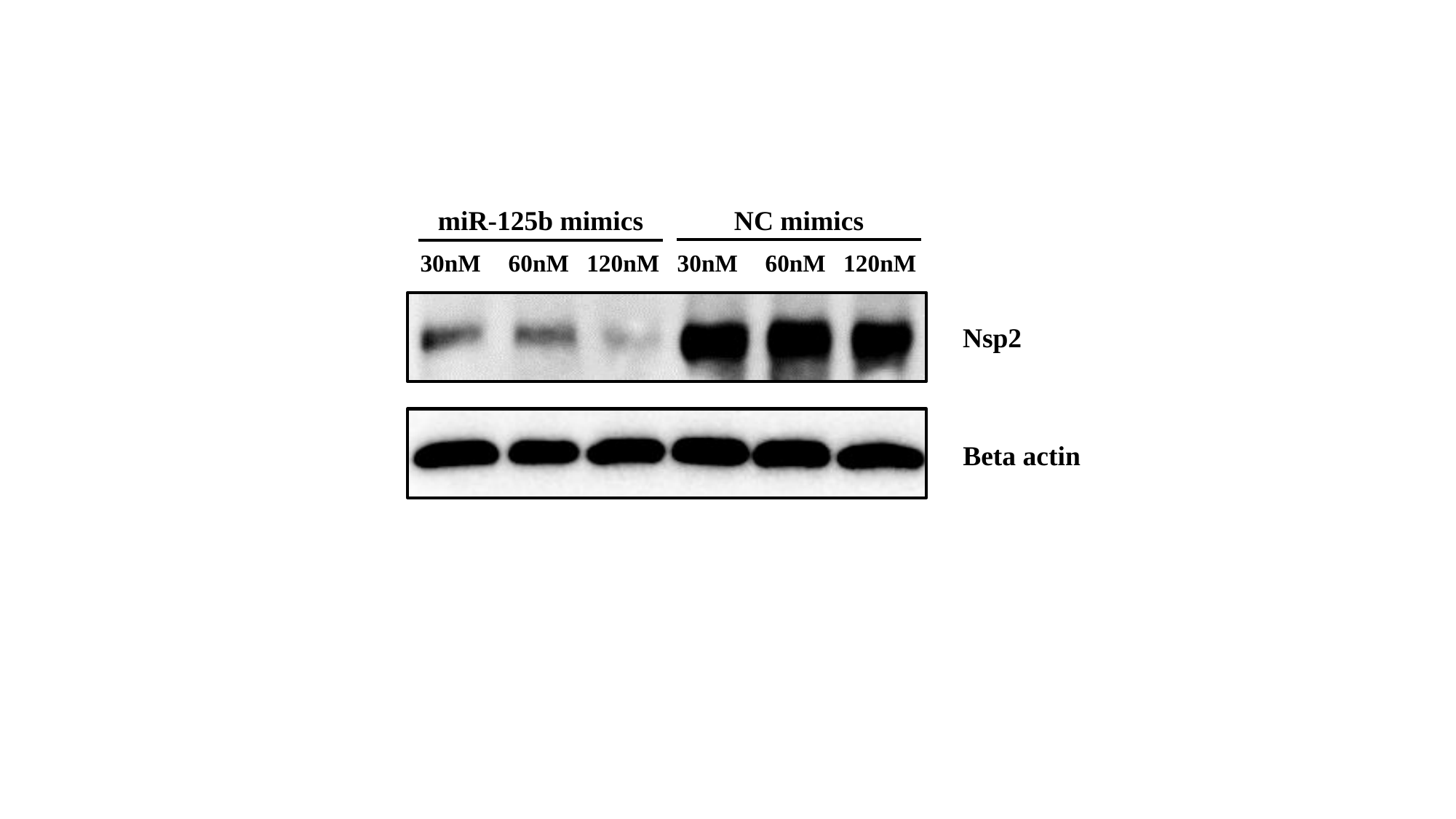

miR-125b mimics
NC mimics
30nM
60nM
120nM
30nM
60nM
120nM
Nsp2
Beta actin

Supplement: S4 File — (ZIP) [file pone.0354311.s004.zip › Later repeat experiments/Figrue 2B/Fig 2B_blot_description.pptx]

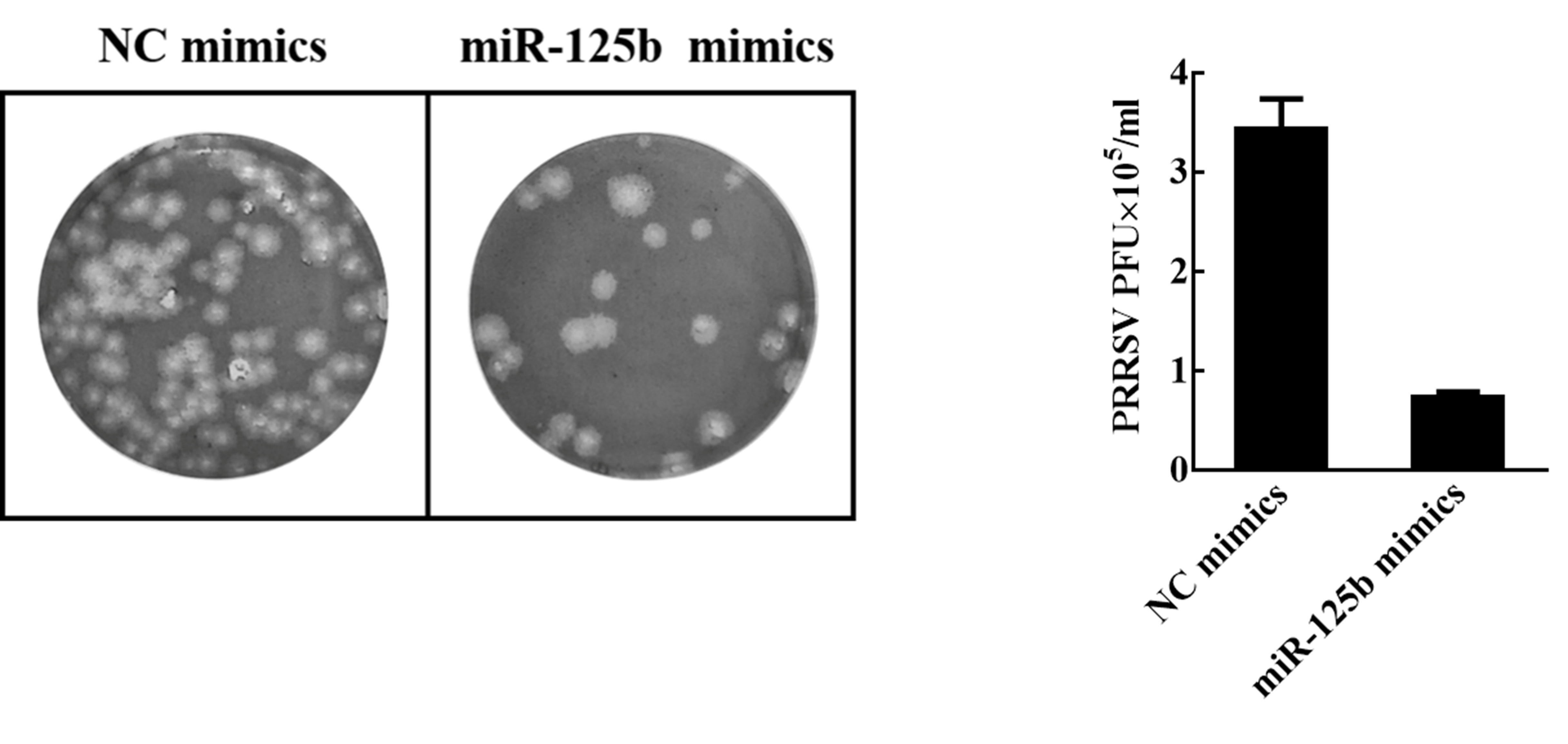

Supplement: S4 File — (ZIP) [file pone.0354311.s004.zip › Later repeat experiments/Figure 2C/Fig 2C.tif]

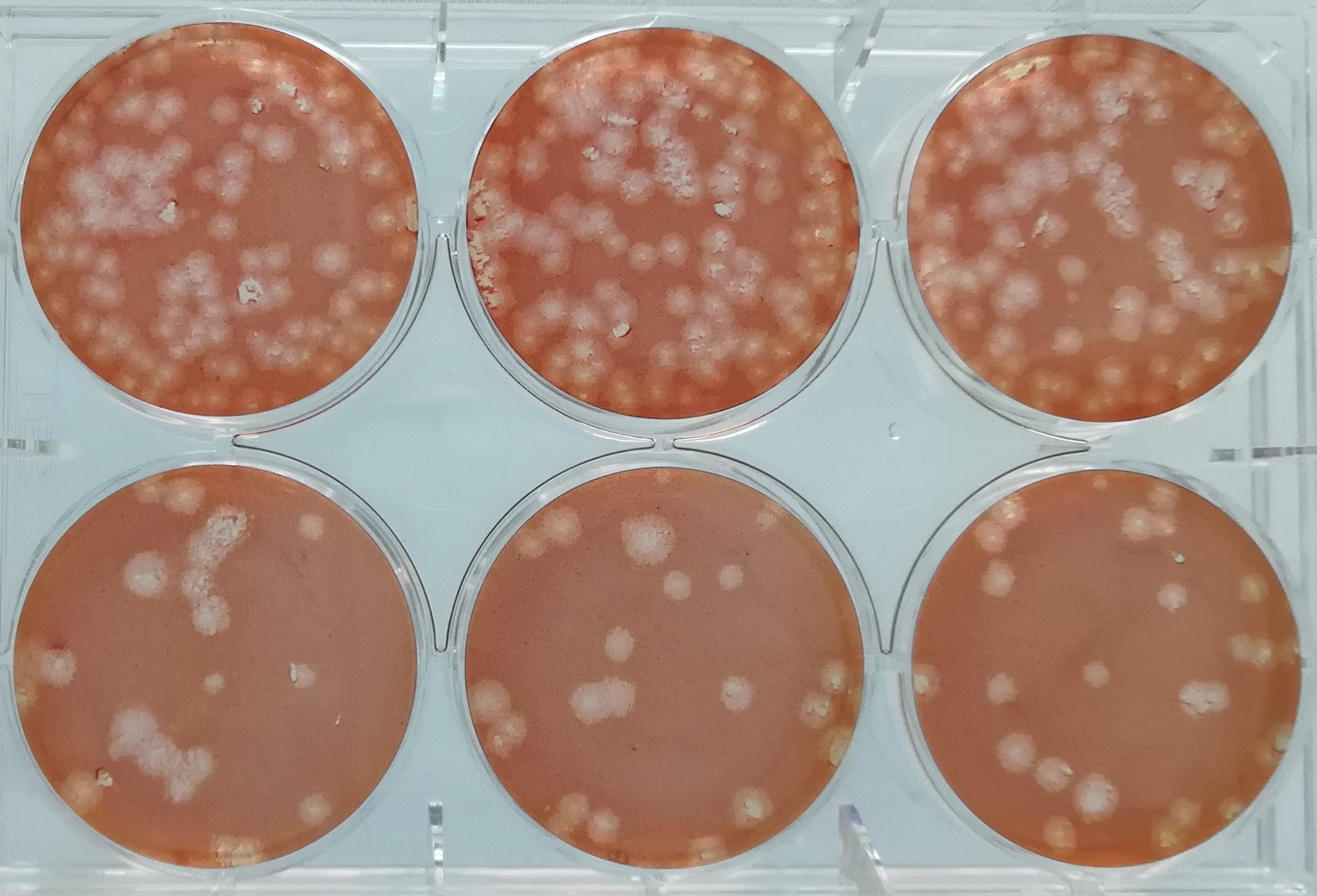

Supplement: S4 File — (ZIP) [file pone.0354311.s004.zip › Later repeat experiments/Figure 2C/Fig 2C_plaque assay image.jpg]

## Slide 1
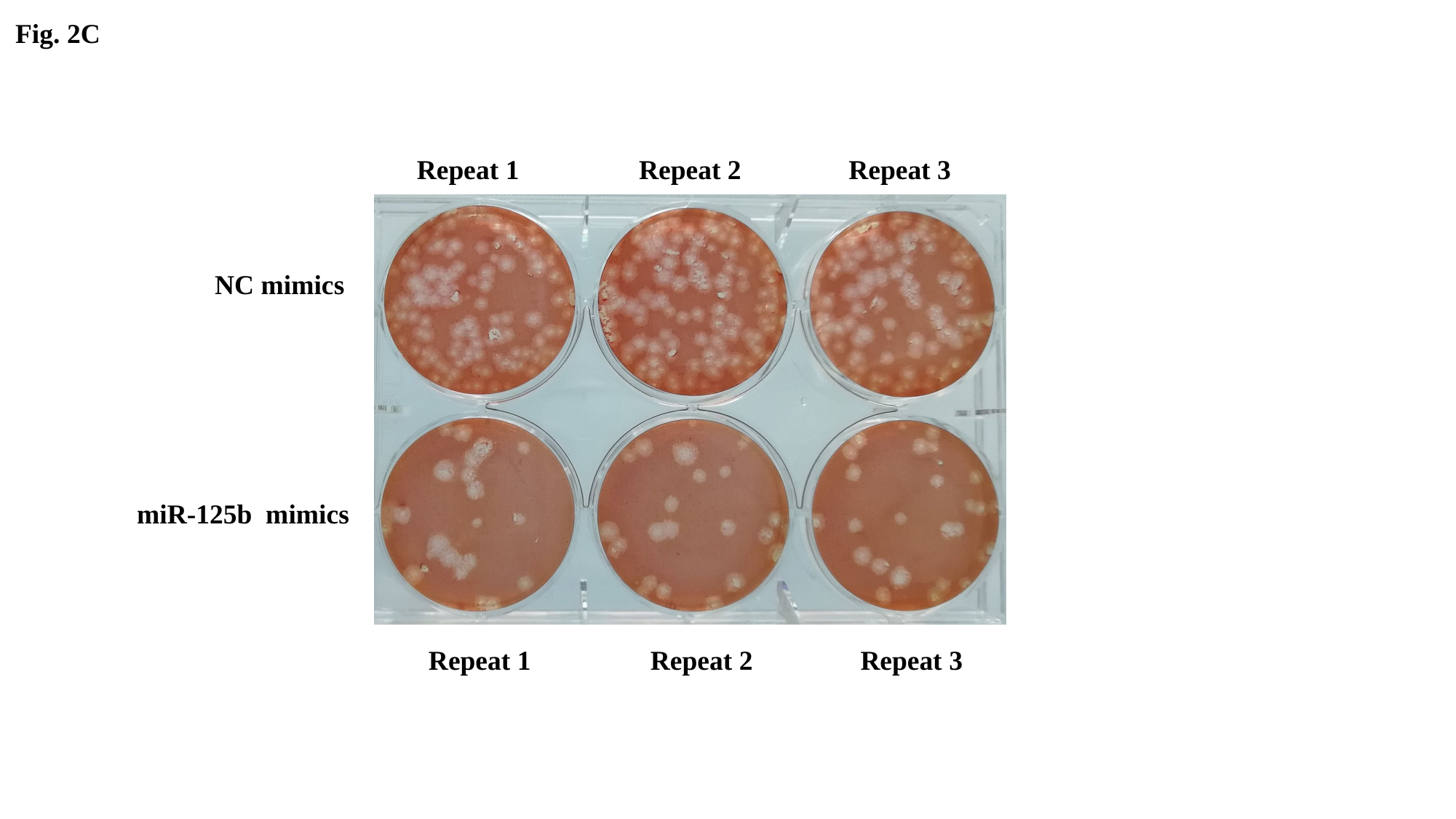

Fig. 2C
Repeat 1
Repeat 2
Repeat 3
NC mimics
 miR-125b mimics
Repeat 1
Repeat 2
Repeat 3

Supplement: S4 File — (ZIP) [file pone.0354311.s004.zip › Later repeat experiments/Figure 2C/Fig 2C_plaque assay image_description.pptx]

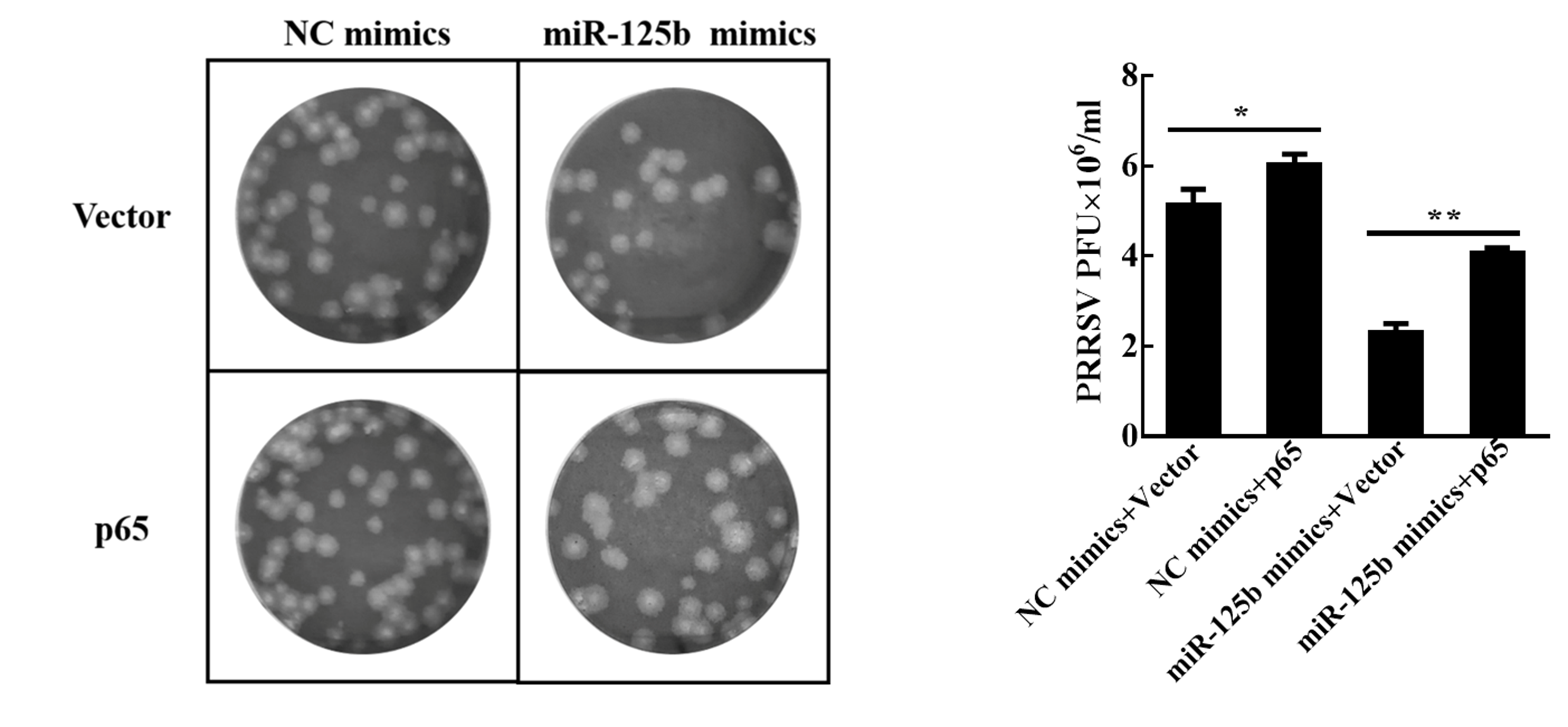

Supplement: S4 File — (ZIP) [file pone.0354311.s004.zip › Later repeat experiments/Figure 6A/Fig 6A.tif]

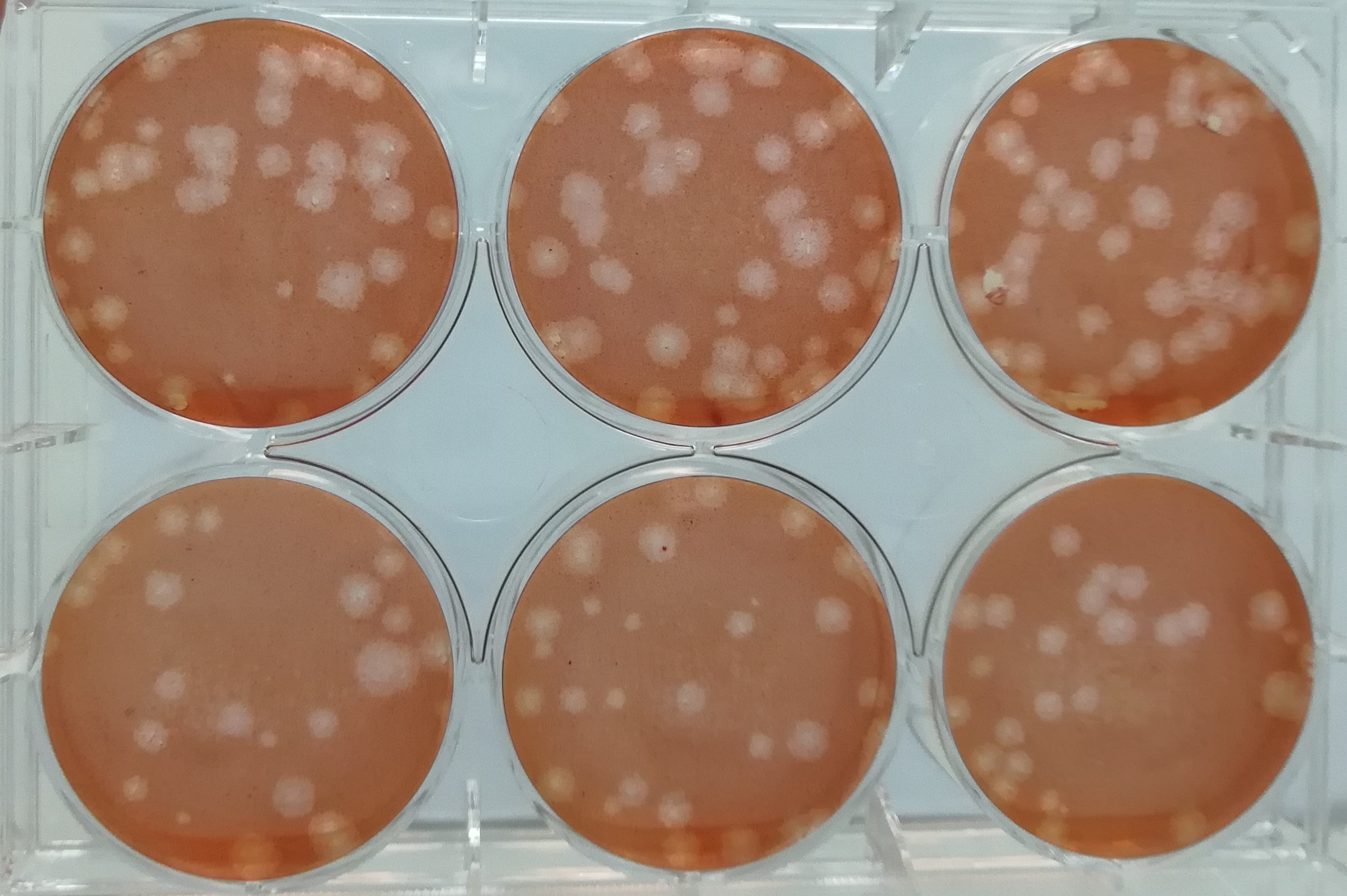

Supplement: S4 File — (ZIP) [file pone.0354311.s004.zip › Later repeat experiments/Figure 6A/Fig6A_plaque assay image-1.jpg]

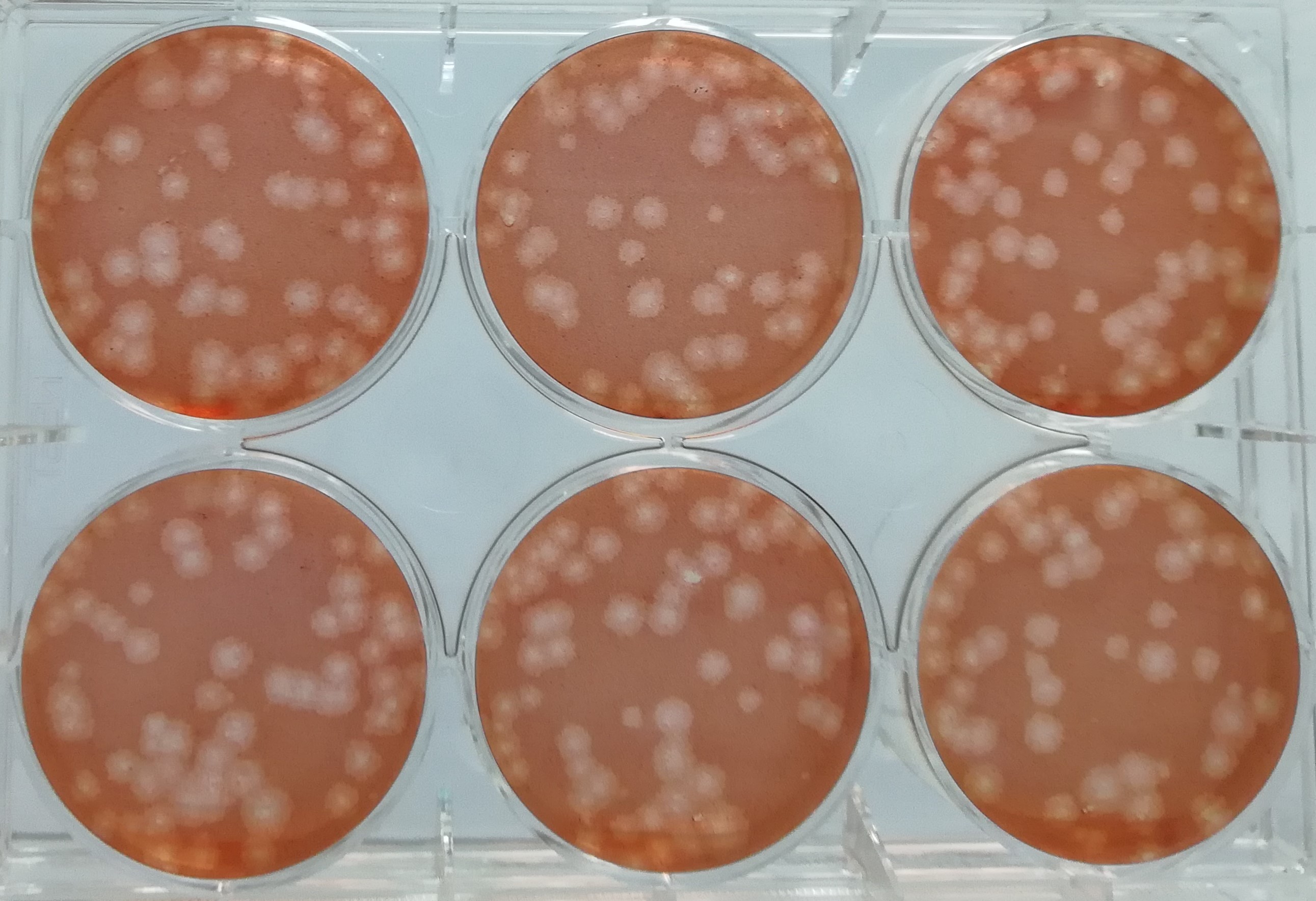

Supplement: S4 File — (ZIP) [file pone.0354311.s004.zip › Later repeat experiments/Figure 6A/Fig6A_plaque assay image-2.jpg]
