# Supplementary material for: Expression of Concern: MiR-125b Reduces Porcine Reproductive and Respiratory Syndrome Virus Replication by Negatively Regulating the NF-κB Pathway
Source: PLoS One. 2026 Jul 22;21(7):e0354311. doi: 10.1371/journal.pone.0354311 (PMC13390827; doi:10.1371/journal.pone.0354311)
Supplement: S4 File — (ZIP) [file pone.0354311.s004.zip › Later repeat experiments/Figure 6A/Fig 6A_plaque assay image_description.pptx]

## Slide 1
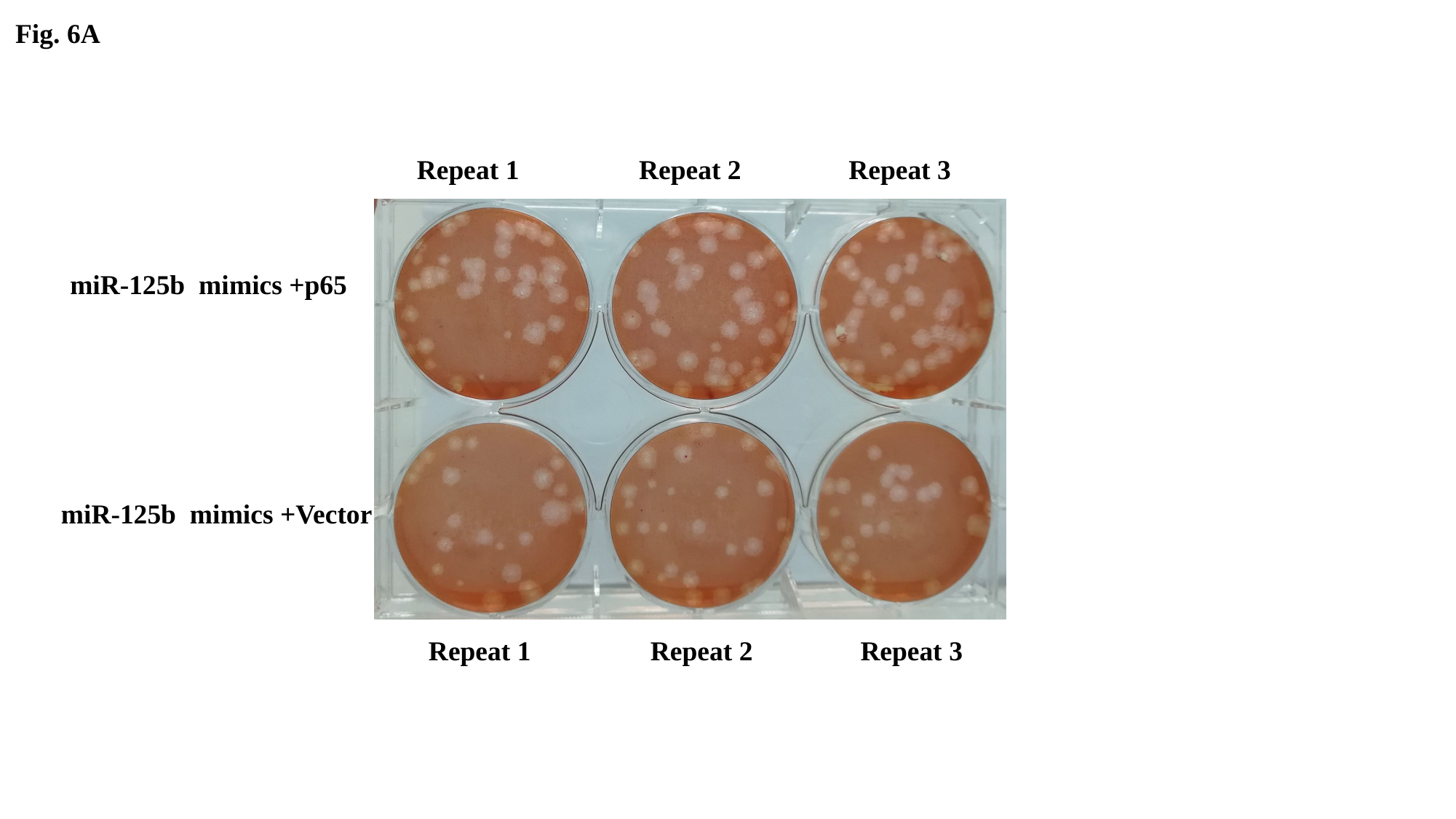

Fig. 6A
Repeat 1
Repeat 2
Repeat 3
miR-125b mimics +p65
 miR-125b mimics +Vector
Repeat 1
Repeat 2
Repeat 3

## Slide 2
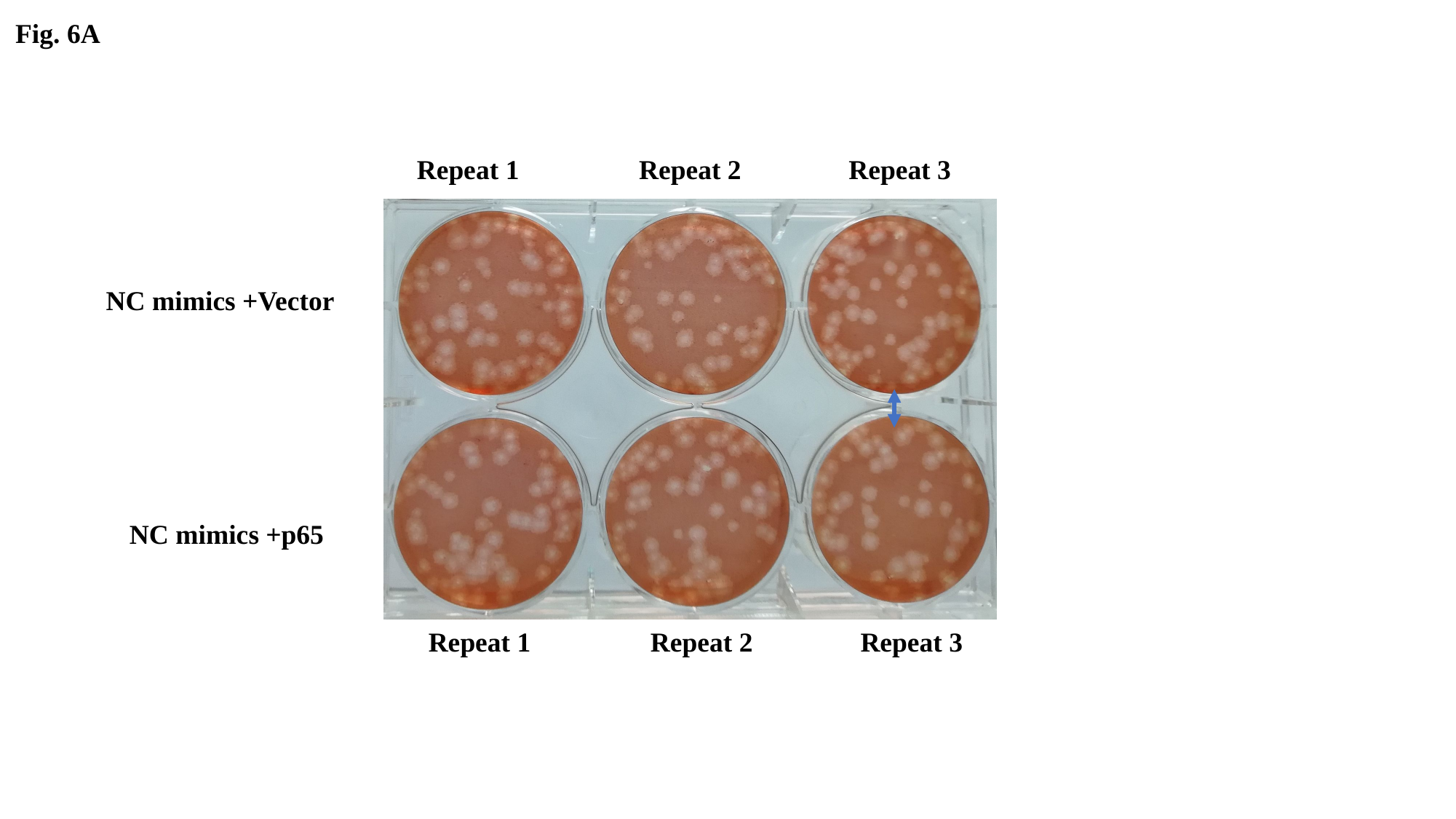

Fig. 6A
Repeat 1
Repeat 2
Repeat 3
NC mimics +Vector
NC mimics +p65
Repeat 1
Repeat 2
Repeat 3
