# Supplementary material for: Expression of Concern: MiR-125b Reduces Porcine Reproductive and Respiratory Syndrome Virus Replication by Negatively Regulating the NF-κB Pathway
Source: PLoS One. 2026 Jul 22;21(7):e0354311. doi: 10.1371/journal.pone.0354311 (PMC13390827; doi:10.1371/journal.pone.0354311)
Supplement: S6 File — (DOCX) [file pone.0354311.s006.docx]

**Target Application Primer**

miR-125b RT 5'-GTCGTATCCAGTGCGTGTCGTGGAGTCGG

CAATTGCACTGGATACGACTCACAA-3'

miR-125b PCR 5'-CGCGCTCCCTGAGACCCTAAC-3'
